# Understanding the molecular mechanisms underlying the effects of light intensity on flavonoid production by RNA-seq analysis in *Epimedium pseudowushanense* B.L.Guo

**DOI:** 10.1371/journal.pone.0182348

**Published:** 2017-08-07

**Authors:** Junqian Pan, Haimei Chen, Baolin Guo, Chang Liu

**Affiliations:** 1 Key Laboratory of Bioactive Substances and Resources Utilization of Chinese Herbal Medicine from Ministry of Education, Beijing, P.R. China; 2 Institute of Medicinal Plant Development, Chinese Academy of Medical Sciences, Peking Union Medical College, Beijing, P.R. China; Universidade de Lisboa Instituto Superior de Agronomia, PORTUGAL

## Abstract

*Epimedium pseudowushanense B*.*L*.*Guo*, a light-demanding shade herb, is used in traditional medicine to increase libido and strengthen muscles and bones. The recognition of the health benefits of Epimedium has increased its market demand. However, its resource recycling rate is low and environmentally dependent. Furthermore, its natural sources are endangered, further increasing prices. Commercial culture can address resource constraints of it.Understanding the effects of environmental factors on the production of its active components would improve the technology for cultivation and germplasm conservation. Here, we studied the effects of light intensities on the flavonoid production and revealed the molecular mechanism using RNA-seq analysis. Plants were exposed to five levels of light intensity through the periods of germination to flowering, the flavonoid contents were measured using HPLC. Quantification of epimedin A, epimedin B, epimedin C, and icariin showed that the flavonoid contents varied with different light intensity levels. And the largest amount of epimedin C was produced at light intensity level 4 (I4). Next, the leaves under the treatment of three light intensity levels (“L”, “M” and “H”) with the largest differences in the flavonoid content, were subjected to RNA-seq analysis. Transcriptome reconstruction identified 43,657 unigenes. All unigene sequences were annotated by searching against the Nr, Gene Ontology, and Kyoto Encyclopedia of Genes and Genomes (KEGG) databases. In total, 4008, 5260, and 3591 significant differentially expressed genes (DEGs) were identified between the groups L vs. M, M vs. H and L vs. H. Particularly, twenty-one full-length genes involved in flavonoid biosynthesis were identified. The expression levels of the flavonol synthase, chalcone synthase genes were strongly associated with light-induced flavonoid abundance with the highest expression levels found in the H group. Furthermore, 65 transcription factors, including 31 FAR1, 17 MYB-related, 12 bHLH, and 5 WRKY, were differentially expressed after light induction. Finally, a model was proposed to explain the light-induced flavonoid production. This study provided valuable information to improve cultivation practices and produced the first comprehensive resource for *E*. *pseudowushanense* transcriptomes.

## 1. Introduction

The shade plant, *Epimedium pseudowushanense* B.L.Guo belong to the genus *Epimedium* (Chinese name, Yin Yang Huo) from the Berberidaceae family. This genus contains 58 species [1]. Among them, *Epimedium brevicornum* Maxim, *Epimedium sagittatum* (Sieb. et Zucc.) Maxim, *Epimedium pubescens* Maxim, *Epimedium wushanense* T.S.Ying, and *Epimedium koreanum* Nabai were considered authentic sources of pharmacological products (2015 Chinese pharmacopeia). Materials from *Epimedium* plants have been used to invigorate sexuality and to strengthen muscles and bones [1]. They are of significant economic importance as the annual sale value of medicinal products containing active components of *Epimedium* is estimated to exceed 1.1 billion Chinese Yuan in China (personal communications). *E*. *pseudowushanense* B.L.Guo is one the species most similar to *E*. *wushanense* in terms of morphology and chemical components. Due to its many favorable agricultural properties, *E*. *pseudowushanense* has been cultivated widely and used extensively as a substitute of *E*. *wushanense*. Improvement of its cultivation efficiency remains an active area of research.

Active components of *Epimedium* plants largely consist of flavonoids, particularly prenylated flavonol glycosides. Well-known compounds include epimedin A, epimedin B, epimedin C, and icariin. Previous studies have revealed significant therapeutic effects of these compounds on breast cancer, liver cancer, and leukemia [2–4]. With the increased demand of active components from *Epimedium* and the low recycling rate of these plants, increasing the production of the active compounds through valid commercial culture and metabolic engineering has become an active area of research. Based on the previous study we found that light could influence the content of *Epimedium pseudowushanense* B.L.Guo[5]. So we should research the molecular mechanisms underlying the effects of light intensity on flavonoid production of it. This study could help us to know why the flavonoid content changed under different light conditions. Flavonoids are a remarkably large group of plant secondary metabolites that are derived from phenylalanine. The flavonoid biosynthetic pathway is one of the best most studied pathways of plant secondary metabolites. Many structural gene encoding enzymes involved in this pathway have been isolated and well characterized from several model species such as *Arabidopsis*, maize, and grape.[6]

Our study intends to investigate the effects of one of the most important environmental factor, light, on the production of its active components, flavonoids in *E*. *pseudowushanense*. Furthermore, we would like to identify the optimal light intensity for maximal flavonoid accumulation. Last, we exploited RNA-seq technology to understand the underlying molecular mechanisms. The success of this study would not only determine the optimal conditions for cultivation and flavonoid production, but also identify the genes responsible for flavonoid biosynthesis and regulation.

RNA sequencing (RNA-seq) technology uses next-generation sequencing (NGS) to reveal the presence and quantity of RNAs in a biological sample under a particular condition [7]. Given its high-throughput capability, RNA-seq can detect low-abundance genes with sufficient sensitivity [7,8]. RNA-seq has been widely used for gene discovery, differential gene expression analysis, single nucleotide polymorphism discovery, and SSR discovery [7,9]. NGS technology has been applied to identify genes in *Epimedium* species in recent years. For example, analysis of the leaf transcriptome of *E*. *sagittatum* through 454 GS-FLX pyrosequencing led to the discovery of many genes involved in flavonoid biosynthesis [10].

Light is an important environmental factor that can induce plant growth, development and the biosynthesis of secondary metabolites and stimulate the accumulation of these compounds in plants [11]. Changes in light intensity may influence flavonoid content because the flavonoid hydroxyl groups on the A and B rings vary in number and position. Several studies have shown that high light irradiance promotes the biosynthesis of flavonoids, such as dihydroxy B-ring-substituted flavonoids (luteolin 7-O- and quercetin 3-O-glycosides) but does not influence the biosynthesis of monohydroxy B-ring-substituted flavonoids (pigenin 7-O- and kaempferol 3-O-glycosides) [12–15]. Pacheco [16] reported that *Piper aduncum* grown under 50% natural light irradiance had higher total flavonoid concentration than those grown under 100% natural irradiance. Deng and others[17] found that *Cyclocarya paliurus* under 100% natural light had higher kaempferol, quercetin and isoquercitrin than 50% and 15% natural light.

The effects of light are likely to be mediated through the upregulation of the expression of genes involved in the secondary metabolite biosynthesis. For example, light can promote the upregulation of genes involved in the biosynthesis and accumulation of flavonoids in *Catharanthus roseus* and *Ligustrum vulgare* [18,19]. In the study of Azumaet [20], light treatment led to induced higher expression levels of CHS, CHI, F3H, flavonoid 3’,5’-hydroxylase (F3’5’H), DFR, O-methyltransferase (OMT) as well as UFGT compared to dark grown berries. Pacheco [16] reported that *Piper aduncum* grown under 50% natural light irradiance had higher PAL expression than others. Leyva [21] also found that the regulation of CHS was up with the increased light intensity in Arabidopsis thaliana.

Based on the information described above, we hypothesize that (1) the accumulation of flavonoid is induced by light in an intensity dependent manner; (2) the induction is mediated by the differential expression of genes involved in the biosynthesis of the active components, flavonoids. To test this hypothesis, we first treated the plants with different light intensity levels. Second, we determined the abundance of the flavonoid contents with HPLC. Third, we compared the flavonoid abundance against the light intensity to identify the optimal levels. Forth, we selected plant materials treated at three levels with lowest, middle and highest levels of flavonoids for RNA-seq analysis. Fifth, analysis of the RNA-seq results identified genes involved in flavonoid biosynthesis and differential expressed genes (DEGs) between different light treatment groups. Last, models were proposed to explain the light-induced flavonoid accumulation.

## 2. Materials and methods

### 2.1 Plant materials and growth conditions

Ninety 2-year-old healthy *E*. *pseudowushanense* plants were collected from Lei Shan County (16° N, 108° E) in Guizhou Province. The plants were transferred to plastic pots (10 cm × 10 cm for inner diameter and height, 1 plant per pot) filled with a substrate mixture of 75% peat and 25% vermiculite, and then placed in the greenhouse of the Institute of Medicinal Plant Development on March 1, 2015. The plants were randomly subjected to radiation with five level I1 (5.5 ± 2.5 μmol· m^−2^·s^−1^), I2 (14.5 ± 2.5μmol· m^−2^·s^−1^), I3 (18.2 ± 2.5 μmol· m^−2^·s^−1^), I4 (54.6 ± 2.5 μmol· m^−2^·s^−1^), and I5 (90.9 ± 2.5μmol· m^−2^·s^−1^) light intensities for 16 h per day (T5-fluorescent lamps were used as the light resource, and there were 30 pots per level). A 20–21°C temperature range was set for entire cultivation, and humidity was maintained at 60%. Except for the light intensity, the other culture conditions are same at each pot. To control the light intensity is the same for all plants in each light treat level, the thin paper were used which eliminated the effect of light from outside. The light conditions were confirmed by Li-6400 external quantum sensor (LI-COR, Lincoln, NE, USA) system. After treatment for 30 days, the plants in each group were further divided into three subgroups with 10 plants each. Fresh leaves from plants belonging to the same subgroups were randomly collected, pooled, and then stored in liquid nitrogen until use.

### 2.2 Profiling of chemical compositions using HPLC

*E*. *pseudowushanense* leaf powder (200 mg) was passed through a No. 3 pharmacopoeia sieve (Each treatment group had 30 plants, they were divided into three sub groups, with 10 plants. The sub group leaves were mixed and each treatment group had 3 biological replications) and then extracted with 50 mL of 70% EtOH by ultrasonication at room temperature for 30 min. The solution was passed through a 0.45 μm microfiltration membrane, and a 20 μL aliquot of the filtrate was injected into HPLC for analysis. HPLC separation was performed on a Zorbax SB-C18 column (Agilent Technologies, Palo Alto, CA, USA) (5 μm, 250 mm × 4.6 mm). Eluents A and B were water and acetonitrile, respectively. The gradient elution program was as follows: 0–17 min (25%–26% B) and 17–26 min (26%–100% B). The column was washed with 100% eluent B for 15 min between every two testing samples and then re-equilibrated with 25% eluent B for 10 min. The elution was performed under the following conditions: flow rate, 1.0 mL/min; column temperature, 25°C; and detection wavelength, 270 nm. Data processing was performed using PerkinElmer ChemStation software (version 6.3.1).

### 2.3 RNA isolation and quantification

For RNA-seq experiments, plant samples from two subgroups of each treatment group were subjected to total RNA extraction using the RNAprep Pure Plant Kit (Polysaccharides and Polyphenolics-rich) (Cat No. DP441, TianGene, China). RNA degradation and contamination were monitored using GeneGreen-stained 1% agarose gels, and RNA purity was determined using a NanoPhotometer^®^ spectrophotometer (IMPLEN, Westlake Village, CA). RNA concentration was measured using Qubit^®^ RNA Assay Kit in Qubit 2.0 Fluorometer (Life Technologies, Foster City, CA), and RNA integrity was assessed using the RNA Nano 6000 Assay Kit of a Bioanalyzer 2100 system (Agilent Technologies, Santa Clara, CA).

### 2.4 RNA-seq library construction and sequencing

The sequencing libraries were constructed using the NEBNext^®^ Ultra^™^ RNA Library Prep Kit for Illumina (NEB, USA) in accordance with the manufacturer’s protocol. In brief, mRNA was purified from total RNA using poly-T oligo-attached magnetic beads. Fragmentation was carried out using divalent captions under elevated temperature in the NEBNext First-Strand Synthesis Reaction Buffer (5×). First-strand cDNA was synthesized using a random hexamer primer and M-MuLV Reverse Transcriptase (RNase H). Subsequently, second-strand cDNA was synthesized using DNA Polymerase I and RNase H. The remaining overhangs were converted into blunt ends via exonuclease/polymerase activities, and the enzymes were removed. After adenylation of 3′ends of DNA fragments, NEBNext Adaptor with a hairpin loop structure was ligated to the cDNA fragments, which were then purified, end-repaired, A-tailed, and then ligated to index adapters (NEB). The templates were amplified by PCR and then sequenced on an Illumina Hiseq^™^ 2500 platform, which led to the generation of 125 bp paired-end reads. Data analysis and base calling were performed using Illumina instrument software. DNA sequencing was performed at Beijing Ori-Gene Science and Technology Co., Ltd. Raw data had been deposited in the Short Read Archive of GenBank with the accession numbers: xxx (to be provided).

### 2.5 De novo assembly and function annotation

Raw sequencing reads were processed with SolexaQA (http://solexaqa.sourceforge.net/) to filter out low-quality reads with default parameters and short reads with length ≤ 60 bp. The resulting high-quality RNA-seq data from the libraries were assembled using the computer program Trinity [22]. In case several transcripts were identified for the same gene, the longest transcript was selected as the representative sequence of the gene and will be called unigene sequence in the following text. For functional annotation, all unigene sequences were searched against several databases, including the NCBI non-redundant protein sequences (Nr, ftp://ftp.ncbi.nlm.nih.gov/blast/db/FASTA/nr.gz), Gene Ontology (GO http://www.geneontology.org/), Swiss-Prot/Trembl (http://www.uniprot.org/), Pfam (http://pfam.xfam.org/), and Kyoto Encyclopedia of Genes and Genomes (KEGG; http://www.genome.jp/kegg/), by using the program BLASTX with E value ≤ 1e^−5^ and percentage of similarity ≥ 30%.

### 2.6 Gene expression quantification and differential gene expression analysis

To estimate the abundance of the transcripts, all transcripts assembled by Trinity were treated as the reference sequences. The clean reads were then mapped to the reference sequences using TopHat (version 2.0.10, http://tophat.cbcb.umd.edu/) with default parameters. The program Cuffdiff (version 2.2.1,(http://cuffdiff.cbcb.umd.edu/) was used to calculate the expression levels of genes and transcripts in terms of reads per kilobases per million reads (RPKM) and the p-value for differentially expressed genes (DEGs) based on two-tailed unpaired Student’s t-test. Genes with the number of mapped reads ≥ 10, fold change ≥ 2, and uncorrected p ≤ 0.05 were deemed significant DEGs.

### 2.7 Enrichment analysis

GO enrichment analysis was conducted using GOseq [23]. We identified the significantly enriched GO term of DEGs with corrected p ≤ 0.05. For KEGG analysis, we used the KEGG pathway as a unit and applied the hyper geometric test to find significantly enriched pathways [24]. We identified the significantly enriched KEGG pathway of DEGs with corrected p ≤ 0.05.

### 2.8 Identification of transcription factors in *E*. *pseudowushanense*

Gene-encoding transcription factors were identified by comparing all unigene sequences against the plant transcription factor database (PlnTFDB; http://plntfdb.bio.uni-potsdam.de/v3.0/downloads.php) using BLASTX with a cutoff E value of 1e^-5^ [25].

### 2.9 Validation of RNA-seq experiments

The RNA samples used for RNA-seq analyses were subjected to reverse transcription quantitative real-time PCR (RT-qPCR) analysis. Each experiment was conducted with three technical replicates. For each sample, reverse transcription was performed on 1 μg total RNA by TransScript One-Step gDNA Removal and cDNA Synthesis SuperMix (TransGen) in a 20 μl volume with anchored oligo(dT)18 primer. The reaction was carried out at 42°C for 15 min and 80°C for 5 s using an ABI 7500 Fast instrument (Applied Biosystems). Gene-specific primers were designed using PrimerQuest (http://www.idtdna.com/Primerquest/Home/Index). The primers used in this study are listed in S1 Table. The actin gene was chosen as the endogenous control. Each qPCR reaction contained 10 μL of 2× TransStart^®^ Top Green qPCR SuperMix (TransGen), 25 ng of cDNA sample, and 200 nM gene-specific primers in a final volume of 20 μL. The cycling conditions were 94°C for 30 s, followed by 40 cycles of 94°C for 5 s and then 60°C for 34 s. Melting curve analyses were performed to verify the specificity by ABI 7500 Fast instrument. The relative expression levels were calculated using the 2^–ΔΔCt^ method [26].

### 2.10 Sequence analysis

For selected proteins, homologous sequences were retrieved from Genbank with an E value cutoff of 1e^−5^. The sequences were then aligned with ClustalW software. Phylogenetic trees were constructed using the neighbor-joining algorithm with MEGA 7.0. The bootstrap score was calculated based on 1000 replications.

### 2.11 Statistical analysis

Correlation coefficients among flavonoid contents, gene expression levels of related enzymes, and transcription factors were calculated using Excel. All values are presented as the mean standard error of the mean. Statistical significance of differences was evaluated using Student’s t-test or ANOVA in SPSS10 software. The significance of pearson correlation was calculated as described by VassarStats (http://www.vassarstats.net/).

## 3. Results

### 3.1. Effects of light intensities on flavonoid content

**Fig 1:**
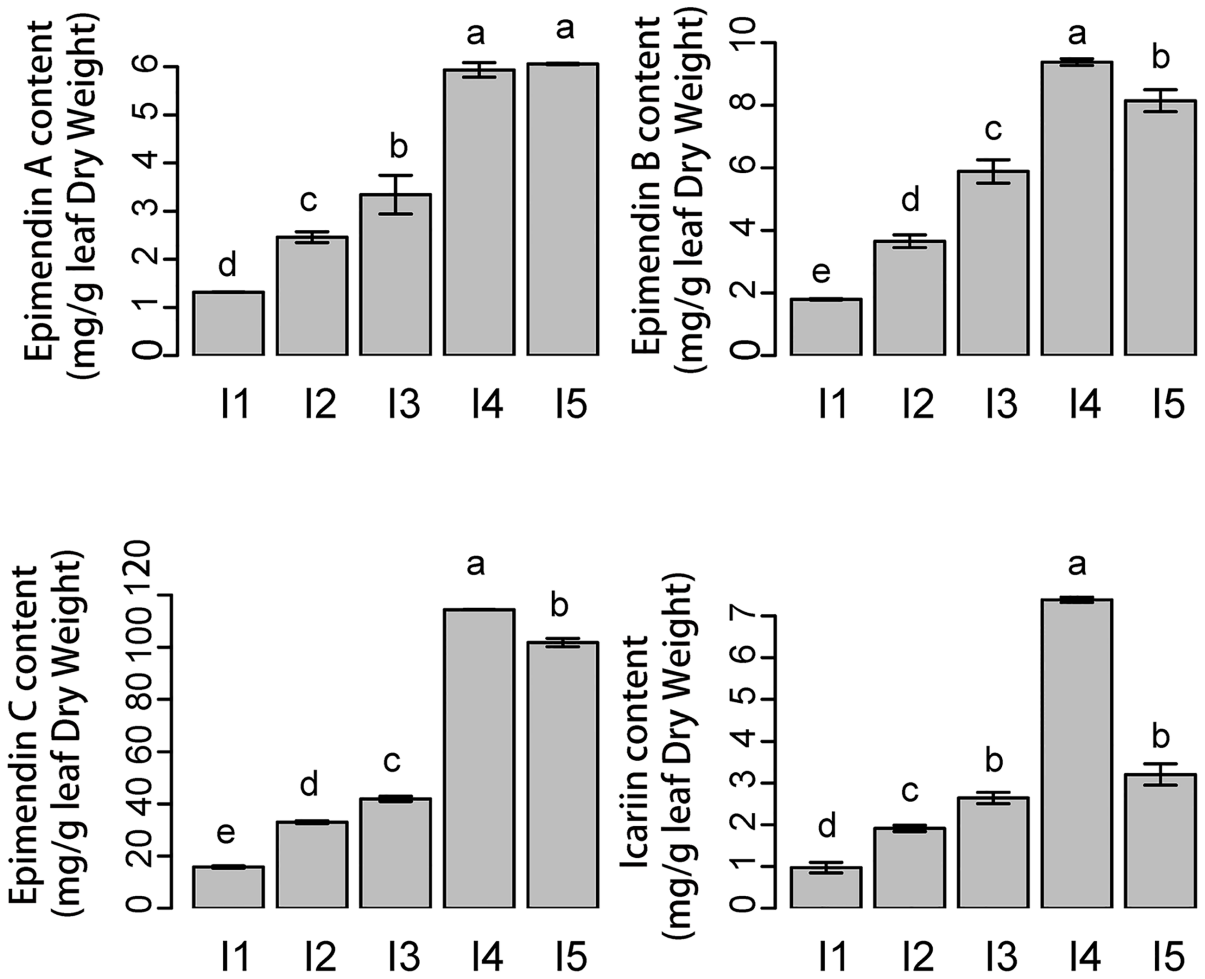
The amounts of Epimedin A, Epimedin B, Epimedin C and Icariin in *E*. *pseudowushanense* under different light intensities. The data are expressed as the mean±SD. Different letters (a, b and c) indicate significant differences among groups with different light intensity treatments (*p*≤0.05; n = 30).

The methodology validated in our previous study was applied to analyze the flavonoid content by HPLC at five light levels [27]. Fig 1 shows the changes in the contents of four different flavonoid glycosides in *E*. *pseudowushanense* under different light intensities. Interestingly, epimedin A showed different changes from epimedin B, epimedin C and icariin at I4 and I5 treatments. Epimedin A content increased as light intensity increased from I1 to I5. Thus, I5 increased epimedin A by 360.6% (p<0.05) compared with I1. Furthermore, epimedin B, epimedin C and icariin amounts showed similar changes. Epimedin B, epimedin C and icariin increased when light intensity increased from I1 to I4, whereas all decreased under I5. The highest epimedin B, epimedin C and icariin contents were observed under I4. Epimedin B, epimedin C and icariin contents were 421.9% (p<0.05), 624.0% (p<0.05) and 659.9% higher, respectively, than under I1.

### 3.2. RNA-seq analysis of E. pseudowushanense treated with different light intensities

In order to explore the molecular mechanism of light-induced flavonoid synthesis and accumulation in *E*. *pseudowushanense*, six cDNA libraries constituting two biological repeats were constructed from three treatment groups which the flavonoid contents were found most significantly different (i.e., the low I1, the middle I3 and the high I4) (Fig 1) and sequenced using Illumina high-throughput sequencing platform. Six samples were named L1, L2 (I1); M1, M2 (I3); H1 and H2 (I4). The RNA-seq results were summarized in Table 1. For the six samples, the total number of raw reads ranged from 45 to 55 million. After removing the adapters, low-quality sequences, and reads shorter than 35 bp, the numbers of clean reads were 40.3, 27.3, 27.0, 28.9, 32.9, and 32.9 million for the six samples, respectively (Table 1). All the clean reads were combined and then assembled into 57,962 contigs by using Trinity. For each unigene, the longest transcript (in case of multiple transcripts) was selected as the representative and was called “unigene sequence.” A total of 43,657 unigene sequences with lengths ranging from 224 bp to 17,683 bp, with an average length of 837 bp and an N50 of 1383 bp, were obtained (S1 File, S2 Table). To assess the quality of our assembly, the clean reads were mapped to unigenes. The ratios of all mapped reads ranged from 80.27% to 90.38%, whereas the ratios of uniquely mapped reads ranged from 71.67% to 83.44% (S3 Table).

**Table 1:** Summary of RNA-seq data from *E*. *pseudowushanense* treated with different light intensities.

Sample Name	Number of Raw Reads	Raw Bases	Clean Reads	Clean Bases	Average length	% of Clean Reads
L_1	50.441 M	6.356 G	40.252 M	4.813 G	119.6 bp	79.80%
L_2	55.118 M	6.945 G	27.337 M	3.205 G	117.3 bp	49.60%
H_1	45.830 M	5.775 G	32.933 M	3.955 G	120.1 bp	71.86%
H_2	45.535 M	5.737 G	32.913 M	3.947 G	119.9 bp	72.28%
M_1	44.538 M	5.612 G	27.020 M	3.240 G	119.9 bp	60.67%
M_2	47.603 M	5.998 G	28.919 M	3.466 G	119.8 bp	60.75%

**Fig 2:**
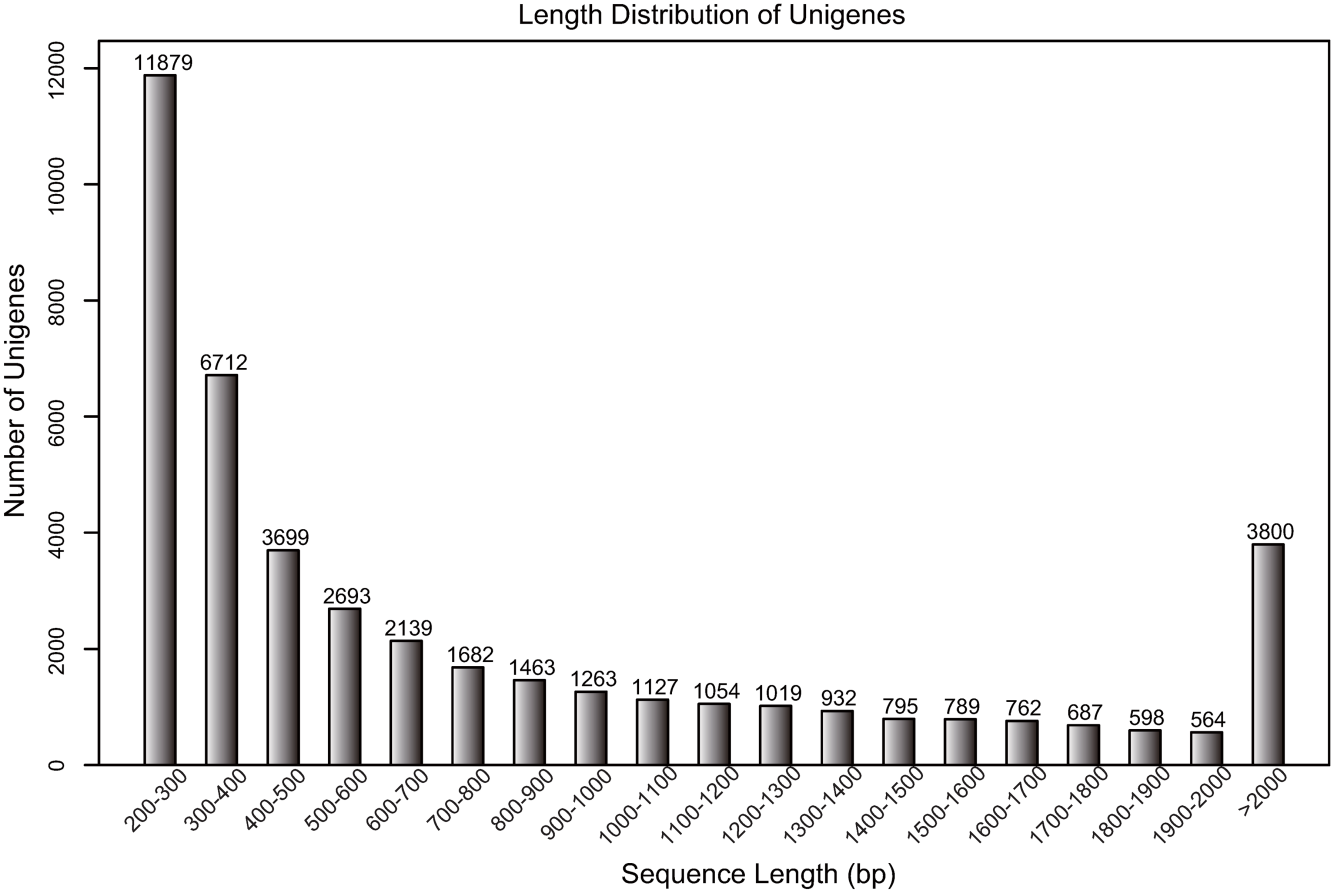
Sequence length distribution of the unigene sequences. The X-axis shows the range of lengths of the transcript sequences. The Y-axis shows the number of unigenes.

We then examined the length distribution of these unigene sequences. In contrast to 12,127 unigene sequences that were longer than 1000 bp, 18,591 unigene sequences had lengths between 200 and 400 bp (Fig 2). We then compared our assembly with those for *E*. *sagittatum* based on information provided in the manuscript. Our assembled transcript was, on average, 1.23 times longer than those of *E*. *sagittatum*. Moreover, the number of genes was 15.3% greater than that of the *E*. *sagittatum* dataset.

To determine the potential functions of these unigene sequences, they were searched against the databases Nr, Nt, Trembl, Swiss-Prot, and Pfam by using BLAST with an E value cutoff of 1e^−5^. The ratios of annotated unigene sequences ranged from 35% to 61% (S4 Table). Among the 43,657 unigenes, 25,989 (59.5%) and 15,441 (35.4%) had at least one significant match with an E value ≤ 1e^−5^ against the Nr and Nt databases. The mapping rates of unigene sequences to the Swiss-prot, Trembl, and Pfam protein databases were 45.2%, 60.1%, and 47.8%, respectively. In terms of the species source of top hit sequences, sequences from *Nelumbo nucifera* represented 36.5% of the top hits of our unigene sequences, followed by *Vitis vinifera* (10.7%), *Ricinus communis* (3.4%), *Theobroma cacao* (2.9%), *Jatropha curcas* (1.9%), and *Populus trichocarpa* (1.6%). This distribution suggests that *N*. *nucifera* is the closest species that has a large number of sequences in the Nr database (S1 Fig).

### 3.3. Functional classification of unigenes

**Fig 3:**
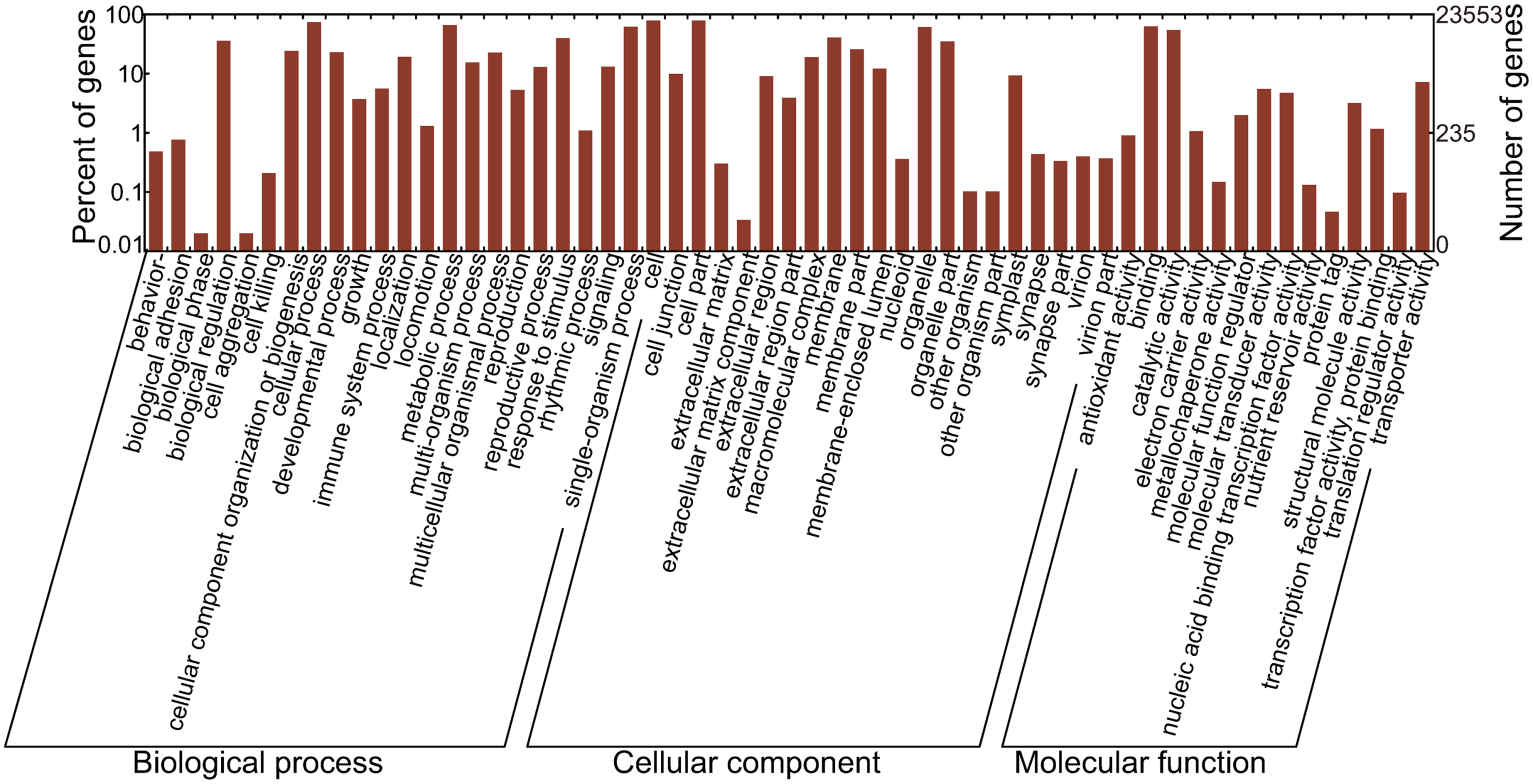
GO classifications of the unigene sequences. Annotated unique sequences were classified into ‘Biological process’, ‘Cellular component’ and ‘Molecular function’ respectively.

We mapped the transcripts to GO terms and KEGG pathways; 23,553 unigene sequences were assigned GO terms. These terms belong to 57 functional groups, which were distributed under three main categories: molecular function, biological process, and cellular component (Fig 3). In the molecular function category, “binding,” “catalytic,” and “transporter” were the most mapped terms. In the biological process category, “biological regulation,” “cellular process,” “metabolic process,” “response to stimulus,” and “single-organism process” were the most mapped terms. In the cellular component category, “cell,” “cell part,” “organelle,” “membrane,” and “organelle part” were the mainly mapped terms. Furthermore, a few unigenes were mapped to terms “cell killing,” “extracellular matrix component,” “other organism,” “other organism part,” “nutrient reservoir activity,” “protein tag,” “translation regulator,” and “metallochaperone activity.”

**Fig 4:**
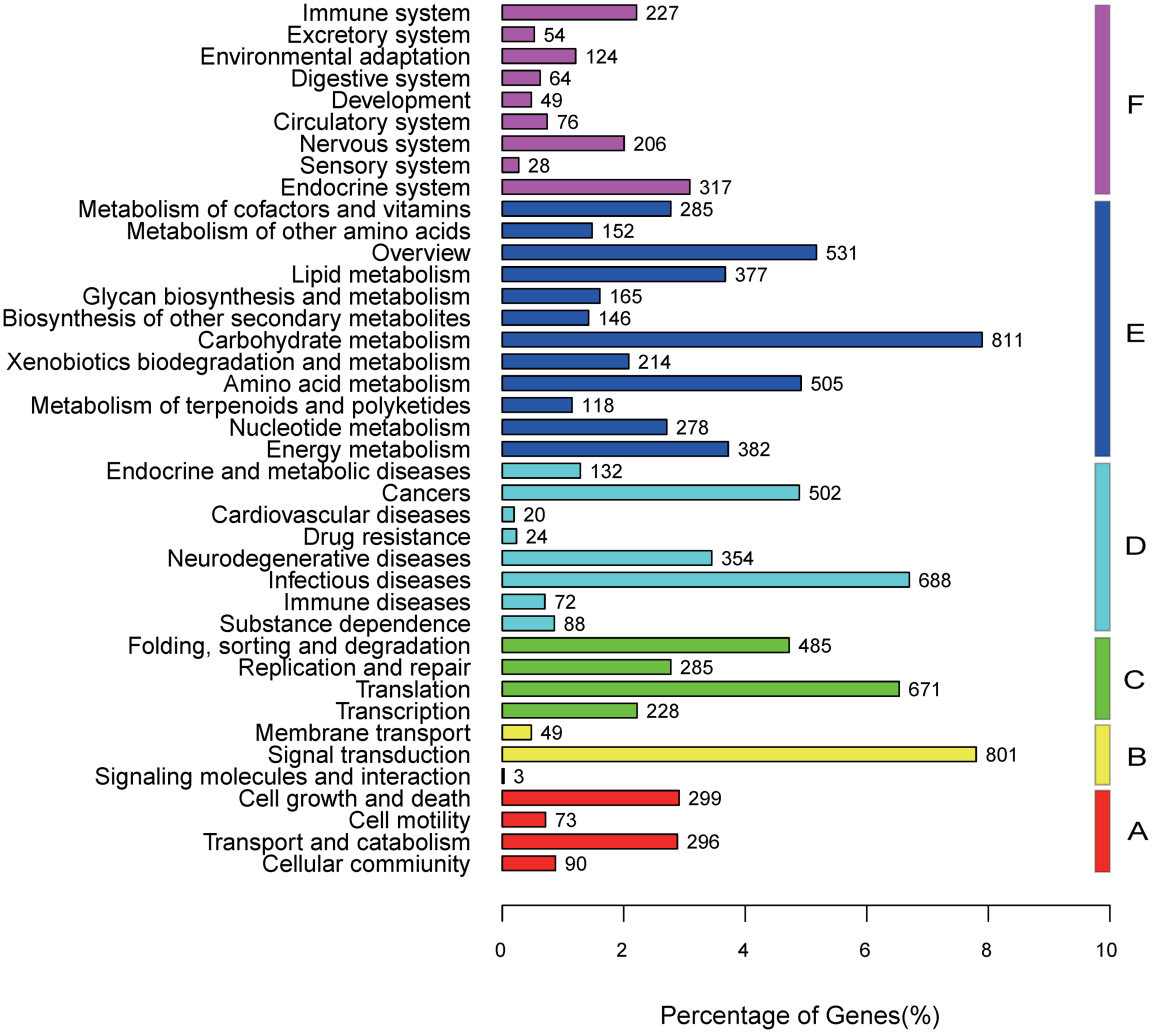
KEGG classifications of all unique sequences. A: Cellular Processes; B: Environmental Information Processing; C: Genetic Information Processing; D: Human Diseases; E: Metabolism and F: Organismal Systems.

For the KEGG pathways, 6085 unique sequences were assigned to the pathways (Fig 4). The top 10 most mapped pathways were “Ribosome” (322 sequences), “Carbon metabolism” (213 sequences), “Biosynthesis of amino acids” (196 sequences), “Purine metabolism” (157 sequences), “Spliceosome” (146 sequences), “Protein processing in endoplasmic reticulum” (146 sequences), “Oxidative phosphorylation” (135 sequences), “RNA transport” (130 sequences), “Huntington’s disease” (123 sequences), and “Pyrimidine metabolism” (121 sequences). In particular, KEGG analysis showed that 21 unigene sequences were involved in flavonoid biosynthesis (S5 Table). Compared with those described for the *E*. *sagittatum* dataset, 54% of unigene sequences in our dataset were mapped to GO terms, whereas only 29.2% transcripts were mapped to GO terms for the *E*. *sagittatum* dataset.

### 3.4. Determination of gene abundance and identification of differentially expressed genes

**Fig 5:**
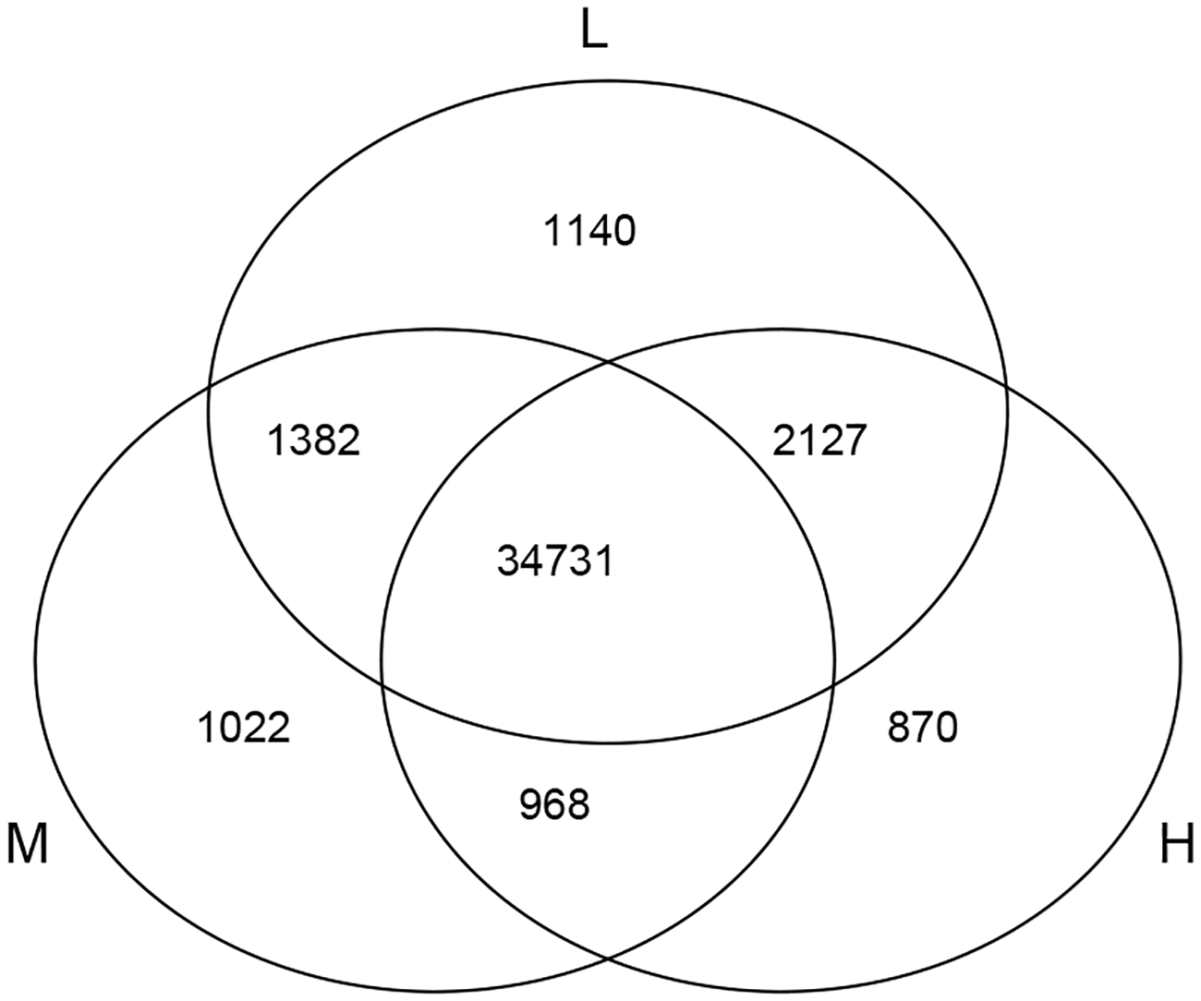
Venn diagram indicating the numbers of genes expressed under different light. The number of genes annotated is listed in each diagram component.

**Fig 6:**
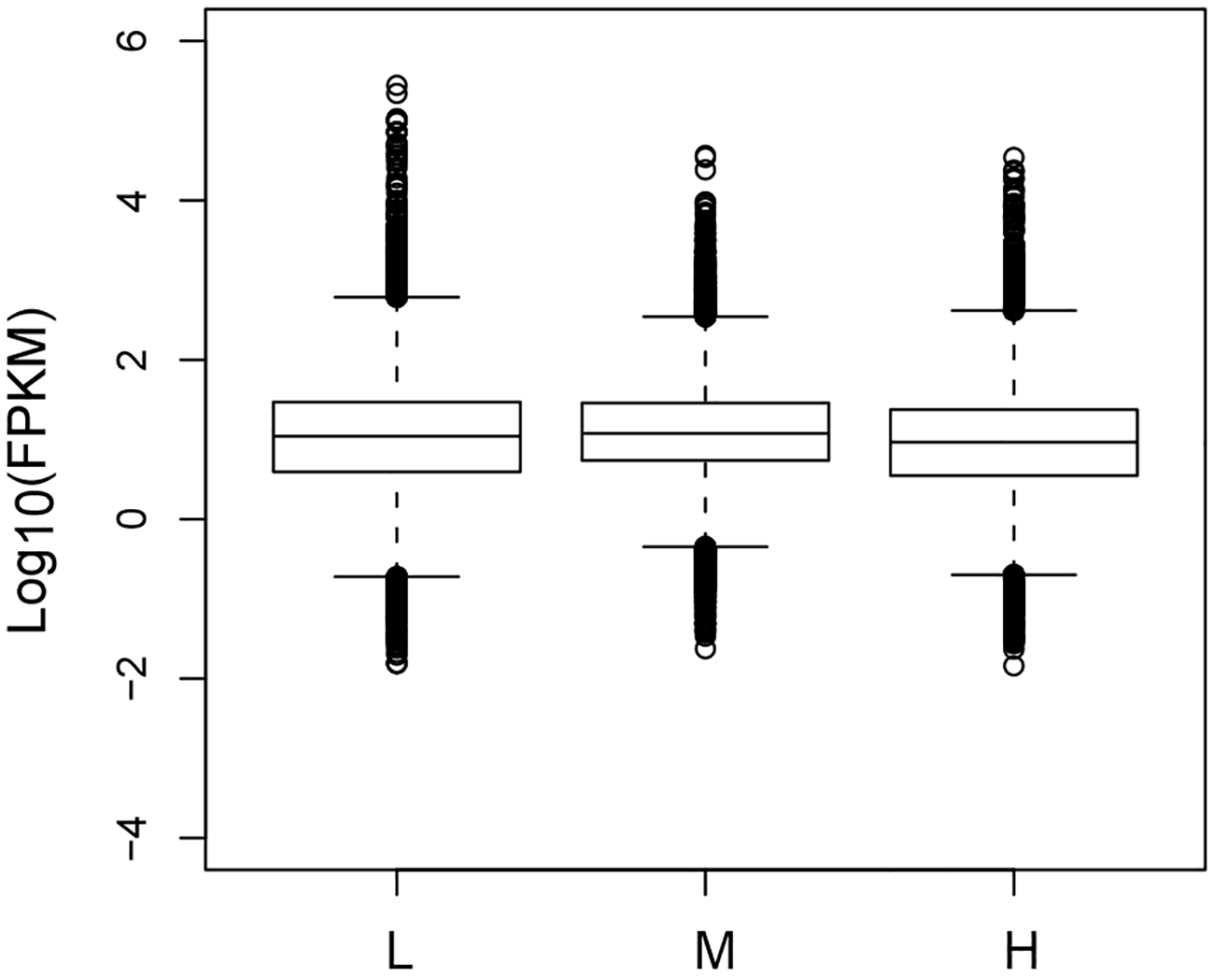
Distribution of gene expression levels across three treatment groups.

**Fig 7:**
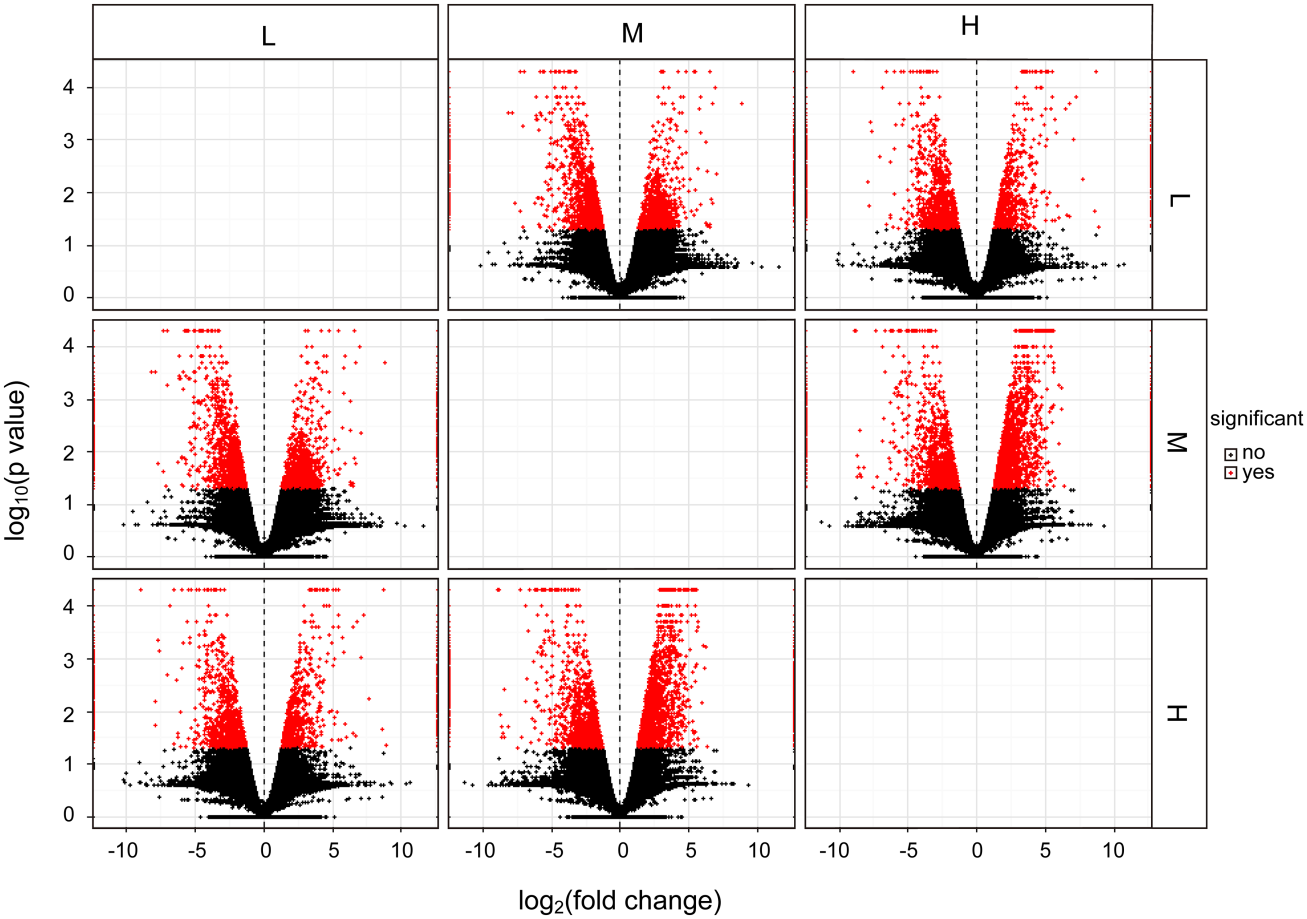
Volcano plot showing the distribution of log10(p value) vs. log2(Fold Change). The horizontal axis represents the log2(Fold Change) between the two samples indicated on the top or on the right of the figure, while the vertical axis represents the log10(p value) for the differential expressions between the two samples. Each point represent a gene, red points indicate p value ≤ 0.05, while blue points indicate that p value > 0.05.

The abundance of unigene sequences was quantified using the program cuffdiff and represented by FPKM [28]. The pearson correlation coefficients of gene expression levels between biological replicates are 0.76, 0.79 and 0.81 for the three treatment groups respectively. A total of 39,380, 38,103, 38,696 expressed genes were identified in groups L, M, and H, respectively. As shown in Fig 5, a total of 34731 genes were expressed in all three treatments. Among them, there were 1140, 1022 and 870 genes expressed only in the L, M and H treatment, respectively. Boxplots showing the abundance distribution are shown in Fig 6 and S6 Table. It appears that the overall deistrubtion of gene expression levels are similar for the three treatment groups. Similarly, the differentially expressed genes (DEGs) were also identified using cuffdiff. The volcano plots showing the distribution of fold changes and p values are shown in Fig 7. A total of 4008 DEGs were identified between groups L and M, of which 1928 were upregulated and 2080 were downregulated. By contrast, 5260 DEGs were found between groups M and H, of which 2468 were upregulated and 2792 were downregulated. Lastly, 3591 DEGs were detected between groups L and H, of which 1289 were upregulated and 2302 were downregulated. Details for these DEGs can be found in Fig 7 and S7 Table. These DEGs are potentially involved in the light-induced accumulation of flavonoids.

### 3.5. Functional enrichment analysis of DEGs

To further narrow down the genes that are involved in light-induced flavonoid biosynthesis, the DEGs were first mapped to GO terms. The distribution of mapped GO classifications is shown in S2 Fig. The details for the mapping can be found in S8 Table. The most mapped terms of DEGs for the categories of biological process and cellular component were “defense response” and “integral component of membrane.” In the category of molecular function, the most mapped term for DEGs between groups L and M was “Metal ion binding.” By contrast, the most mapped term for DEGs between groups M and H was “ATP binding.” Furthermore, the most mapped term between groups L and H was “protein serine/threonine kinase activity.”

In parallel, the DEGs were mapped to KEGG pathways. The most enriched pathways between groups L and M included a two-component system in environmental information processing and signal transduction (22 DEGs), phenylpropanoid biosynthesis (14 DEGs), and glyoxylate and dicarboxylate metabolism in carbohydrate metabolism (14 DEGs) (S9 Table). By contrast, the most enriched pathways between groups M and H included starch and sucrose metabolism (39 DEGs), amino sugar and nucleotide sugar metabolism (27 DEGs), and phenylpropanoid biosynthesis (20 DEGs) (S9 Table). The results confirmed that light-induced flavonoid accumulation is mediated through the increased expression levels of genes involved in the biosynthesis of phenolic acids and flavonoids. Furthermore, a dose-response relationship exists between light intensity and gene expression levels.

### 3.6. Enzyme genes involved in the biosynthesis of active compounds in *E*. *pseudowushanense*

The flavonoid pathway can be divided into three pathways leading to the production of anthocyantin, proanthocyanin, and flavonol, respectively [29]. Basing on the structural characteristics of the compounds, we proposed a pathway for the biosynthesis of flavonoids in *E*. *pseudowushanense* (S3 Fig). In this proposed pathway, L-phenylalanine is first converted to trans-cinnamic acid by phenylalanine ammonia-lyase (PAL, EC: 4.3.1.24) and subsequently to p-coumaric acid by trans-cinnamate 4-hydroxylase (C4H, EC: 1.14.13.11). p-Coumaric acid can be converted into p-coumaroyl-CoA by 4-coumarate-CoA ligase (4CL, EC: 6.2.1.12) and then catalyzed by chalcone synthase (CHS, EC: 2.3.1.74), chalcone isomerase (CHI, EC: 5.5.1.6), and flavanone 3-hydroxylase (EC: 1.14.11.9). As the product of these steps, dihydrokaempferol can be further converted into kaempferol by flavonol synthase (FLS, EC: 1.14.11.23), which is then converted to prenyl-flavonoids such as icariin by UGT, OMT, and some unknown methoxy transferase and isopentenyl transferase. Alternatively, kaempferol can be either hydroxylated by flavonoid 3′ hydroxylase (EC: 1.14.13.21) to produce dihydroquercetin. Furthermore, kaempferol can be converted successively by dihydroflavonol 4-reductase (DFR, EC: 1.1.1.219) and leucoanthocyanidin dioxygenase (EC: 1.14.11.19) to generate anthocyanin.

In our study, 21 unique sequences that encode 14 enzyme families involved in the flavonoid biosynthetic pathways were identified (Table 2). The short and full gene names were shown. A prefix “epps” was added to the short gene name to indicated that it is derived from *E*. *pseudowushanense*. Multiple sequence alignments of the identified proteins and their homologous sequences were conducted to determine if the full-length sequences have been obtained. Furthermore, phylogenetic trees were constructed to examine the relationship of the following proteins: PAL (S4 Fig), 4CL (S5 Fig), caffeoyl-CoA O-methyltransferase (S6 Fig), CHS (S7 Fig), CHI (S8 Fig), leucoanthocyanidin dioxygenase (S9 Fig), FLS (S10 Fig), flavonoid 3′-monooxygenase (S11 Fig), anthocyanidin reductase (S12 Fig), naringenin 3-dioxygenase (S13 Fig), bifunctional dihydroflavonol 4-reductase/flavanone 4-reductase (S14 Fig), trans-cinnamate 4-monooxygenase (S15 Fig), shikimate O-hydroxycinnamoyltransferase (S16 Fig), and coumaroylquinate(coumaroylshikimate) 3'-monooxygenase (S17 Fig). As shown in the Figures, all *E*. *pseudowushanense* genes are highly similar to their homolgous sequences. The phylogenetic relationship among these genes are consisitent with the those of the species. Based on the length of their homologs, it is likely that all these sequences containing the full-length codoing sequences.

**Table 2:** Putative enzymes involved in the production of the active compounds in the leaf samples of *E*. *pseudowushanense*.

Unigene_id	Short Gene name	KEGG Pathway ID (KO)	Full Gene Name	EC number
TR2108|c0_g1	eppsPAL1	K10775	phenylalanine ammonia-lyase	E4.3.1.24
TR575|c1_g1	eppsPAL2	K10775	phenylalanine ammonia-lyase	E4.3.1.24
TR10614|c0_g1	epps4CL1	K01904	4-coumarate—CoA ligase	EC:6.2.1.12
TR1945|c0_g1	epps4CL2	K01904	4-coumarate—CoA ligase	EC:6.2.1.12
TR9038|c0_g1	epps4CL3	K01904	4-coumarate—CoA ligase	EC:6.2.1.12
TR11481|c3_g2	eppsF3'H	K05280	flavonoid 3'-monooxygenase	EC:1.14.13.21
TR11916|c0_g1	eppsCHS1	K00660	chalcone synthase	EC:2.3.1.74
TR18393|c0_g1	eppsCHS2	K00660	chalcone synthase	EC:2.3.1.74
TR9672|c0_g1	eppsCHS3	K00660	chalcone synthase	EC:2.3.1.74
TR17306|c0_g1	eppsCOMT1	K00588	caffeoyl-CoA O-methyltransferase	EC:2.1.1.104
TR1231|c0_g1	eppsCOMT2	K00588	caffeoyl-CoA O-methyltransferase	EC:2.1.1.104
TR10281|c0_g1	eppsCOMT3	K00588	caffeoyl-CoA O-methyltransferase	EC:2.1.1.104
TR1877|c0_g1	eppsC3'H	K09754	coumaroylquinate(coumaroylshikimate) 3'-monooxygenase	EC:1.14.13.36
TR19370|c0_g1	eppsCHI	K01859	chalcone isomerase	EC:5.5.1.6
TR19880|c0_g1	eppsDFR	K13082	bifunctional dihydroflavonol 4-reductase/flavanone 4-reductase	EC:1.1.1.219 1.1.1.234
TR3386|c0_g1	eppsANR	K08695	anthocyanidin reductase	EC:1.3.1.77
TR4169|c0_g1	eppsF3H	K00475	naringenin 3-dioxygenase	EC:1.14.11.9
TR465|c1_g1	eppsHCT	K13065	shikimate O-hydroxycinnamoyltransferase	EC:2.3.1.133
TR6321|c0_g1	eppsFLS	K05278	flavonol synthase	EC:1.14.11.23
TR8942|c0_g1	eppsC4H	K00487	trans-cinnamate 4-monooxygenase	EC:1.14.13.11
TR9962|c0_g1	eppsLDOX	K05277	leucoanthocyanidin dioxygenase	EC:1.14.11.19

### 3.7. Correlation between the expression profiles of biosynthetic genes and flavonoid contents

To determine which flavonoid biosyntheis genes were most strongly induced by light, we examined the differential expression of these genes as well as the correlation of the expression profiles of these genes and those of the flavonoid contents across the three treatment conditions (Table 3). The expression level of FLS was upregulated between groups L and M. The expression levels of CHS and FLS were upregulated between groups L and H. Lastly, the expression levels of C4H, CHI, and FLS were upregulated, whereas that of caffeoyl-CoA O-methyltransferase (COMTEC: 2.1.1.104) was downregulated between groups M and H. The expression profiles of four of the twenty-one unigenes were found to be highly correatled with those of the flavonid contents, with pearson correlation coefficents ≥ 0.9. In summar, the genes FLS, CHS, C4H and CHI seemed to be most strongly associated with the light-induced flavonoid accumulation.

**Table 3:** Correlation analysis between flavonol contents and expression profiles of the related genes.

Unigene_id	Gene name	Expression levels (FPKM)			Correlation Coefficient
		L	M	H	
TR10614|c0_g1	epps4CL1	158.079	168.0136	196.307	0.999
TR9962|c0_g1	eppsLDOX	196.149	244.779	490.129	0.992
TR17306|c0_g1	eppsCOMT1	1.45126	5.45209	11.2109	0.991
TR2108|c0_g1	eppsPAL1	196.992	218.676	384.105	0.984
TR4169|c0_g1	eppsF3H	254.675	378.125	1347.9	0.984
TR6321|c0_g1*	eppsFLS	101.355	170.6277	815.999	0.981
TR11916|c0_g1*	eppsCHS1	197.028	274.591	1374.08	0.975
TR19880|c0_g1	eppsDFR	152.986	271.555	352.847	0.941
TR8942|c0_g1*	eppsC4H	229.376	188.182	732.647	0.938
TR11481|c3_g2	eppsF3'H	70.708	124.069	154.145	0.922
TR9672|c0_g1	eppsCHS3	297.071	152.765	1259.05	0.919
TR19370|c0_g1*	eppsCHI	242.674	168.983	686.668	0.914
TR1231|c0_g1	eppsCOMT2	47.8312	43.5506	73.3397	0.914
TR1945|c0_g1	epps4CL2	59.4564	50.2029	85.7176	0.858
TR9038|c0_g1	epps4CL3	27.5068	45.1075	48.3871	0.816
TR18393|c0_g1	eppsCHS2	890.587	2830.32	1995.24	0.357
TR3386|c0_g1	eppsANR	374.384	786.82	588.589	0.303
TR575|c1_g1	eppsPAL2	170.524	290.408	231.094	0.287
TR1877|c0_g1	eppsC3'H	81.7185	105.585	44.2763	-0.777
TR10281|c0_g1	eppsCOMT3	2074.99	1290.9	253.881	-0.987
TR465|c1_g1	eppsHCT	102.536	82.3289	49.0085	-0.995
**Total flavonoid contents**^a^ **(mg/g)**		19.886	53.798	137.100	

^a^Total flavonoid content is the sum of Epimedin A, Epimedin B, Epimedin C and Icariin;

*significantly, differentially, up-regulated genes.

**Fig 8:**
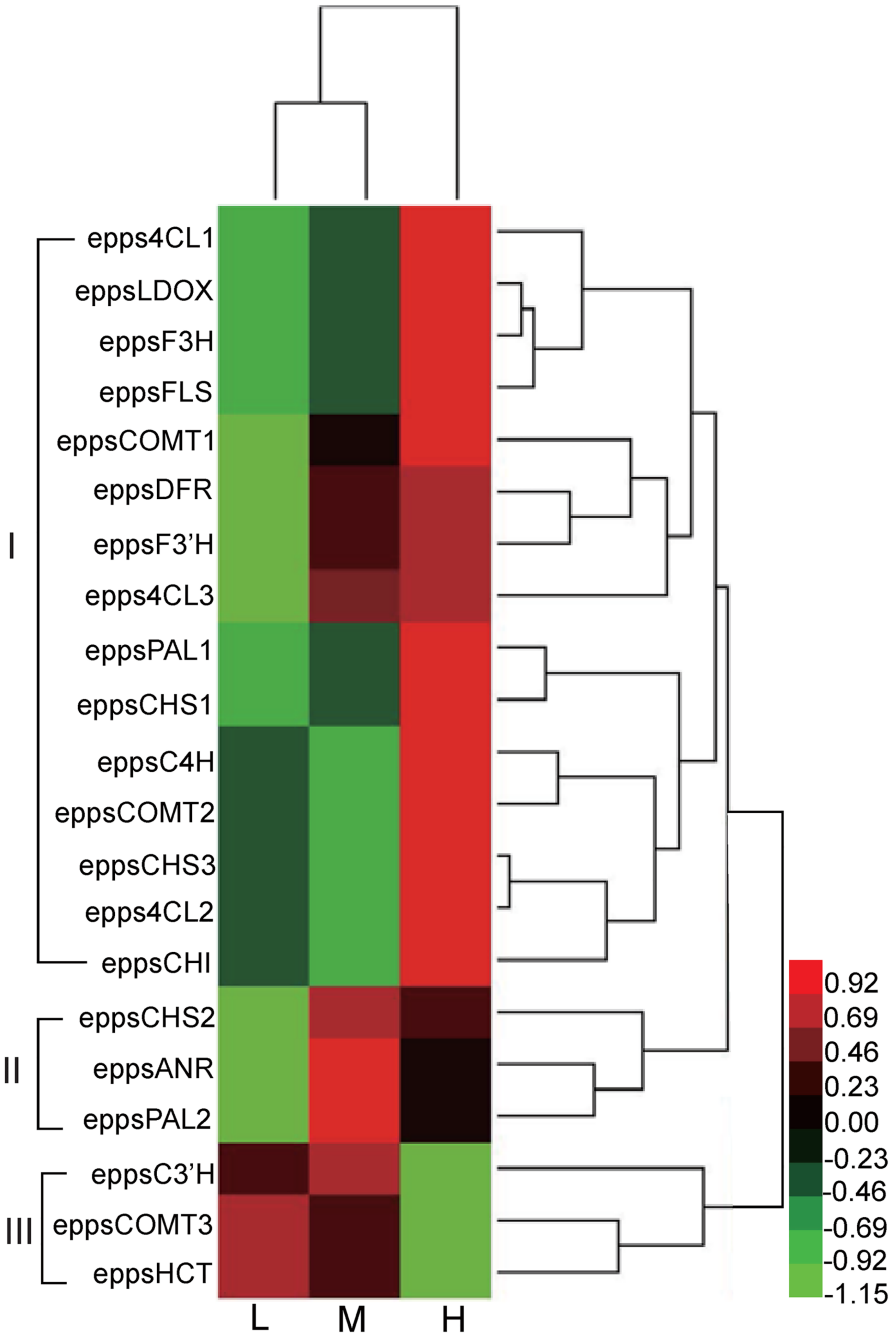
Hierarchical clustering of 21 flavonoid biosynthesis genes. Red, green and black colors in the heat-map represent relative gene expression levels. The scale bar denotes the log (FPKM+1)/(mean expression levels across the three treatment groups). The green color indicates down-regulated expression compared with the mean expression levels, and the red color indicates up-regulated expression compared to the mean. Three clusters (I, II and III) were indicated with brackets.

To study the co-expression patterns of these genes, we performed hierarchical clustering of the expression profiles of these 21 flavonoid biosynthesis genes (Fig 8). Three main clusters were readily discernable, the first cluster contained 15 genes that showed the highest expression levels in the “H” group. The second cluster contain 3 genes that were expressed at the highest levels in the “M” group. The remaining three genes belonged to the third cluster and had the highest expression levels in the “L” group. Genes in the cluster I have expression profiles that were better corrleated with the flavonoid contents comparing to those of the cluster II and III. It should be pointed out that the four genes FLS, CHS, C4H and CHI all belonged to cluster I.

### 3.8. RT-qPCR validation

**Fig 9:**
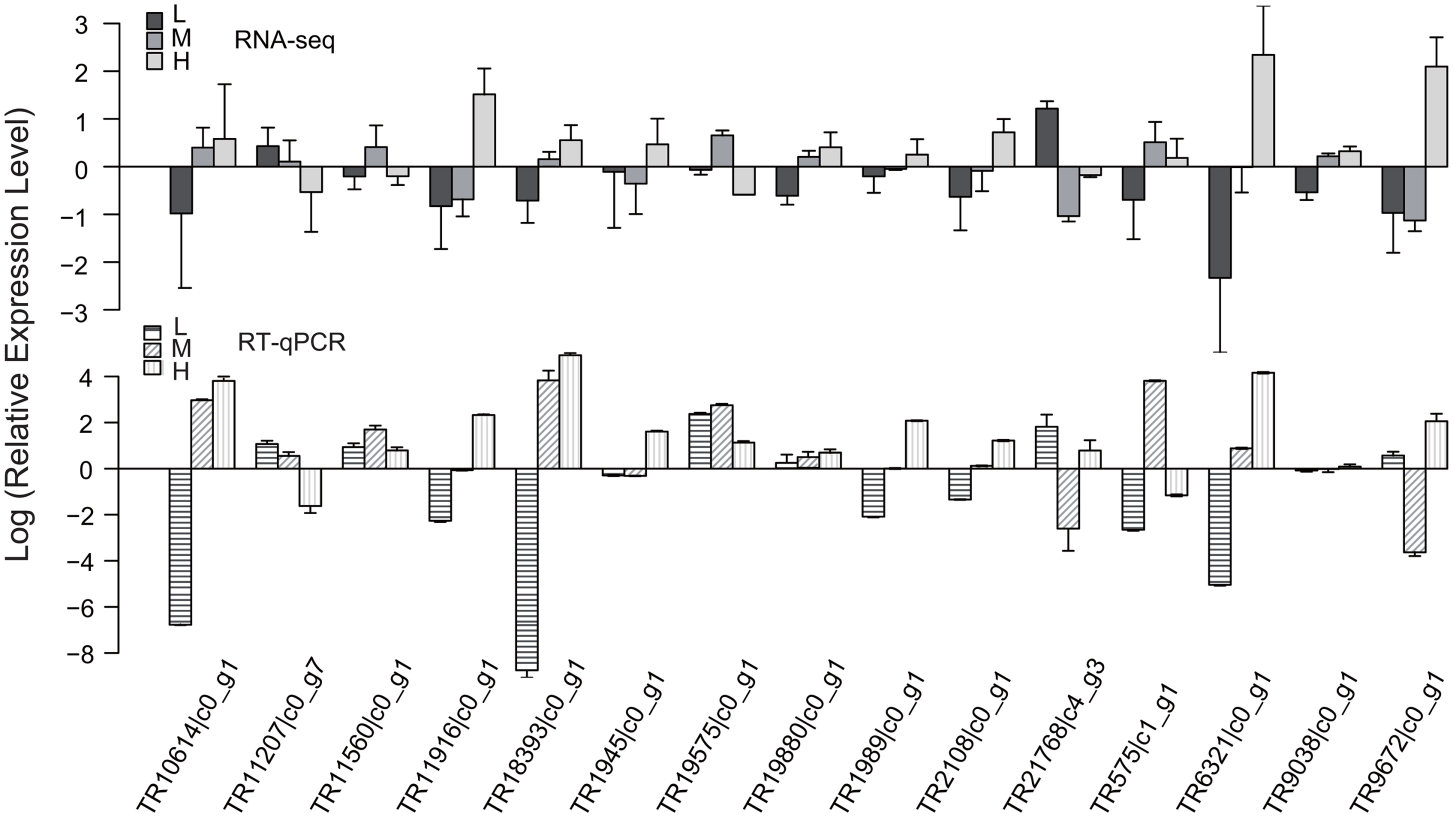
Comparison of the expression patterns of fifteen genes obtained from RNA-seq experiments with those obtained from RT-qPCR experiments. Relative expression values, normalized to actin, were shown as 2^−ΔΔCt^ relative to mean of the L, M and H treatments. Error bars represent the SD of three biological replicas with three technical replicas each.

To validate the RNA-seq data, 15 genes were selected and subjected to RT-qPCR analysis. These genes include the 2 genes that are highly correlated and differentially expressed, two genes (TR2108|c0_g1, TR575|c1_g1) belonged to the PAL family, one gene (TR6321|c0_g1) belonged to the FLS family, three genes (TR1945|c0_g1, TR10614|c0_g1, TR9038|c0_g1) belong to the 4CL family, three genes (TR11916|c0_g1, TR9672|c0_g1, TR18393|c0_g1) belonged to the CHS family, one gene (TR19880|c0_g1) belonged to the DFR family and five genes (TR11207|c0_g7, TR11560|c0_g1, TR1989|c0_g1, TR19575|c0_g1, TR21768|c4_g3) belongd to the UGT family. Except for TR575|c1_g1, TR9672|c0_g1, TR19880|c0_g1 and TR9038|c0_g1, the expression profiles determined by RNA-Seq experiments correlated well with those obtained from RT-qPCR experiments for 11 out of 15 (73.3%) genes with pearson correlation coefficients (r) > 0.9 (S10 Table). And 15 of 16 pairs of expression profiles were found to be significantly correlated (p < 0.05). This finding suggests that the results of our RNA-seq experiments are reliable (Fig 9).

**Fig 10:**
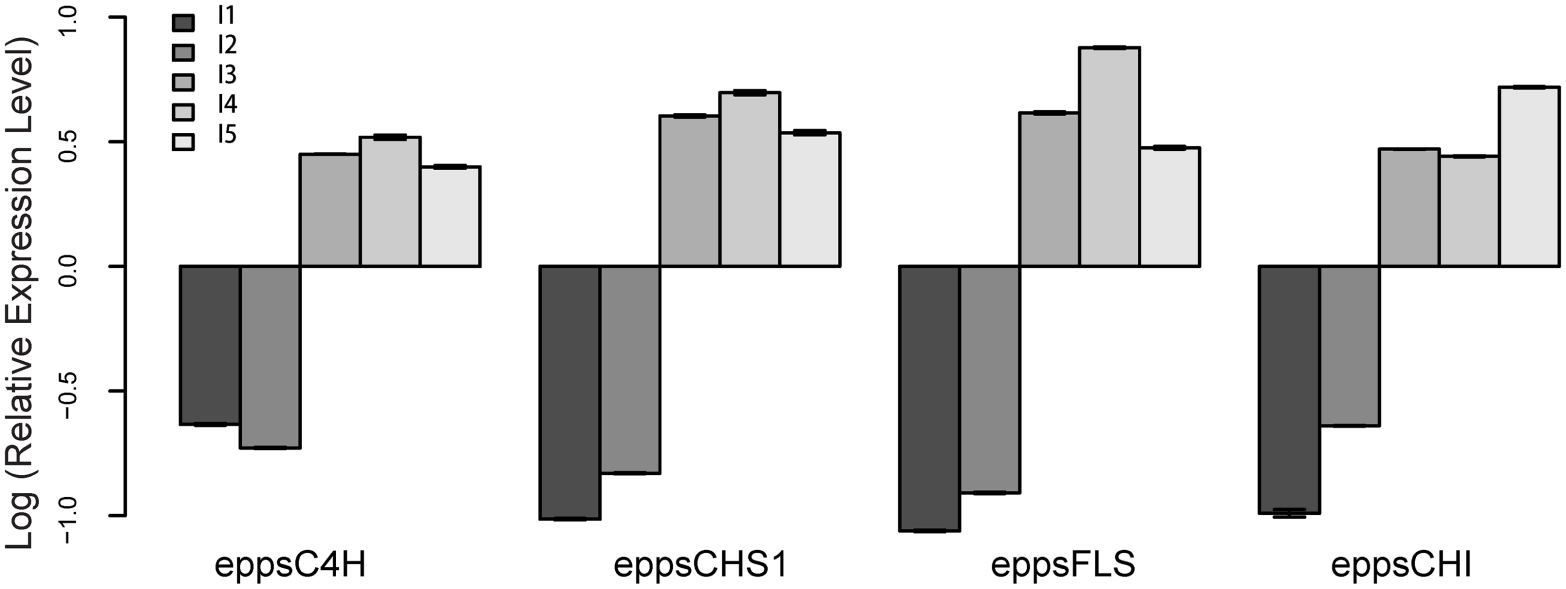
RT-qPCR analysis of differential expression genes involved in flavonoids biosynthesis in *E*. *pseudowushanense* at different light intensities. Relative expression values, normalized to actin, were shown as 2^−ΔΔCt^ relative to mean of the five light treatments. Error bars represent the SD of three biological replicas with three technical replicas each.

To see if any correlations existed between flavonoid content and expression patterns of the flavonoids biosynthesis genes, we analyzed transcript abundance of four related genes (*C4H*, *CHS1*, *FLS*, and *CHI*) by RT-qPCR from 5 different light intensity described in Fig 1. The relative expression level of the four genes showed similar changes from I1 to I5 light intensity. The expression of four related genes under I1 and I2 are lower than I3 to I5. Interestingly the changes of *CHS1* and *FLS* showed similarly changes to epimedin B, epimedin C and icariin. The I4 treatment showed highest expression level at *C4H*, *CHS1* and *FLS* while the I5 treatment showed highest expression level at *CHI* gene (Fig 10**)**.

### 3.9. Transcriptional factors involved in the light-induced flavonoid accumulation in *E*. *pseudowushanense*

To understand how the expression of genes involved in flavonoid biosynthesis was regulated in response to light, we first identified all unique sequences encoding the transcription factors in our RNA-seq dataset by comparing to sequences in the plant transcription factor database using BLAST with an E value cutoff of 1e^−5^ (S11 and S12 Tables). We identified 4621 unigene sequences that likely encode transcription factors. The lengths of unigene sequences representing these transcription factors varied from 224 to 13,144 bp, with an average length of 1241.5 bp and an N50 value of 1863 bp. The length distribution of these putative transcriptional factor genes is shown in S18 Fig. In terms of types, the identified transcription factors were distributed in 59 families, such as C3H, bHLH, FAR1, WRKY, NAC, MYB-related, and so on (S19 Fig). The differentially expressed transcription factors after light treatment mainly belong to the families FAR1, WRKY, bHLH, and MYB-related families (S13 Table).

To select further the transcription factors that are involved in the light-induced flavonoid accumulation, we first collected the sequences of all transcription factors from *Arabidopsis thaliana*, *Oryza sativa*, *V*. *vinifera*, and *E*. *sagittatum*, based on (1) similarity to known transcription factors involved in flavonoid biosynthesis; (2) p value for differential gene expression in any contrast group; and (3) correlation between gene expression profiles and flavonoid contents. Transcription factors including 31 FAR1, 17 MYB, 12 bHLH, and 5 WRKY are likely involved in light-induced flavonoid accumulation (S11 Table).

### 3.10. Light signalingl factors involved in the light-induced flavonoid accumulation in *E*. *pseudowushanense*

To select the light signaling factors that are most likely involved in the light-induced flavonoid accumulation, we collected them, based on (1) similarity to known light signal factors involved in flavonoid biosynthesis; (2) p value for differential gene expression in any contrast group; and (3) correlation between gene expression profiles and flavonoid contents(>|0.9|). Light signal factors including 3 COP1, 1 pif, 1 HY5, 1 SPA, 1 DET, 3 phy and 3 cry are likely involved in light-induced flavonoid accumulation (S14 Table).

## 4. Discussion

### 4.1 Enzymatic genes involved in flavonoid biosynthesis

Previous studies demonstrated that light treatment of grape and kale could influence gene expression, leading to the accumulation of specific flavonol glycosides [28,30]. Further studies in grape berries reported that flavonol levels are sensitive to changes in light conditions; flavonols accumulate with increased expression of FLS [31–33]. These studies suggest that the expression levels of genes involved in flavonoid biosynthesis are regulated by light. In the present study, we found that C4H, CHS, CHI, and FLS were all upregulated under the different light treatments, partially explaining the light-induced flavonol accumulation in *E*. *pseudowushanense*.

### 4.2 Transcription factors involved in light-induced flavonoid biosynthesis

Transcription factors regulate the secondary metabolite biosynthesis and accumulation of flavonoids. Several families of transcription factors play roles in the production of flavonol compounds. Qiu et al. [34] identified a WRKY protein (OsWRKY13) as a transcriptional regulator of flavonoid biosynthesis in *O*. *sativa*, which could induce the expression of CHS. WRKY transcription factors are defined by the presence of the DNA-binding domain WRKY. The identified WRKY genes are significant regulators involved in plant developmental processes and responses to biotic and abiotic signals [35]. The inducible expression patterns of WRKY genes suggest that they are involved in the regulation of plant secondary metabolis [36].

As for flavonol biosynthesis, several specific regulators belonging to the MYB transcriptional factor family have been identified in model species. MYB proteins are characterized by the presence of one or many MYB repeat (R) DNA-binding domains. In *A*. *thaliana*, AtMYB12 activates the expression of AtFLS and AtCHS [37]. In grape, VvMYBF1, orthologous to AtMYB12, markedly upregulated the expression levels of VvFLS and VvCHI [38]. In *E*. *sagittatum*, some MYB members have been isolated and characterized, among which EsMYBF is homologous to AtMYB12 that is related to flavonol synthesis [30,39]. In grape, light induces the expression of an array of MYB transcription factors, such as VvMYBF1 and VvMYB12, which are positive regulators of the general flavonoid biosynthesis pathway as well as those specifically responsible for flavonol biosynthesis [31,40]. MYB transcription factors can directly and specifically interact with MYB recognition element (MRE). MRE is part of the light regulatory unit, which also contains bZIP recognition element (ACE). MREs can be found in the promoter regions of light-induced structural flavonoid genes, such as CHS and FLS in Arabidopsis and grapevine [41, 42].

The expression levels of these MYB are also regulated by other transcription factors, such as Elongated Hypocotyl 5 (HY5). HY5 is a bZIP transcription factor that can promote photo-morphogenesis [43] by recognizing ACE. In particular, HY5 has been linked to the activation of MYB and key structural genes (CHS and FLS) of the flavonoid pathway as well as the accumulation of flavonoids in response to light in Arabidopsis [44–47].

Located further upstream of the regulatory pathway, HY5 is a direct target of RING-finger-type ubiquitin E3 ligase Constitutive Photo-morphogenic 1 (COP1). COP1 acts as a negative regulator of light signaling directly downstream of the photoreceptors and controls different light-regulated plant development processes by adjusting its subcellular localization. In the presence of light, the interaction of the COP1/Suppressor of PhyA (SPA) complex with activated photoreceptors inhibits COP1/SPA function through the dissociation of COP1 from the complex and exportation from the nucleus. The downregulation of COP1 in the nucleus allows nuclear-localized transcription factors, such as HY5, to accumulate and induce the expression of genes responsible for flavonoid biosynthesis [48].

Aside from the transcription factors described above, other important classes of transcriptional factors that might be involved in flavonoid biosynthesis include the Far-red impaired Response 1 (FAR1) and Far-Red Elongated Hypocotyl 3 (FHY3) families [49]. FAR1 and FHY3 participate in diverse developmental and physiological processes and are essential for PhyA signaling in A. thaliana [50–51]. HY5 physically interacts with FHY3/FAR1 through their respective DNA binding domains in *A*. *thaliana* [52].

### 4.3 Other pathways related to light-induced flavonoid accumulation

Enrichment analysis showed that DEGs are significantly enriched for those involved in the two-component regulatory system, suggesting that this pathway might be involved in light-induced flavonoid accumulation. A two-component regulatory system is a basic stimulus-response coupling mechanism to allow organisms to sense and respond to changes in different environmental conditions [53]. Two-component systems typically consist of a membrane-bound histidine kinase that senses a specific environmental stimulus and a corresponding response regulator that mediates the cellular response, mostly through the differential expression of target genes [54]. Two-component regulatory systems are also commonly found in plants. How this system is involved in light-induced flavonoid accumulation in *E*. *pseudowushanense* represents an interesting research question in the future.

### 4.4 Model proposed

**Fig 11:**
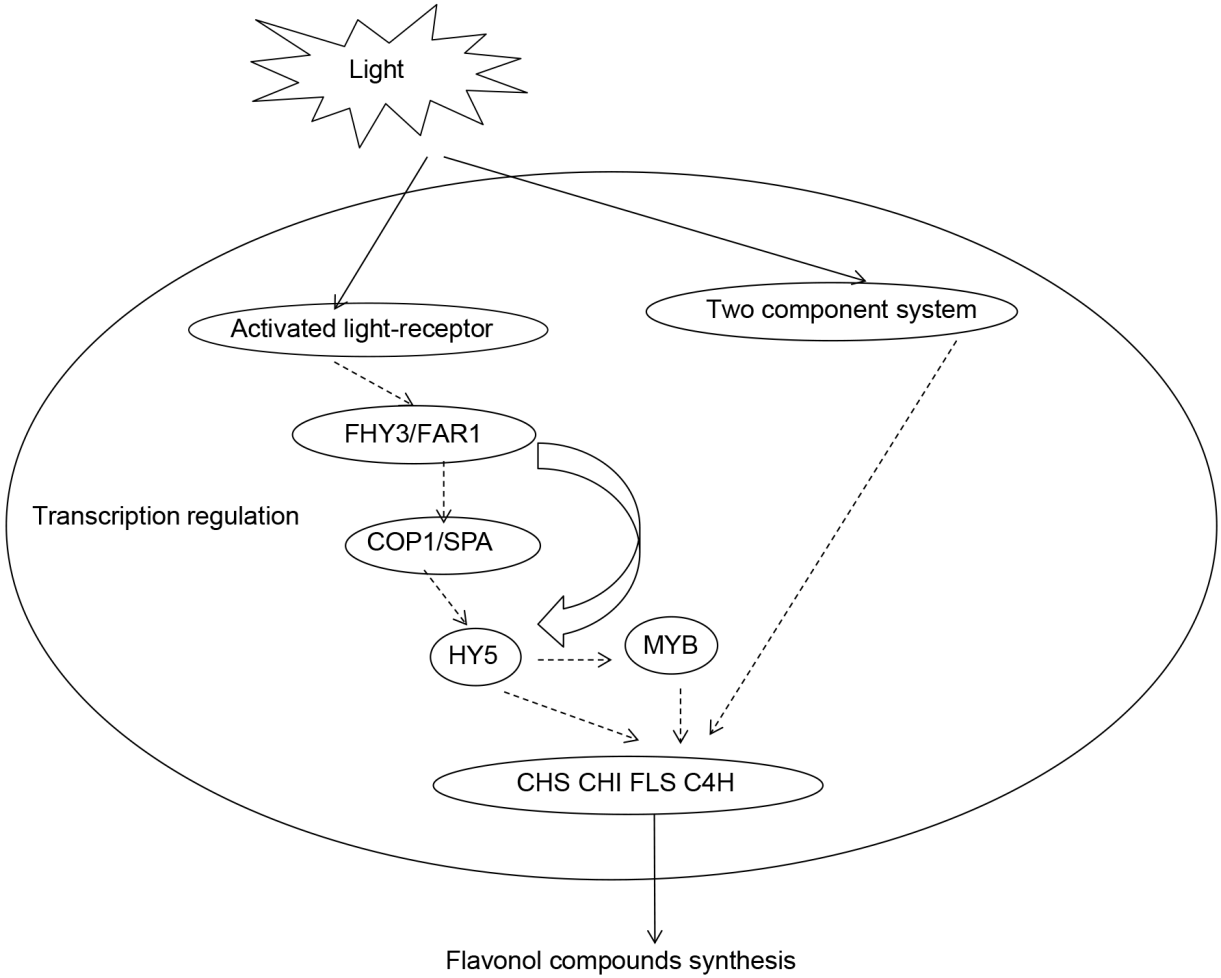
A putative model for the light-induced flavonoids biosynthesis in *E*. *pseudowushanense*.

To date, the mechanism by which light induces the biosynthesis of specific flavonoids in *Epimedium* is unknown. However, analysis of our transcriptome data implies that the mechanism of flavonoid accumulation in *E*. *pseudowushanense* is rather complex. Basing on previous studies, we proposed a model explaining light-induced flavonoid accumulation (Fig 11). In this model, light signals are received either by photoreceptors such as phytochrome or the two-component regulatory system through downstream signaling pathways, leading to the upregulation of genes involved in flavonoid biosynthesis and ultimately resulting in the accumulation of these compounds. This model will serve as a central hypothesis for the light-induced flavonoid biosynthesis that will be tested in the future.

## 5. Conclusions

This study represents the first comprehensive investigation of the genetic makeup responsible for the flavonol biosynthesis in *E*. *pseudowushanense*. Firstly, we find I4 light intensity is optimal for flavonoid ingredient accumulation. Then, we identified 43,657 unigene sequences in *E*. *pseudowushanense* from samples treated with light at three intensity levels by using RNA-seq technology. We determined the full-length sequences of 21 enzymatic genes involved in the flavonol biosynthesis. Among them, the FLS, CHS1 genes were strongly associated with light-induced flavonoid accumulation. We also found 65 transcription factors, including 31 FAR1, 17 MYB-related, 12 bHLH, and 5 WRKY, which might participate in light-induced flavonoid accumulation. A model was proposed to explain the underlying molecular mechanism. This work provides valuable resources for further studies on flavonoid production in *Epimedium*. These information can help us to know why the flavonoid content changed under different light conditions. Besides in vitor experiments could be conduct to examine the fouction of FLS and CHS1 under diferent light intensities.

## Supporting information

S1 Table Primers used in RT-qPCR. (XLSX)

S2 Table Length distribution of transcripts. (XLSX)

S3 Table Reads mapping results. (XLSX)

S4 Table Mapping of unigenes by BLAST. (XLSX)

S5 Table Summary of KEGG annotation results. (XLSX)

S6 Table Abundance distribution by RPKM. (XLSX)

S7 Table List of differentially expressed genes (DEGs). (XLSX)

S8 Table Enrichment analysis by GO. (XLSX)

S9 Table Enrichment analysis by KEGG. (XLSX)

S10 Table Correlation analysis between RNA-seq and RT-qPCR. (XLSX)

S11 Table Putative transcription factors. (XLSX)

S12 Table Transcription factor families. (XLSX)

S13 Table Differentially expressed TFs. (XLSX)

S14 Table Correlation analysis between flavonol contents and expression profiles of the phytochrome related genes. (XLSX)

S1 Fig Species taxonomy structure of Nr annotation. (DOCX)

S2 Fig GO classifications of DEGs between different light conditions. Annotated unique sequences were classified into ‘Biological process’, ‘Cellular component’ and ‘Molecular function’. Panels (A), (B) and (C) are for different groups.
(DOCX)

S3 Fig Putative flavonoid biosynthesis pathway of *E*. *pseudowushanense*. (DOCX)

S4 Fig Sequence alignment of phenylalanine ammonia-lyase (PAL) proteins from *E*. *pseudowushanense* and various other plants, and phylogenetic relationships of phenylalanine ammonia-lyase (PAL) proteins from *E*. *pseudowushanense* and various other plants. (DOCX)

S5 Fig Sequence alignment of 4-coumarate-CoA ligase (4CL) proteins from *E*. *pseudowushanense* and various other plants, and phylogenetic relationships of 4-coumarate-CoA ligase (4CL) proteins from *E*. *pseudowushanense* and various other plants. (DOCX)

S6 Fig Sequence alignment of caffeoyl-CoA O-methyltransferase proteins from *E*. *pseudowushanense* and various other plants and phylogenetic relationships of caffeoyl-CoA O-methyltransferase proteins from *E*. *pseudowushanense* and various other plants. (DOCX)

S7 Fig Sequence alignment of chalcone synthase (CHS) proteins from *E*. *pseudowushanense* and various other plants, and phylogenetic relationships of chalcone synthase (CHS) proteins from *E*. *pseudowushanense* and various other plants. (DOCX)

S8 Fig Sequence alignment of chalcone isomerase (CHI) proteins from *E*. *pseudowushanense* and various other plants, and phylogenetic relationships of chalcone isomerase (CHI) proteins from *E*. *pseudowushanense* and various other plants. (DOCX)

S9 Fig Sequence alignment of leucoanthocyanidin dioxygenase proteins from *E*. *pseudowushanense* and various other plants, and phylogenetic relationships of leucoanthocyanidin dioxygenase proteins from *E*. *pseudowushanense* and various other plants. (DOCX)

S10 Fig Sequence alignment of flavonol synthase (FLS) proteins from *E*. *pseudowushanense* and various other plants, and phylogenetic relationships of flavonol synthase (FLS) proteins from *E*. *pseudowushanense* and various other plants. (DOCX)

S11 Fig Sequence alignment of flavonoid 3'-monooxygenase proteins from *E*. *pseudowushanense* and various other plants, and phylogenetic relationships of flavonoid 3'-monooxygenase proteins from *E*. *pseudowushanense* and various other plants. (DOCX)

S12 Fig Sequence alignment of anthocyanidin reductase proteins (ANR) from *E*. *pseudowushanense* and various other plants, and phylogenetic relationships of anthocyanidin reductase (ANR) proteins from *E*. *pseudowushanense* and various other plants. (DOCX)

S13 Fig Sequence alignment of naringenin 3-dioxygenase proteins from *E*. *pseudowushanense* and various other plants, and phylogenetic relationships of naringenin 3-dioxygenase proteins from *E*. *pseudowushanense* and various other plants. (DOCX)

S14 Fig Sequence alignment of bifunctional dihydroflavonol 4-reductase/flavanone 4-reductase proteins from *E*. *pseudowushanense* and various other plants, and phylogenetic relationships of bifunctional dihydroflavonol 4-reductase/flavanone 4-reductase proteins from *E*. *pseudowushanense* and various other plants. (DOCX)

S15 Fig Sequence alignment of trans-cinnamate 4-monooxygenase proteins from *E*. *pseudowushanense* and various other plants, and phylogenetic relationships of trans-cinnamate 4-monooxygenase proteins from *E*. *pseudowushanense* and various other plants. (DOCX)

S16 Fig Sequence alignment of shikimate O-hydroxycinnamoyltransferase proteins from *E*. *pseudowushanense* and various other plants, and phylogenetic relationships of shikimate O-hydroxycinnamoyltransferase proteins from *E*. *pseudowushanense* and various other plants. (DOCX)

S17 Fig Sequence alignment of coumaroylquinate(coumaroylshikimate) 3'-monooxygenase proteins from *E*. *pseudowushanense* and various other plants, and phylogenetic relationships of coumaroylquinate(coumaroylshikimate) 3'-monooxygenase proteins from *E*. *pseudowushanense* and various other plants. (DOCX)

S18 Fig Sequence length of TF unigenes. The X-axis shows the range of lengths of the transcript sequences. The Y-axis shows the number of unigenes.
(DOCX)

S19 Fig Distribution of TF family. The X-axis shows the type of transcription factor family. The Y-axis shows the number of unigenes.
(DOCX)

S1 File The unigene sequences. (FASTA)
