# Supplementary material for: Understanding the molecular mechanisms underlying the effects of light intensity on flavonoid production by RNA-seq analysis in Epimedium pseudowushanense B.L.Guo
Source: PLoS One. 2017 Aug 7;12(8):e0182348. doi: 10.1371/journal.pone.0182348 (PMC5546586; doi:10.1371/journal.pone.0182348)
Supplement: S2 Fig — Annotated unique sequences were classified into ‘Biological process’, ‘Cellular component’ and ‘Molecular function’. Panels (A), (B) and (C) are for different groups. (DOCX) [file pone.0182348.s016.docx]

**S2 Fig. GO classifications of DEGs between different light conditions. Annotated unique sequences were classified into ‘Biological process’, ‘Cellular component’ and ‘Molecular function’. Panels (A), (B) and (C) are for different groups.**

1. **L and M**

1. **M and H**

1. **L and H**
