# Supplementary material for: Understanding the molecular mechanisms underlying the effects of light intensity on flavonoid production by RNA-seq analysis in Epimedium pseudowushanense B.L.Guo
Source: PLoS One. 2017 Aug 7;12(8):e0182348. doi: 10.1371/journal.pone.0182348 (PMC5546586; doi:10.1371/journal.pone.0182348)
Supplement: S3 Fig — (DOCX) [file pone.0182348.s017.docx]

**S3 Fig. Putative flavonoid biosynthesis pathway of *E. pseudowushanense*.**
