# Supplementary material for: Understanding the molecular mechanisms underlying the effects of light intensity on flavonoid production by RNA-seq analysis in Epimedium pseudowushanense B.L.Guo
Source: PLoS One. 2017 Aug 7;12(8):e0182348. doi: 10.1371/journal.pone.0182348 (PMC5546586; doi:10.1371/journal.pone.0182348)

**S4 Fig. Sequence alignment of phenylalanine ammonia-lyase (PAL) proteins from *E. pseudowushanense* and various other plants, and phylogenetic relationships of phenylalanine ammonia-lyase (PAL) proteins from *E. pseudowushanense* and various other plants.**

* 20 * 40 * 60 * 80 * 100
P45726.pro : MDSTTAIG-----NGVGSGGSPGFCLK------------DPLNWGVAAEAMKGSHLEEVKGMVEEFRKPVVRLGGETLTISQVAAIAVR-G--SE-VAVE : 79
O64963.pro : MATNSIKQNG---HKNGSVELPELCIKK-----------DPLNWGVAAETLKGSHLDEVKRMVAEYRKPVVKLGGESLTISQVAAIATHDS---G-VKVE : 82
Q42667.pro : MELSHETCNGIKNDRNGGTSSLGLCTG-T----------DPLNWTVAADSLKGSHLDEVKRMIDEYRRPVVKLGGESLTIGQVTAIAAHDS---G-VKVE : 85
P45729.pro : MAYVNG-TT----NGHANGNGLDLCMKKE----------DPLNWGVAAEALTGSHLDEVKRMVAEYRKPVVKLEGETLTISQVAAISARDD--SG-VKVE : 82
P45730.pro : METVTKNG-----YQN--GSLESLCVNQR----------DPLSWGVAAEAMKGSHLDEVKRMVADYRKPVVKLGGETLTIAQVASIAGHDT---GDVKVE : 80
P24481.pro : MENGNGATT----NGHVNGNGMDFCMKTE----------DPLYWGIAAEAMTGSHLDEVKKMVAEYRKPVVKLGGETLTISQVAAISARDG--SG-VTVE : 83
P35513.pro : MAG-VA---------QNGHQEMDFCMK-V----------DPLNWEMAADSLKGSHLDEVKKMVAEFRKPVVKLGGETLTVAQVAAIAAKDN-VKT-VKVE : 77
O23865.pro : MDCENK--------NVVLGNG--LCMQK-----------DPLNWGMAAEALTGSHLDEVKRMVAEFRKPMVQLGGETLTVSQVAAIAAG-----S-VKVE : 73
P25872.pro : MASNGH---------VNGGENFELCKKSA----------DPLNWEMAAESLRGSHLDEVKKMVSEFRKPMVKLGGESLTVAQVAAIAVRDKSANG-VKVE : 80
P45728.pro : MENGNGAIT----NGHVNGNGMDFCMKTE----------DPLYWGIAAEAMTGSHLDEVKKMVAEYRKPVVKLGGETLTISQVAAISARDG--SG-VTVE : 83
TR2108|c0_ : MATTTTTVH----HQNGNGSIDGLCMKS-----------DPLNWGLAAESLQGSHLDEVKRMVNEFHKPIVKLEGQSLTISQVAAVAMR-G--DG-IEIE : 81
P27991.pro : MEATNG-------HQN-----GSFCLSTAKGN----N--DPLNWGAAAEAMKGSHLDEVKRMVAEYRKPVVRLGGETLTIAQVAAVAGHDH---G-VAVE : 78
O49835.pro : METIVE---------NGNGKTMEFCMK------------DPLNWEMASESMKGSHLDEVKNMVAEFRKPVVQLAGKTLTIGQVAAIAARDD---G-VTVE : 75
P45734.pro : MEVVAAAI-----LKNNINDYDSFCLTHANANNMKVNAADPLNWGVAAEAMKGSHLDEVKRMVEEYRKPVVRLGGETLTISQVAAIAAHD----G-ATVE : 90
Q01861.pro : METVAAAI-----TKN--NGYESFCVTNAKNNNMKVNSADPLNWGVAAEAMKGSHLDEVKRMVEEYRKPVVRLGGETLTISQVAAIAAHDH---G-VKVE : 89
TR575|c1_g : ----MGSLV----QEN---SIA-LCLNK-----------DPLNWNMAAESMKGSHLDEVKRMVEEYRKGVVRLGGESLTISQVAAIASH-D--QG-VKVE : 73
 m C DPLnW Aae 6 GSHLdEVK M6 e5r4p6V LgG 3LT6 QVaa6a g 6E

 * 120 * 140 * 160 * 180 * 200
P45726.pro : LSESAREGVKASSDWVMESMNKGTDSYGVTTGFGATSHRRTKEGGALQKELIRFLNAGIFGNGTES-CHTLPQSATRAAMLVRINTLLQGYSGIRFEILE : 178
O64963.pro : LSESARAGVKASSDWVMDSMSKGTDSYGVTTGFGATSHRRTKQGAALQKELIRFLNAGVFGSTKES-GHTLPHQATRAAMLVRINTLLQGYSGIRFEILE : 181
Q42667.pro : LAEAARAGVKASSDWVMDSMMKGTDSYGVTTGFGATSHRRTKQGGALQKELIRFLNSGIFGNGTES-SHTLPHSATRAAMLVRVNTLLQGYSGIRFEILE : 184
P45729.pro : LSEEARAGVKASSDWVMDSMNKGTDSYGVTTGFGATSHRRTKQGGALQKELIRFLNAGIFGSGAEAGNNTLPHSATRAAMLVRINTLLQGYSGIRFEILE : 182
P45730.pro : LSESARPGVKASSDWVMDSMDKGTDSYGVTTGFGATSHRRTKQGGALQKELIRFLNAGIFGNGTET-CHTLPHSATRAAMLVRINTLLQGYSGIRFEILE : 179
P24481.pro : LSEAARAGVKASSDWVMDSMNKGTDSYGVTTGFGATSHRRTKQGGALQKELIRFLNAGIFGNGSD---NTLPHSATRAAMLVRINTLLQGYSGIRFEILE : 180
P35513.pro : LSEGARAGVKASSDWVMDSMGKGTDSYGVTTGFGATSHRRTKNGGALQKELIRFLNAGVFGNGTES-CHTLPQSGTRAAMLVRINTLLQGYSGIRFEILE : 176
O23865.pro : LAESARAGVKASSDWVMESMNKGTDSYGVTTGFGATSHRRTKQGGALQKELIRFLNAGIFGSGNDS-SNILPHSATRAAMLVRINTLLQGYSGIRFEILE : 172
P25872.pro : LSEEARAGVKASSDWVMDSMNKGTDSYGVTTGFGATSHRRTKNGGALQKELIRFLNAGVFGNGTET-SHTLPHSATRAAMLVRINTLLQGYSGIRFEILE : 179
P45728.pro : LSEAARAGVKASSDWVMDSMNKGTDSYGVTTGFGATSHRRTKQGGALQKELIRFLNAGIFGNGSD---NTLPHSATRAAMLVRINTLLQGYSGIRFEILE : 180
TR2108|c0_ : LSESARAGVKASSDWVMDSMSKGTDSYGVTTGFGATSHRRTKQGGALQKELIRFLNAGVFGNGTES-THTLPQTATRAAMLVRINTLLQGYSGIRFEIME : 180
P27991.pro : LSESAREGVKASSEWVMNSMNNGTDSYGVTTGFGATSHRRTKQGGALQKELIRFLNAGIFGNGTES-SHTLPHTATRAAMLVRINTLLQGYSGIRFEILE : 177
O49835.pro : LAEAAREGVKASSDWVMDSMNKGTDSYGVTTGFGATSHRRTKQGGALQKELIRFLNAGIFGNGTET-SHTLPHSATRAAMLVRINTLLQGYSGIRFEILE : 174
P45734.pro : LSESARAGVKASSDWVMESMNKGTDSYGVTTGFGATSHRRTKQGGALQKELIRFLNAGIFGNGTES-NHTLPHTATRAAMLVRINTLLQGYSGIRFEILE : 189
Q01861.pro : LSESARAGVKASSDWVMESMNKGTDSYGVTTGFGATSHRRTKQGGALQKELIRFLNAGIFGNGTES-SHTLPHTATRAAMLVRINTLLQGYSGIRFEILE : 188
TR575|c1_g : LNESSRAGVKASSDWVMDSMNKGTDSYGVTTGFGATSHRRTNQGGALQKELIRFLNAGVFGNGTET-SHTLPHSATRAAMLVRINTLLQGYSGIRFEILE : 172
 L E aR GVKASSdWVM SM kGTDSYGVTTGFGATSHRRTk GgALQKELIRFLNaG6FGng e tLPh aTRAAMLVR6NTLLQGYSGIRFEI6E

 * 220 * 240 * 260 * 280 * 300
P45726.pro : AISKFLNNNITPCLPLRGTITASGDLVPLSYIAGLLTGRHNSKAVGPTGEILHPKEAFRLAGVEGGFFELQPKEGLALVNGTAVGSGLASMVLFEANILA : 278
O64963.pro : VITKFLNNNVTPCLPLRGTITASGDLVPLSYIAGMLTGRPNSKAVGPDGQTLSAAEAFEFVGINSGFFELQPKEGLALVNGTAVGSGLASTVLFDTNILA : 281
Q42667.pro : TITKFLNHNITPCLPLRGTITASGDLVPLSYIAGLLTGRPNSKAVGSNGQVLNPTEAFNLAGVTSGFFELQPKEGLALVNGTAVGSGLAATVLFEANILA : 284
P45729.pro : AITKFLNHNITPCLPLRGTITASGDLVPLSYIAGLLTGRPNSKAVGPTGVTLSPEEAFKLAGVEGGFFELQPKEGLALVNGTAVGSGMASMVLFEANILA : 282
P45730.pro : AITRLLNNNITPCLPLRGTITASGDLVPLSYIAGLLTGRPNSKATGPTGEVLDAAEAFKAAGIESGFFELQPKEGLALVNGTAVGSGLASMVLFETNVLA : 279
P24481.pro : AITKFLNQNITPCLPLRGTITASGDLVPLSYIAGLLTGRPNSKAVGPTGVILSPEEAFKLAGVEGGFFELQPKEGLALVNGTAVGSGMASMVLFEANILA : 280
P35513.pro : AITKLLNHNVTPCLPLRGTITASGDLVPLSYIAGLLTGRPNSKAVGPNGETLNAEEAFRVAGVNGGFFELQPKEGLALVNGTAVGSGLASMVLFDANVLA : 276
O23865.pro : AITKFLNQNITPCLPLRGTITASGDLVPLSYIAGLLTGRPNSKAVGPTGENLTAAEAFKLAGVDGGFFELQPKEGLALVNGTAVGSGMASMVLFETNILA : 272
P25872.pro : AITKLINSNITPCLPLRGTITASGDLVPLSYIAGLLTGRPNSKAVGPNGETLNAEEAFRVAGVNGGFFELQPKEGLALVNGTAVGSGMASMVLFDSNILA : 279
P45728.pro : AITKFLNQNITPCLPLRGTITASGDLVPLSYIAGLLTGRPNSKAVGPTGVILSPEEAFKLAGVEGGFFELQPKEGLALVNGTAVGSGMASMVLFEANILA : 280
TR2108|c0_ : AITKFLNNNITPCLPLRGTITASGDLVPLSYIAGILTGRPNSKAVGPKGEALTAQEAFNLAGIETGFFELQPKEGLALVNGTAVGSGLASMVLFDANILA : 280
P27991.pro : AITKLLNNNVTPCLDLRGTITASGDLVPLSYIAGLLTGRPNSKAVGPSGEVLNAKEAFELASINSEFFELQPKEGLALVNGTAVGSGLASMVLFEANILA : 277
O49835.pro : AITKFLNTNITPCLPLRGTITASGDLVPLSYIAGLLTGRPNSKAVGPTGEKINAEEAFRLAGISTGFFELQPKEGLALVNGTAVGSGMASMVLYEANILA : 274
P45734.pro : AITKLLNNNITPCLPLRGTITASGDLVPLSYIAGLLTGRSNSKAHGPSGEMLNAKEAFQLAGINAEFFELQPKEGLALVNGTAVGSGLASIVLFEANILA : 289
Q01861.pro : AITKLINNNVTPCL-LRGTITASGDLVPLSYIAGLLTGRPNSKAHGTSGEILNAKEAFQSAEINDGFFELQPKEGLALVNGTAVGSGLASIVLFEANILA : 287
TR575|c1_g : AITKFLNHNITPCLPLRGTITASGDLVPLSYIAGLLTGRPNSKAVGPKGEPLNAQEAFTLAGIDTGFFELQPKEGLAMVNGTAVGSGLASMVLFEANILA : 272
 aI34 6N N6TPCLpLRGTITASGDLVPLSYIAG6LTGRpNSKAvGp G 6 EAF ag6 gFFELQPKEGLA6VNGTAVGSG6As VL5 N6LA

 * 320 * 340 * 360 * 380 * 400
P45726.pro : VLSEVLSAIFAEVMQGKPEFTDHLTHKLKHHPGQIEAAAIMEHILDGSSYVKAAQKLHEMDPLQKPKQDRYALRTSPQWLGPLIEVIRSSTKSIEREINS : 378
O64963.pro : LLSEILSAIFAEVMQGKPEFTDHLTHKLKHHPGQIEAAAIMEHILDGSSYVKAAKKLHEQDPLQKPKQDRYALRTSPQWLGPQIEVIRYSTKSIEREIDS : 381
Q42667.pro : IMSEVLSAIFAEVMNGKPEFTDHLTHKLKHHPGQIEAAAIMEHILDGSSYVKAAQKLHETDPLQKPKQDRYALRTSPQWLGPQIEVIRAATKMIEREINS : 384
P45729.pro : VLAEVMSAIFAEVMQGKPEFTDHLTHKLKHHPGQIEAAAIMEHILDGSAYVKAAQKLHEMDPLQKPKQDRYALRTSPQWLGPQIEVIRSSTKMIEREINS : 382
P45730.pro : VLSELLSAIFAEVMNGKPEFTDHLTHKLKHHPGQIEAAAIMEHILDGSAYMKAAKKLHETDPLQKPKQDRYALRTSPQWLGPQIEVIRFSTKSIEREINS : 379
P24481.pro : VLAEVMSAIFAEVMQGKPEFTDHLTHKLKHHPGQIEAAAIMEHILDGSAYVKAAQKLHEMDPLQKPKQDRYALRTSPQWLGPQIEVIRSSTKMIEREINS : 380
P35513.pro : VFSEVLSAIFAEVMNGKPEFTDHLTHKLKHHPGQIEAAAIMEHILDGSSYVKAAQKLHETDPLQKPKQDRYALRTSPQWLGPQIEVIRSATKMIEREINS : 376
O23865.pro : VLAEVMSAIFAEVMQGKPEFTDHLTHKLKHHPGQIEAAAIMEHILDGSSYVKAAEKQHEMDPLQKPKQDRYALRTSPQWLGPQIEVIRSSTKMIEREINS : 372
P25872.pro : VMSEVLSAIFAEVMNGKPEFTDHLTHKLKHHPGQIEAAAIMEHILDGSSYVKAAQKLHEMDPLQKPKQDRYALRTSPQWLGPQIEVIRAATKMIEREINS : 379
P45728.pro : VLAEVMSAIFAEVMQGKPEFTDHLTHKLKHHPGQIEAAAIMEHILDGSAYVKAAQKLHEMDPLQKPKQDRYALRTSPQWLGPQIEVIRSSTKMIEREINS : 380
TR2108|c0_ : LLSEVISAIFAEVMQGKPEFTDHLTHKLKHHPGQIEAAAIMEHILDGSSYVKSAQKLHETDPLQKPKQDRYALRTSPQWLGPLIEVIRFSTKSIEREINS : 380
P27991.pro : VLSEVLSAIFAEVMQGKPEFTDHLTHKLKHHPGQIEAAAIMEHILDGSSYMKAAKKLHEIDPLQKPKQDRYALRTSPQWLGPLIEVIRFSTKSIEREINS : 377
O49835.pro : VLSEVISAIFAEVMNGKPEFTDHLTHKLKHHPGQIEAAAIMEHILDGSGYVKAAQKLHEMDPLQKPKQDRYALRTSPQWLGPQIEVIRSATKMIEREINS : 374
P45734.pro : VLSEVLSAIFAEVMQGKPEFTDHLTHKLKHHPGQIEAAAIMEHILHGSAYVKDAKKLHEMDPLQKPKQDRYALRTSPQWLGPLIEVIRFSTKSIEREINS : 389
Q01861.pro : VLSEVLSAIFAEVMQGKPEFTDHLTHKLKHHPGQIEAAAIMEHILDGSAYVKAAKKLHEMDPLQKPKQDRYALRTSPQWLGPLIEVIRFSTKSIEREINS : 387
TR575|c1_g : VLSEVISAIFAEVMQGKPEFTDHLTHKLKHHPGQIEAAAIMEHILTGSSYVKAAEKLHEMDPLQKPKQDRYALRTSPQWLGPLIEVIRSSTKSIEREINS : 372
 6 E66SAIFAEVM GKPEFTDHLTHKLKHHPGQIEAAAIMEHILdGS Y6KaA KlHE DPLQKPKQDRYALRTSPQWLGP IEVIR TK IEREI1S

 * 420 * 440 * 460 * 480 * 500
P45726.pro : VNDNPLINVSRNKALHGGNFQGTPIGVSMDNTRLAVASIGKLMFAQFSELVNDFYNNGLPSNLSGGRNPSLDYGFKGAEIAMAAYCSELQFLANPVTNHV : 478
O64963.pro : VNDNPLIDVSRNKALHGGNFQGTPIGVSMDNTRLAIASIGKLMFAQFSELVNDFYNNGLPSNLSGGRNPSLDYGFKGAEIAMASYCSELQFLANPVTNHV : 481
Q42667.pro : VNDNPLIDVSRNKALHGGNFQGTPIGVSMDNTRLAIASIGKLMFAQFSELVNDFYNNGLPSNLTGGRNPSLDYGFKGAEIAMASYCSELQFLANPVTNHV : 484
P45729.pro : VNDNPLIDVSRNKAIHGGNFQGSPIGVSMDNTRLAIAAIGKLMFAQFSELVNDFYNNGLPSNLSGGRNPSLDYGFKGAEIAMASYCSELQFLANPVTNHV : 482
P45730.pro : VNDNPLIDVSRNKAIHGGNFQGTPIGVSMDNVRLAIASIGKLLFAQFSELVNDFYNNGLPSNLTASRNPSLDYGFKGAEIAMASYCSELQYLANPVTTHV : 479
P24481.pro : VNDNPLIDVSRNKAIHGGNFQGTPIGVSMDNTRLAIAAIGKLMFAQFSELVNDFYNNGLPSNLSGGRNPSLDYGFKGAEIAMASYCSELQFLANPVTNHV : 480
P35513.pro : VNDNPLIDVSRNKALHGGNFQGTPIGVSMDNARLALASIGKLMFGQFSELVNDYYNNGLPSNLTAGRNPSLDYGFKGSEIAMASYCSELQFLANPVTNHV : 476
O23865.pro : VNDNPLIDVSRNKAIHGGNFQGTPIGVSMDNTRLAIAAIGKLMFAQFSELVNDFYNNGLPSNLSGGRNPSLDYGFKGAEIAMASYCSELQFLGNPVTNHV : 472
P25872.pro : VNDNPLIDVSRNKALHGGNFQGTPIGVSMDNARLALASIGKLMFAQFSELVNDYYNNGLPSNLTASRNPSLDYGFKGAEIAMASYCSELQFLANPVTNHV : 479
P45728.pro : VNDNPLIDVSRNKAIHGGNFQGTPIGMSMDNTRLAIAAIGKLMFAQFSELVNDFYNNGLPSNLSGGRNPSLDYGFKGAEIAMASYCSELQFLANPVTNHV : 480
TR2108|c0_ : VNDNPLIDVSRNKALHGGNFQGTPIGVSMDNTRLALASIGKLLFAQFSELVNDLYNNGLPSNLSGGRNPSLDYGMKGAEIAMASYCSELQFLANPVTNHV : 480
P27991.pro : VNDNPLIDVSRNKALHGGNFQGTPIGVSMDNTRLALASIGKLMFAQFSELVNDFYNNGLPSNLTASRNPSLDYGFKGAEIAMASYCSELQYLANPVTTHV : 477
O49835.pro : VNDNPLIDVSRNKALHGGNFQGTPIGVAMDNTRLAIASIGKLLFAQFSELVNDYYNNGLPSNLTGSRNPSLDYGFKGAEIAMASYCSELQFLANPVTNHV : 474
P45734.pro : VNDNPLIDVSRNKALHGGNFQGTPIGVSMDNTRLALASIGKLLFAQFSELVNDFYNNGLPSNLSASRNPSLDYGFKGSEIAMASYCSELQYLANPVTTHV : 489
Q01861.pro : VNDNPLIDVSRNKALHGGNFQGTPIGVSMDNTRLALASIGKLLFAQFSELVNDFYNNGLPSNLSASRNPSLDYGFKGSEIAMASYCSELQYLANPVTTHV : 487
TR575|c1_g : VNDNPLIDVSRNKALHGGNFQGTPIGVSMDNTRLAIGSIGKLLFAQFSELVNDFYNNGLPSNLSGGRNPSLDYGFKGAEIAMASYCSELQFLSNPVTNHV : 472
 VNDNPLI1VSRNKA6HGGNFQG3PIG6sMDNtRLA6a IGKL6FaQFSELVND YNNGLPSNL3 RNPSLDYGfKGaEIAMAsYCSELQ5LaNPVT HV

 * 520 * 540 * 560 * 580 * 600
P45726.pro : QSAEQHNQDVNSLGLISSRKTAEAVDILKLMSSTYLVALCQAVDLRHFEENLRNTVKSTVSQVAKRVLTMGVNGELHPSRFCEKDLLRVVDREYIFAYID : 578
O64963.pro : QSAEQHNQDVNSLGLISSRKTAEAVDILKLMSSTFLVALCQAIDLRHLEENLRNTVKNTVSQVAKRTLTTGVNGELHPSRFCEKDLLKVVDREYVFAYID : 581
Q42667.pro : QSAEQHNQDVNSLGLNSSRKTAEAVDILKLMSSTFLVALCQAIDLRHLEENLKNTVKNTVSQVAKRVLTMGVNGELHPSRFCEKDLIKVVDREYVFAYID : 584
P45729.pro : QSAEQHNQDVNSLGLISSRKTSEAVEILKLMSTTFLVGLCQAIDLRHLEENLKSTVKNTVSQVAKRVLTMGVNGELHPSRFCEKDLLRVVDREYIFAYID : 582
P45730.pro : QSAEQHNQDVNSLGLISSRKTAEAVDILKLMSTTFLVALCQAIDLRHLEENLKSAVKNTVSQVSKRVLTTGANGELHPSRFCEKELLKVVDREYVFAYVD : 579
P24481.pro : QSAEQHNQDVNSLGLISSRKTSEAVEILKLMSTTFLVGLCQAIDLRHLEENLKSTVKNTVSSVAKRVLTMGVNGELHPSRFCEKDLLRVVDREYIFAYID : 580
P35513.pro : QSAEQHNQDVNSLDLISARKTAEAVDILKLMSSTYLVALCQAIDLRHLEENLRNAVKNTVSQVAKRTLTMGTNGELHPSRFCEKDLLRVVDREYVFAYAD : 576
O23865.pro : QSAEQHNQDVNSLGLISSRKTAEAVEILKLMSTTFLVGLCQAVDLRHLEENLKSTVKNTVSQVAKKVLTMGVNGELHPSRFCELDLLRVVDREYIFAYID : 572
P25872.pro : QSAEQHNQDVNSLGLISARKTAEAVDILKLMSSTYLVALCQAIDLRHLEENLKNAVKNTVSQVAKRTLTMGANGELHPARFCEKELLRIVDREYLFAYAD : 579
P45728.pro : QSAEQHNQDVNSLGLISSRKTSEAVEILKLMSTTFLVGLCQAIDLRHLEENLKSTVKNTVSSVAKRVLTMGVNGELHPSRFCEKDLLRFVDREYIFAYID : 580
TR2108|c0_ : QSAEQHNQDVNSLGLISSRKTAEAVDILKLMSSTFLVGLCQAIDLRHLEENLKSAVKSTVSQVAKKVLTMGVNGELHPSRFCEKDLLQVVDREYVYAYID : 580
P27991.pro : QSAEQHNQDVNSLGLISSRKTNEAIEILKLMSSTFLIALCQAIDLRHLEENLKNSVKNTVSQVSKRILTTGVNGELHPSRFCEKDLLKVVDREYIFSYID : 577
O49835.pro : QSAEQHNQDVNSLGLISSRKTSEAVEILKLMSSSFLVALFQAVDLRHIEENVRLAVKNTVSQVAKRTLTTGVNGELHPSRFSEKDLLRVVDREYVFAYAD : 574
P45734.pro : QSAEQHNQDVNSLGLISSRKTKEAIEILQLMSSTFLIALCQAIDLRHLEENLKNSVKNTVSQVAKKTLTIGVSGELHPSRFCEKDLLKVVDREHVFSYID : 589
Q01861.pro : QSAEQHNQDVNSLGLISSRKTYEAIEILQLMSSTFLIALCQAVDLRHLEENLKNSVKNIVSQVAKRTLTTGVNGELHPSRFCEKDLLRVVDREHVFAYID : 587
TR575|c1_g : QSAEQHNQDVNSLGLISSRKTAESVEILKLMSSTFLVGLCQAIDLRFMEENLRSCVKSTVSQVAKKVLTMGVNGELHPSRFCEKDLLKVIDREYIFAYID : 572
 QSAEQHNQDVNSLgLiSsRKT Ea6 ILkLMS335L6 LcQA6DLRh EEN64 VKntVSqVaK4 LT GvnGELHPsRFcEkdL6 6DREy65aY D

 * 620 * 640 * 660 * 680 * 700
P45726.pro : DPCSATYPLMQKLRQVLVEHALKNGESEKNLSTSIFQKIRAFEEEIKTLLPKEVESTRAAIENGNSAIPNRIKECRSYPLYKFVREELGTELLTGEKVRS : 678
O64963.pro : DPCSATYPLMQKLRQVLVEHALTNGENEKNASTSIFQKIVAFEEELKVLLPKEVDSARAALDSGSAGVPNRITECRSYPLYKFVREELGAEYLTGEKVRS : 681
Q42667.pro : DPCSASSPLMQKLRQVLVDHALDNGDREKNSTTSIFQKIGAFEDELKTLLPKEVEIARTELESGNAAIPNRIKECRSYPLYKIVREDIGTSLLTGEKVRS : 684
P45729.pro : DPCSATYPLMQKLRETLVEHALNNGDKERNLSTSIFQKIAAFEDELKALLPKEVETARAALESGNPAIPNRIKECRSYPLYKFVREELGTEYLTGEKVRS : 682
P45730.pro : DPCSATYPLMQKLRQVFVDHALENGENEKNFSTSVFQKIEAFEEELKALLPKEVESARAAYDSGNSAIDNKIKECRSYPLYKFVREELGTVLLTGEKVQS : 679
P24481.pro : DPCSATYPLMQKLRQTLVEHALKNGDNERNLSTSIFQKIATFEDELKALLPKEVESARAALESGNPAIPNRIEECRSYPLYKFVRKELGTEYLTGEKVTS : 680
P35513.pro : DACSANYPLMQKLRQVLVDHALQNGENEKNANSSIFQKILAFEDELKAVLPKEVESARAALESGNPAIANRIKECRSYPLYRFVRGELGAELLTGEKVRS : 676
O23865.pro : DPCSATYPLMQKLRQVLVEHALKNGETEKNLSTSIFQKIAAFEDELKALLPKEVESARAVVESGNPAIPNRIKECRSYPLYKFIREELGTVYLTGEKVTS : 672
P25872.pro : DPCSCNYPLMQKLRQVLVDHAMNNGESEKNVNSSIFQKIGAFEDELKAVLPKEVESARAALESGNPAIPNRITECRSYPLYRFVRKELGTELLTGEKVRS : 679
P45728.pro : DPCSATYPLMQKLRQTLVEHALKNGDNERNMNTSIFQKIATFEDELKALLPKEVESARAALESGNPAIPNRIEECRSYPLYKFVRKELGIEYLTGEKVTS : 680
TR2108|c0_ : DPCSAVYPLMQKLRQVLVEHALSNGENEKNSSTSIFQKISLFEEELKLLLPKEVESARSAVESDNAAIPNQIKDCRSYPLYRFIRGELGTALLTGEKVVS : 680
P27991.pro : DPCSATYPLMQKLRQVLVDHALVNAECEKDVNSSIFQKIAIFEEELKNLLPKEVEGARAAYESGKAAIPNKIQECRSYPLYKFVREELGTGLLTGEKVRS : 677
O49835.pro : DPCLTTYPLMQKLRETLVGHALDNGENEKDVNTSIFHKIAIFEEELKAILPKEVENARASVENGIPAISNRIEECRSYPLYKFVREELGTELLTGEKVRS : 674
P45734.pro : DPCSATYPLAQKLRQVLVDHALVNGESEKNSNTSIFQKIATFEEELKTLLPKEVESARTAYENGNSTIANKINGCRSYPLYKFVREELGTSLLTGERVIS : 689
Q01861.pro : DPCSATYPLMQKLRQVLVDHALVNGESEKNLNTSIFQKIATFEDELKTLLPKEVESTRAAYESGNPTVPNKINGCRSYPLYRFVRQELGTGLLTGEKVIS : 687
TR575|c1_g : DPCSATYPLMQKLRQVLVDHALLNGQNEKDLNTSIFQKISTFEEELKALLPKEVESMRTAIESGNPLIPNRIKECRSYPLYKFVREELGTELLTGEKVRS : 672
TR575|c1_g : DpCsa yPLmQKLR2 lV HA6 Ng E41 3S6FqKI FE E6K 6LPKEVe aR a esgn 6 N I eCRSYPLY4f6R e6Gt LTGE4V S

 * 720 *
P45726.pro : PGEEFDKVFTALCKGEMIDPLMDCLKEWNGAPLPIC-- : 714
O64963.pro : PGEECDKVFTAICEGKIIDPILDCLEGWNGAPLPIC-- : 717
Q42667.pro : PGEEFDKVFTAMCEGKLIDPMLECLKEWNGAPLPICQN : 722
P45729.pro : PGEEFEKVFTAMSKGEIIDPLLECLESWNGAPLPIC-- : 718
P45730.pro : PGEEFDKVFTAMCQGKIIDPMLECLGEWNGSPLPIC-- : 715
P24481.pro : PGEEFEKVFIAMSKGEIIDPLLECLESWNGAPLPIC-- : 716
P35513.pro : PGEECDKVFTAMCNGQIIDSLLECLKEWNGAPLPIC-- : 712
O23865.pro : PGEEFDKVFTAMSKGEIIDPLLACLESWNGAPLPIA-- : 708
P25872.pro : PGEECDKVFTAMCNGQIIDPMLECLKSWNGAPLPIC-- : 715
P45728.pro : PGEEFDKVFIAMSKGEIIDPLLECLESWNGAPLPIC-- : 716
TR2108|c0_ : PGEEIDKVFTAMCEGKLIDPLLECLKEWNGAPLPIC-- : 716
P27991.pro : PGEEFDKLFTAMCQGKIIDPLMECLGEWNGAPLPIS-- : 713
O49835.pro : PGEELDKVFTAMCEGKLVDPLLACLEAWNGAPLPIC-- : 710
P45734.pro : PGEECDKLFTAMCQGKIIDPLLKCLGEWNGAPLPIC-- : 725
Q01861.pro : PGEECDKLFTAICQGKIIDPLLQCLGDWNGAPLPIS-- : 723
TR575|c1_g : PGEEIDKVFTAICQGKLIDPLLACVSEWNGSPLPIC-- : 708
 PGEE dK6FtA6 G 66Dp66 C6 WNGaPLPIc


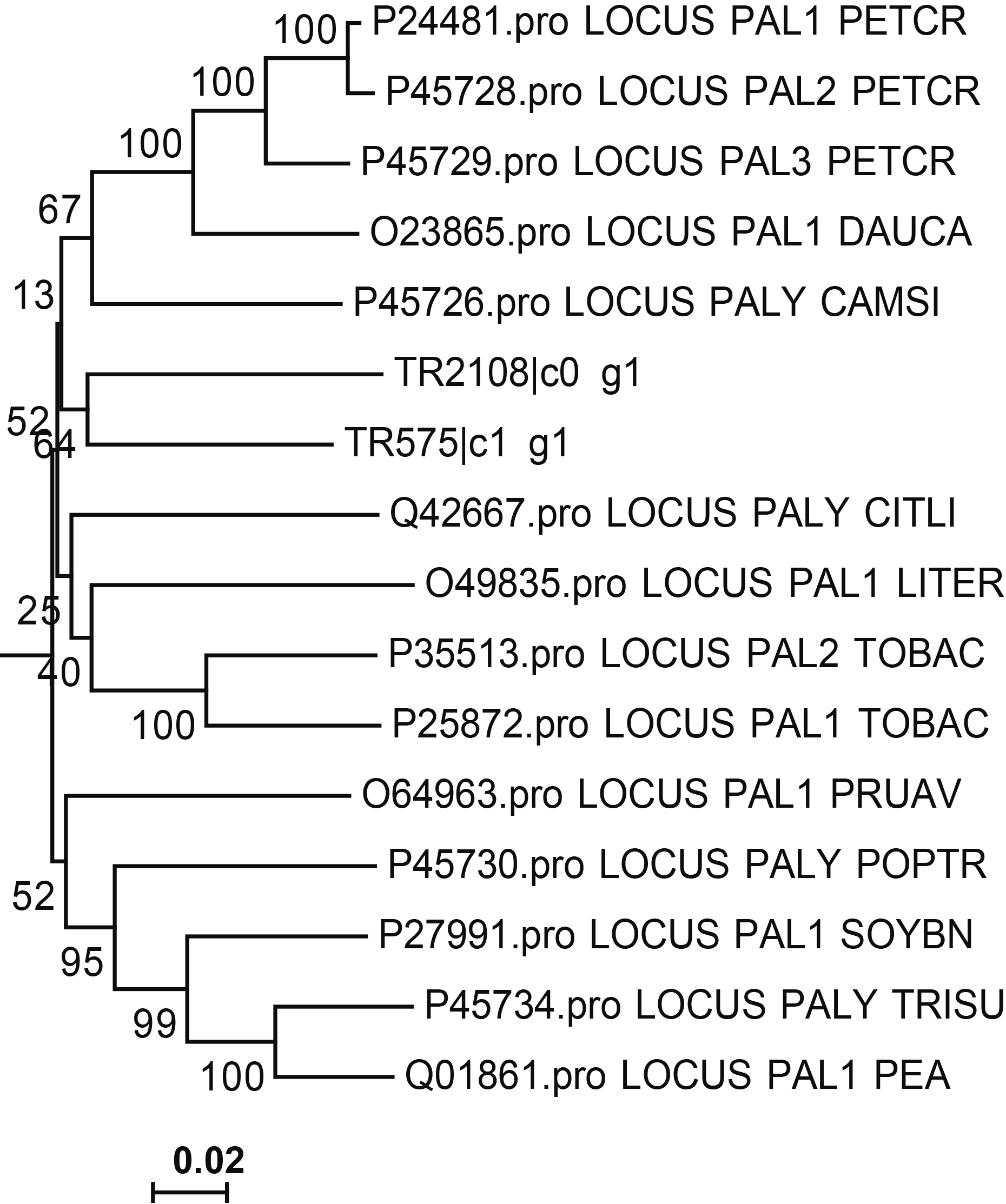

Supplement: S4 Fig — (DOCX) [file pone.0182348.s018.docx]
