# Supplementary material for: Understanding the molecular mechanisms underlying the effects of light intensity on flavonoid production by RNA-seq analysis in Epimedium pseudowushanense B.L.Guo
Source: PLoS One. 2017 Aug 7;12(8):e0182348. doi: 10.1371/journal.pone.0182348 (PMC5546586; doi:10.1371/journal.pone.0182348)

**S5 Fig. Sequence alignment of 4-coumarate-CoA ligase (4CL) proteins from *E. pseudowushanense* and various other plants, and phylogenetic relationships of 4-coumarate-CoA ligase (4CL) proteins from *E. pseudowushanense* and various other plants.**

* 20 * 40 * 60 * 80 * 100
Q9SEV0.pro : -----------------------------------------------MDQTLTHTGS-------KKACVIGGTGNLASILIKHLLQSGYKVNTTVR---- : 42
TR3386|c0_ : -----------------------------------------------MDTKPTVVS---------RTCVTGGTGFMASLLVKHLLEKGYAVNTTAR---- : 40
P31687.pro : MITLA-PSLDTPKTD-----QNQVSDPQ--TSHVFKSKLPDIPISNHLPLHSYCFQN--LSQFAHRPCLIVGPAS-KTFTYADTHLISSKIAAGLS--NL : 87
Q42982.pro : MITVAAPEAQP-QVAA------AVDEAPPEAVTVFRSKLPDIDIPSHLPLHEYCFAR--AAELPDAPCLIAAATG-RTYTFAETRLLCRRAAAALH--RL : 88
Q9S777.pro : MITAALHEPQIHKPTDTSVVSDDVLPHSPPTPRIFRSKLPDIDIPNHLPLHTYCFEK--LSSVSDKPCLIVGSTG-KSYTYGETHLICRRVASGLY--KL : 95
O24145.pro : -----MPMETT----------TETKQSG---DLIFRSKLPDIYIPKHLPLHSYCFEN--ISEFSSRPCLINGAND-QIYTYAEVELTCRKVAVGLN--KL : 77
P41636.pro : -------MANG----------IKKVEH------LYRSKLPDIEISDHLPLHSYCFER--VAEFADRPCLIDGATD-RTYCFSEVELISRKVAAGLA--KL : 72
O24146.pro : -----MEKD--------------TKQV----DIIFRSKLPDIYIPNHLPLHSYCFEN--ISEFSSRPCLINGANK-QIYTYADVELNSRKVAAGLH--KQ : 72
P31685.pro : -----MPMD------------IETKQSG---DLIFRSKLPDIYIPKHLPLHSYCFEN--LSEFNSRPCLIDGAND-RIYTYAEVELTSRKVAVGLN--KL : 75
P31684.pro : -----MPMD------------TETKQSG---DLIFRSKLPDIYIPKHLPLHSYCFEN--LSEFNSRPCLIDGAND-RIYTYAEVELTSRKVAVGLN--KL : 75
P14913.pro : -------MGDC----------VAPKE-----DLIFRSKLPDIYIPKHLPLHTYCFEN--ISKVGDKSCLINGATG-ETFTYSQVELLSRKVASGLN--KL : 73
O24540.pro : -------MAAA----------VAIEEQKK--DIIFRSKLPDIYIPKNLPLHSYCFEN--ISKFSSRPCLINGATD-EIFTYADVELISRRVGSGLS--KL : 76
P14912.pro : -------MGDC----------VAPKE-----DLIFRSKLPDIYIPKHLPLHTYCFEN--ISKVGDKSCLINGATG-ETFTYSQVELLSRKVASGLN--KL : 73
Q9S725.pro : MTTQDVIVNDQ----------NDQKQCSN--DVIFRSRLPDIYIPNHLPLHDYIFEN--ISEFAAKPCLINGPTG-EVYTYADVHVTSRKLAAGLH--NL : 83
Q42524.pro : MAPQEQAVSQV----------MEKQSNNNNSDVIFRSKLPDIYIPNHLSLHDYIFQN--ISEFATKPCLINGPTG-HVYTYSDVHVISRQIAANFH--KL : 85
Q9M0X9.pro : ----------------------MEKSGYG-RDGIYRSLRPTLVLPKDPNTSLVSFLFRNSSSYPSKLAIADSDTG-DSLTFSQLKSAVARLAHGFH--RL : 74
Q0DV32.pro : MASAS-----------------VPAAGYG-ADGVYRSLRPPAPVASDPGLSLTDLLLRRADACPSAVALADAAAGGRALTFAELRSAVLSTAVALSS-RA : 81
Q84P21.pro : MAS------------------VNSRSGFCNSNSTFYSKRTPIPLPPNPSLDVTTFIS--SQAHRGRIAFIDASTG-QNLTFTELWRAVESVADCLS--EI : 77
P0C5B6.pro : MAYPE------------RSLIVDPRSGFCKSNSTFYSKRQPLSLPPNLSRDVTTFIS--SQPHRGKTAFIDAATG-QCLTFSDLWRAVDRVADCLYH-EV : 84
Q3E6Y4.pro : MAYPE------------RDLIVDPRSGFCKSNSTFYSKRNPLCLPPNPSLDVTTFIS--SQPQRGTTAFIDASTG-HRLTFSDLWRVVDRVADCLYH-EV : 84
Q84P25.pro : MAVKHGVDGDGSEIES-RTLAVDRKSGFCESTSIFYSKREPMALPPNQFLDVTSFIA--SQPHRGKTVFVDAVTG-RRLSFPELWLGVERVAGCLY--AL : 94
Q10S72.pro : MGRSP-------------EMEVDARSGYCAATRTFRSRRADVPLPADPEVDVVSFLA--SRRHSGVVALVDAATG-RRITFTELWRAVAGAASALAAHPV : 84
Q84P24.pro : MAATHLHIPPNP--KT-QTSHQNPPFWFSSKTGIYTSKFPSLHLPVDPNLDAVSALF--SHKHHGDTALIDSLTG-FSISHTELQIMVQSMAAGIYH-VL : 93
TR10614|c0 : MISVASPEANTPQVVPQVIPTSDVETPLS-ETFIFRSKLPDITIPNHLPLHTYCFEN--ITEFADKPCLISGSNG-KIYTFEETHLICQKTAAGLS--KL : 94
TR1945|c0_ : -------METP----------AAPPSPPQ--EFIYRSKLPDIYIPNHLPLHSYCFEN--LSQFSSRPCLINGATG-EIQTYAEVELICRKVAKGLQ--NL : 76
TR9038|c0_ : ----------------------MEKSGYG-RDGIFRSLRPPLVLPKYPNHSMVPFLFRNSNSFQDKLALIDADSG-ETLTFSQSKSTVAKVAHGLL--QL : 74
 s p f a

 * 120 * 140 * 160 * 180 * 200
Q9SEV0.pro : -----------DPENEKKIAHLRKLQELGDLKIFKADLTDEDSFESSFSGCEYIFHVATPINFKSEDP-------------------------------- : 99
TR3386|c0_ : -----------DPENLKKVSHLLELQKLGDLKIFKADLTEEGSFEAAVADCDFVFHVATPVHFESEDP-------------------------------- : 97
P31687.pro : GILKGDVVMILLQNSADFVFSFLAISMIGAVATTANPFYTAPEIFKQFTVSKAKLIITQAMYVDKLRN-HDGA-----KLGED-FKVVTVDDP-----PE : 175
Q42982.pro : GVGHGDRVMVLLQNCVEFAVAFFAASFLGAVTTAANPFCTPQEIHKQFKASGVKLILTQSVYVDKLRQHEAFPRIDACTVGDDTLTVITIDDDE--ATPE : 186
Q9S777.pro : GIRKGDVIMILLQNSAEFVFSFMGASMIGAVSTTANPFYTSQELYKQLKSSGAKLIITHSQYVDKLKN-----------LGEN-LTLITTDEP----TPE : 179
O24145.pro : GIQQKDTIMILLPNSPEFVFAFMGASYLGAISTMANPLFTPAEVVKQAKASSAKIIITQSCFVGKVKDYA----------SENDVKVICID-----SAPE : 162
P41636.pro : GLQQGQVVMLLLPNCIEFAFVFMGASVRGAIVTTANPFYKPGEIAKQAKAAGARIIVTLAAYVEKLADLQ----------SH-DVLVITIDD----APKE : 157
O24146.pro : GIQPKDTIMILLPNSPEFVFAFIGASYLGAISTMANPLFTPAEVVKQAKASSAKIIVTQACHVNKVKDYA----------FENDVKIICID-----SAPE : 157
P31685.pro : GIQQKDTIMILLPNCPEFVFAFIGASYLGAISTMANPLFTPAEVVKQAKASSAKIVITQACFAGKVKDYA----------IENDLKVICVD-----SAPE : 160
P31684.pro : GIQQKDTIMILLPNCPEFVFAFIGASYLGAISTMANPLFTPAEVVKQAKASSAKIVITQACFAGKVKDYA----------IENDLKVICVD-----SVPE : 160
P14913.pro : GIQQGDTIMLLLPNSPEYFFAFLGASYRGAISTMANPFFTSAEVIKQLKASLAKLIITQACYVDKVKDYA----------AEKNIQIICID-----DAPQ : 158
O24540.pro : GIKQGDTIMILLPNSPEFVFAFLGASFIGSISTMANPFFTSTEVIKQAKASNAKLIITQGCYVDKVKDYA----------CENGVKIISIDTTTTADDAA : 166
P14912.pro : GIQQGDTIMLLLPNSPEYFFAFLGASYRGAISTMANPFFTSAEVIKQLKASQAKLIITQACYVDKVKDYA----------AEKNIQIICID-----DAPQ : 158
Q9S725.pro : GVKQHDVVMILLPNSPEVVLTFLAASFIGAITTSANPFFTPAEISKQAKASAAKLIVTQSRYVDKIKNLQ----------ND-GVLIVTTDSD---AIPE : 169
Q42524.pro : GVNQNDVVMLLLPNCPEFVLSFLAASFRGATATAANPFFTPAEIAKQAKASNTKLIITEARYVDKIKPLQ----------NDDGVVIVCIDDNESVPIPE : 175
Q9M0X9.pro : GIRKNDVVLIFAPNSYQFPLCFLAVTAIGGVFTTANPLYTVNEVSKQIKDSNPKIIISVNQLFDKIKGFDLP-----VVLLGS-KDTVEIPPG---SNSK : 165
Q0DV32.pro : GVRPGDAVLLLAPNCVLYPVCFFAVTALGAVGTTVNPDYTPREIAKQVSDARAKLVITISALVPKIAGLRLP-----VILLDDDANAAAASLP---PDAT : 173
Q84P21.pro : GIRKGHVVLLLSPNSILFPVVCLSVMSLGAIITTTNPLNTSNEIAKQIKDSNPVLAFTTSQLLPKISAAAKK----LPIVLMDEERVDSVGD------VR : 167
P0C5B6.pro : GIRRGDVVLILSPNSIFIPVVCLSVMSLGAVFTTANTLNTSGEISKQIADSNPTLVFTTRQLAPKLPVAISV----VLTD--DEVYQELTSA------IR : 172
Q3E6Y4.pro : GIRRGDVVLILSPNSIYIPVVCLSVMSLGAVVTTANTLNTSGEISKQIAQSNPTLVFTTSQLAPKLAAAISV----VLTDEEDEKRVELTSG------VR : 174
Q84P25.pro : GVRKGNVVIILSPNSILFPIVSLSVMSLGAIITTANPINTSDEISKQIGDSRPVLAFTTCKLVSKLAAASNFN---LPVVLMDDYHVPSQSYG---DRVK : 188
Q10S72.pro : SLRKGHVALILSPNSVHFPVAALAAMSLGAVLTTANPLNTPAEIAKQVADARPVLAFTTRELLPKLPRAHDL-----RVVLLESARLPGDSSD-----PR : 174
Q84P24.pro : GVRQGDVVSLVLPNSVYFPMIFLSLISLGAIVTTMNPSSSLGEIKKQVSECSVGLAFTSTENVEKLSSLGVS-----VISVSESYDFDS---------IR : 179
TR10614|c0 : GVKEGDVVMILLQNCPEFVFTFMGASMLGAITTTANPFYTTSEIFKQYHASGTKLIVTQSHYVDKLRGGEDCP-----KIGED-FLVVTVDDP-----PE : 183
TR1945|c0_ : GIKQHDVIMILLPNTPEFVFTFLGASYLGATTTTANPFYTPAEIAKQAKASNARLVITQASYVEKLTEFA----------SENNVKIVCTD-----SPPE : 161
TR9038|c0_ : GIKKGDVVLIFAPNSIQFPLCFLGIVAIGAIATTINPLYTSAEISKQVKDSGTKMVITVSQLWDKVKDFGLP-----AVILGSSRDTHQIV-----SRAR : 164
 g pn Ga t np e kq k

 * 220 * 240 * 260 * 280 * 300
Q9SEV0.pro : ------------------EKDMIKPAIQGVINVLKSCLKSKSVKRVIYTSSAAAVSINNLSGTGIVMNEEN---------------WTDVEFLTEEKPFN : 166
TR3386|c0_ : ------------------ENDMIKPAIQGTVDILRACAKAKTVKRVILTSSAAAVSINKLNGTGLVMNEEN---------------WTDVEFLASAKPPT : 164
P31687.pro : NCLHFSVLSEANESD----VPEVEIHPDDAVAMPFSSGTTGLPKGVILTHKSLTTSVAQQVDGENPNLYLT-TEDVLLCVLPLFHIFSLNSVLLCALRAG : 270
Q42982.pro : GCLPFWDLIADADEGS---VPEVAISPDDPVALPFSSGTTGLPKGVVLTHRSVVSGVAQQVDGENPNLHMG-AGDVALCVLPLFHIFSLNSVLLCAVRAG : 282
Q9S777.pro : NCLPFSTLITDDETNP--FQETVDIGGDDAAALPFSSGTTGLPKGVVLTHKSLITSVAQQVDGDNPNLYLK-SNDVILCVLPLFHIYSLNSVLLNSLRSG : 276
O24145.pro : GCLHFSELTQSDE----HEIPEVKIQPDDVVALPYSSGTTGLPKGVMLTHKGLVTSVAQQVDGENANLYMH-SEDVLMCVLPLFHIYSLNSILLCGLRVG : 257
P41636.pro : GCQHISVLTEADE----TQCPAVKIHPDDVVALPYSSGTTGLPKGVMLTHKGLVSSVAQQVDGENPNLYFH-SDDVILCVLPLFHIYSLNSVLLCALRAG : 252
O24146.pro : GCLHFSVLTQANE----HDIPEVEIQPDDVVALPYSSGTTGLPKGVMLTHKGLVTSVAQQVDGENPNLYIH-SEDVMLCVLPLFHIYSLNSVLLCGLRVG : 252
P31685.pro : GCVHFSELIQSDE----HEIPDVKIQPDDVVALPYSSGTTGLPKGVMLTHKGLVTSVAQQVDGENANLYMH-SDDVLMCVLPLFHIYSLNSVLLCALRVG : 255
P31684.pro : GCVHFSELIQSDE----HEIPDVKIQPDDVVALPYSSGTTGLPKGVMLTHKGLVTSVAQQVDGENANLYMH-SDDVLMCVLPLFHIYSLNSVLLCALRVG : 255
P14913.pro : DCLHFSKLMEADE----SEMPEVVIDSDDVVALPYSSGTTGLPKGVMLTHKGLVTSVAQQVDGDNPNLYMH-SEDVMICILPLFHIYSLNAVLCCGLRAG : 253
O24540.pro : NILHFSELTGADE----NEMPKVEISPDGVVALPYSSGTTGLPKGVMLTHKGLVTSVAQQVDGENPNLYMH-SDDVLLCVLPLFHIYSLNSVLLCGLRAG : 261
P14912.pro : DCLHFSKLMEADE----SEMPEVVINSDDVVALPYSSGTTGLPKGVMLTHKGLVTSVAQQVDGDNPNLYMH-SEDVMICILPLFHIYSLNAVLCCGLRAG : 253
Q9S725.pro : NCLRFSELTQSEEPR-VDSIP-EKISPEDVVALPFSSGTTGLPKGVMLTHKGLVTSVAQQVDGENPNLYFN-RDDVILCVLPMFHIYALNSIMLCSLRVG : 266
Q42524.pro : GCLRFTELTQSTTEA-SEVIDSVEISPDDVVALPYSSGTTGLPKGVMLTHKGLVTSVAQQVDGENPNLYFH-SDDVILCVLPMFHIYALNSIMLCGLRVG : 273
Q9M0X9.pro : ILSFDNVMELSEPVS---EYPFVEIKQSDTAALLYSSGTTGTSKGVELTHGNFIAASLMVTMDQDLMGEYH---GVFLCFLPMFHVFGLAVITYSQLQRG : 259
Q0DV32.pro : VTLYTNLVAGVKEA----DYRRPPIKQSDTAALLYSSGTTGDSKGVILTHRNFIAAARMVTSDQDERREGP---NVFLCFLPMFHIFGLSVITYAQLHRG : 266
Q84P21.pro : R---LVEMMKKEPSG---NRVKERVDQDDTATLLYSSGTTGMSKGVISSHRNLIAMVQTIVNRFGSD--DG--EQRFICTVPMFHIYGLAAFATGLLAYG : 257
P0C5B6.pro : VVGILSEMVKKEPSG---QRVRDRVNQDDTAMMLYSSGTTGPSKGVISSHRNLTAHVARFISDNLKR--D----DIFICTVPMFHTYGLLTFAMGTVALG : 263
Q3E6Y4.pro : VVGILSEMMKKETSG---QRVRDRVNQDDTAMMLYSSGTTGTSKGVISSHRNLTAYVAKYIDDKWKR--D----EIFVCTVPMFHSFGLLAFAMGSVASG : 265
Q84P25.pro : LVGRLETMIETEPSE---SRVKQRVNQDDTAALLYSSGTTGTSKGVMLSHRNLIALVQAYRARFGLE--Q-----RTICTIPMCHIFGFGGFATGLIALG : 278
Q10S72.pro : IVATIEEISATTPDP---ARRKDRVTQDDPATLLYSSGTTGPSKGVVATHRSLISMVQIIMTRFRLEGSDK--TETFLCTVPMFHVYGLVAFATGLLGCG : 269
Q84P24.pro : IENPKFYSIMKESFG---FVPKPLIKQDDVAAIMYSSGTTGASKGVLLTHRNLIASMELFVRFEASQYEYPGSSNVYLAALPLCHIYGLSLFVMGLLSLG : 276
TR10614|c0 : NCIHFSVVSESDEKE----VPVVTIDSDTAVALPFSSGTTGLPKGVILTHKSLISSVAQQVDGENPNLHLT-TEDIVLCVLPLFHIYSLNSVLLCSLRAG : 278
TR1945|c0_ : NCIHFSDLTQSDE----TQLPQVDIQPDDVVALPYSSGTTGLPKGVMLTHKGLVTSVAQQVDGENPNLYMN-SEDVLLCVLPMFHIYSLNSILLCGLRVG : 256
TR9038|c0_ : VTSFYELVNMAGDVS---GFPITSVKQTDTAALLYSSGTTGVSKGVVLTHRNFMAASLMAVSDQDFNGEGQ---NVFLCFLPMFHIFGLSICAYGQLQRG : 258
 d 6 ssgt g KgV l3h c p fh 5 l g

 * 320 * 340 * 360 * 380 * 400
Q9SEV0.pro : WGYPISKVLAEKTAWEFAKENKINLVTVIPALIAGNSLLSDPPS------SLSLSMS------------------------------------------- : 217
TR3386|c0_ : WGYPASKALAEKAAWKYAEENKIDLITVIPSLMAGPALTPSVPS------SICLAMS------------------------------------------- : 215
P31687.pro : SAVLLMQKFEIGTLLELIQRHRVSVAMVVPPLVLALAKNPMVAD--FDLSSIRLVLSGAAPLGKELEEALRNRMPQAVLGQGYGMTEAGPVLSMCLGFAK : 368
Q42982.pro : AAVALMPRFEMGAMLGAIERWRVTVAAVVPPLVLALAKNPFVER--HDLSSIRIVLSGAAPLGKELEDALRARLPQAIFGQGYGMTEAGPVLSMCPAFAK : 380
Q9S777.pro : ATVLLMHKFEIGALLDLIQRHRVTIAALVPPLVIALAKNPTVNS--YDLSSVRFVLSGAAPLGKELQDSLRRRLPQAILGQGYGMTEAGPVLSMSLGFAK : 374
O24145.pro : AAILIMQKFDIAPFLELIQKYKVSIGPFVPPIVLAIAKSPIVDS--YDLSSVRTVMSGAAPLGKELEDAVRTKFPNAKLGQGYGMTEAGPVLAMCLAFAK : 355
P41636.pro : AATLIMQKFNLTTCLELIQKYKVTVAPIVPPIVLDITKSPIVSQ--YDVSSVRIIMSGAAPLGKELEDALRERFPKAIFGQGYGMTEAGPVLAMNLAFAK : 350
O24146.pro : AAILIMQKFDIVSFLELIQRYKVTIGPFVPPIVLAIAKSPMVDD--YDLSSVRTVMSGAAPLGKELEDTVRAKFPNAKLGQGYGMTEAGPVLAMCLAFAK : 350
P31685.pro : AAILIMQKFDIAQFLELIPKHKVTIGPFVPPIVLAIAKSPLVHN--YDLSSVRTVMSGAAPLGKELEDAVRAKFPNAKLGQGYGMTEAGPVLAMCLAFAK : 353
P31684.pro : AAILIMQKFDIAQFLELIPKHKVTIGPFVPPIVLAIAKSPLVDN--YDLSSVRTVMSGAAPLGKELEDAVRAKFPNAKLGQGYGMTEAGPVLAMCLAFAK : 353
P14913.pro : VTILIMQKFDIVPFLELIQKYKVTIGPFVPPIVLAIAKSPVVDK--YDLSSVRTVMSGAAPLGKELEDAVRAKFPNAKLGQGYGMTEAGPVLAMCLAFAK : 351
O24540.pro : SGILIMQKFEIVPFLELIQKYKVTIGPFVPPIVLAIAKSTVVDN--YDLSSVRTVMSGAAPLGKELEDAVRAKFPNAKLGQGYGMTEAGPVLAMCLAFAK : 359
P14912.pro : VTILIMQKFDIVPFLELIQKYKVTIGPFVPPIVLAIAKSPVVDK--YDLSSVRTVMSGAAPLGKELEDAVRAKFPNAKLGQGYGMTEAGPVLAMCLAFAK : 351
Q9S725.pro : ATILIMPKFEITLLLEQIQRCKVTVAMVVPPIVLAIAKSPETEK--YDLSSVRMVKSGAAPLGKELEDAISAKFPNAKLGQGYGMTEAGPVLAMSLGFAK : 364
Q42524.pro : AAILIMPKFEINLLLELIQRCKVTVAPMVPPIVLAIAKSSETEK--YDLSSIRVVKSGAAPLGKELEDAVNAKFPNAKLGQGYGMTEAGPVLAMSLGFAK : 371
Q9M0X9.pro : NALVSMARFELELVLKNIEKFRVTHLWVVPPVFLALSKQSIVKK--FDLSSLKYIGSGAAPLGKDLMEECGRNIPNVLLMQGYGMTETCGIVSVEDPRLG : 357
Q0DV32.pro : NAIIAMSRFDINSLMEAVQRHRVTHLFCVPPVIIALAKHGKAGK--YDLSSLKFIGSGAAPLGKDVMEVVAKKFPDSEIVQGYGMTETCGIISLEYPEKG : 364
Q84P21.pro : STIIVLSKFEMHEMMSAIGKYQATSLPLVPPILVAMVNGADQIKAKYDLSSMHTVLCGGAPLSKEVTEGFAEKYPTVKILQGYGLTESTGIGASTDTVEE : 357
P0C5B6.pro : STVVILRRFQLHDMMDAVEKHRATALALAPPVLVAMINDADLIKAKYDLSSLKTVRCGGAPLSKEVTEGFLEKYPTVDILQGYALTESNGGGAFTNSAEE : 363
Q3E6Y4.pro : STVVILRRFGLDDMMQAVEKYKATILSLAPPVLVAMINGADQLKAKYDLTSLRKVRCGGAPLSKEVMDSFLEKYPTVNIFQGYALTESHGSGASTESVEE : 365
Q84P25.pro : WTIVVLPKFDMAKLLSAVETHRSSYLSLVPPIVVAMVNGANEINSKYDLSSLHTVVAGGAPLSREVTEKFVENYPKVKILQGYGLTESTAIAASMFNKEE : 378
Q10S72.pro : ATVVVLSKYELPEMLRSINAYGVTYLPLVPPILVAMVAHPKPLP----LGQMRKVLSGGAPLGKELIEGFREKYPQVEILQGYGLTESTAIGASTDSAEE : 365
Q84P24.pro : STIVVMKRFDASDVVNVIERFKITHFPVVPPMLMALTKKAKGVCG-EVFKSLKQVSSGAAPLSRKFIEDFLQTLPHVDLIQGYGMTESTAVGTRGFNSEK : 375
TR10614|c0 : AGVLIMPKFEIGSFLELIQRFRVSVAPVVPPLVLALAKNPMVEK--FDLSSIRIVLSGAAPLGKELENALKSRVPQAIFGQGYGMTEAGPVLSMCSGFAK : 376
TR1945|c0_ : AAILIMQKFDIVQLFELVQKYKVSIAPFVPPVVLAIVKNPASDN--YDLSTIRTVMSGAAPMGKELEDSLRTKLPNAKLGQGYGMTEAGPVLAMCLAFAK : 354
TR9038|c0_ : NTVISMERFDLEMVLKSIEKYRVTTLLVVPPVVIALAKQGAVKK--YDLSSLKQLCSGAAPLGKDLMEECAKILPQTEIIQGYGLTESCGIVSLENPKLG : 356
 Pp6 a d 6 sg ap p qgyg te

 * 420 * 440 * 460 * 480 * 500
Q9SEV0.pro : ------------FITGKEMHVT----G------------LKEMQKLSGSISFVHVDDLAR-----------------AHLFLAEKETASGRYICCAYNTS : 272
TR3386|c0_ : ------------LLTGNEFLIN----G------------MKGMQMLSGSISVTHVEDVVR-----------------AHVFLAEKESASGRYICCHINTS : 270
P31687.pro : QPFQTKSGSCGTVVRNAELKVVDPETGRSLGYNQPGEICIRGQQIMKGYLNDEAATASTIDSEGWLHTGDVGYVDDDDEIFIVDRVKELIKYKGFQVPPA : 468
Q42982.pro : EPTPAKSGSCGTVVRNAELKVVDPDTGFSLGRNLPGEICIRGPQIMKGYLNDPEATAATIDVEGWLHTGDIGYVDDDDEVFIVDRVKELIKFKGFQVPPA : 480
Q9S777.pro : EPIPTKSGSCGTVVRNAELKVVHLETRLSLGYNQPGEICIRGQQIMKEYLNDPEATSATIDEEGWLHTGDIGYVDEDDEIFIVDRLKEVIKFKGFQVPPA : 474
O24145.pro : EPFDIKSGACGTVVRNAEMKIVDPDTGCSLPRNQPGEICIRGDQIMKGYLNDPEATTRTIDKEGWLHTGDIGFIDEDDELFIVDRLKELIKYKGFQVAPA : 455
P41636.pro : NPFPVKSGSCGTVVRNAQIKILDTETGESLPHNQAGEICIRGPEIMKGYINDPESTAATIDEEGWLHTGDVEYIDDDEEIFIVDRVKEIIKYKGFQVAPA : 450
O24146.pro : EPFEIKSGACGTVVRNAEMKIVDPKTGNSLPRNQSGEICIRGDQIMKGYLNDPEATARTIDKEGWLYTGDIGYIDDDDELFIVDRLKELIKYKGFQVAPA : 450
P31685.pro : EPFDIKSGACGTVVRNAEMKIVDPDTGCSLPRNQPGEICIRGDQIMKGYLNDPEATARTIEKEGWLHTGDIGFIDDDDELFIVDRLKELIKYKGFQVAPA : 453
P31684.pro : EPFDIKSGACGTVVRNAEMKIVDPDTGCSLPRNQPGEICIRGDQIMKGYLNDPEATARTIEKEGWLHTGDIGFIDDDDELFIVDRLKELIKYKGFQVAPA : 453
P14913.pro : EPYEIKSGACGTVVRNAEMKIVDPETNASLPRNQRGEICIRGDQIMKGYLNDPESTRTTIDEEGWLHTGDIGFIDDDDELFIVDRLKEIIKYKGFQVAPA : 451
O24540.pro : EPFDIKSGACGTVVRNAEMKIVDPETGSSLPRNHPGEICIRGDQIMKGYLNDPEATARTIDKEGWLHTGDIGYIDDDDELFIVDRLKELIKYKGFQVAPA : 459
P14912.pro : EPYEIKSGACGTVVRNAEMKIVDPETNASLPRNQRGEICIRGDQIMKGYLNDPESTRTTIDEEGWLHTGDIGFIDDDDELFIVDRLKEIIKYKGFQVAPA : 451
Q9S725.pro : EPFPVKSGACGTVVRNAEMKILDPDTGDSLPRNKPGEICIRGNQIMKGYLNDPLATASTIDKDGWLHTGDVGFIDDDDELFIVDRLKELIKYKGFQVAPA : 464
Q42524.pro : EPFPVKSGACGTVVRNAEMKIVDPDTGDSLSRNQPGEICIRGHQIMKGYLNNPAATAETIDKDGWLHTGDIGLIDDDDELFIVDRLKELIKYKGFQVAPA : 471
Q9M0X9.pro : ---KRNSGSAGMLAPGVEAQIVSVETGKSQPPNQQGEIWVRGPNMMKGYLNNPQATKETIDKKSWVHTGDLGYFNEDGNLYVVDRIKELIKYKGFQVAPA : 454
Q0DV32.pro : Q--AREFGSTGTLVSGVEAKIVDIKTLKHLPPNQVGEICVRGPNVMQGYFNNVQATEFTI-KQGWLHTGDLGYFDGGGQLFVVDRLKELIKYKGFQIAPA : 461
Q84P21.pro : ---SRRYGTAGKLSASMEGRIVDPVTGQILGPKQTGELWLKGPSIMKGYFSNEEATSSTLDSEGWLRTGDLCYIDEDGFIFVVDRLKELIKYKGYQVAPA : 454
P0C5B6.pro : ---SRRYGTAGTLTSDVEARIVDPNTGRFMGINQTGELWLKGPSISKGYFKNQEATNETINLEGWLKTGDLCYIDEDGFLFVVDRLKELIKYKGYQVPPA : 460
Q3E6Y4.pro : ---SLKYGAVGLLSSGIEARIVDPDTGRVMGVNQPGELWLKGPSISKGYFGNEEATNETINLEGWLKLGDLCYIDEDGFLFVVDRLKELIKYKGYQVPPA : 462
Q84P25.pro : ---TKRYGASGLLAPNVEGKIVDPDTGRVLGVNQTGELWIRSPTVMKGYFKNKEATASTIDSEGWLKTGDLCYIDGDGFVFVVDRLKELIKCNGYQVAPA : 475
Q10S72.pro : ---SRRYGTAGLLSPNTEAKIVDPDSGEALPVNRTGELWIRGPYVMKGYFKNAEATQSTLTPDGWLKTGDLCYIDEDGYLFVVDRLKELIKYKGYQVPPA : 462
Q84P24.pro : ---LSRYSSVGLLAPNMQAKVVDWSSGSFLPPGNRGELWIQGPGVMKGYLNNPKATQMSIVEDSWLRTGDIAYFDEDGYLFIVDRIKEIIKYKGFQIAPA : 472
TR10614|c0 : QPFPTKSGSCGTVVRNAELKIIDPETGLSLKHNLPGEICIRGPQIMKGYLNDADSTAATIDVEGWLHTGDIGYVDDDEEVFIVDRVKELIKFKGFQVPPA : 476
TR1945|c0_ : EPFEIKSGSCGTVVRNAQMKIVDPETGASLPRNQPGELCIRGDQIMKGYVNDPEATAATIDKQGWLHTGDVGYIDDDDELFIVDRLKELIKYKGFQVAPA : 454
TR9038|c0_ : ---SRHSGSTGCLVPGVECQIVSTKTNKSLPPNQHGEIWIRGPNMMQGYFNNPEATSLTLDKQGWVHTGDLGYIDDLGQLFVVDRLKELIKYKGFQIAPA : 453
 g g 2 6 d g n ge 6 g kgy t gw tgd d 656vd4 ke i4 kg q pa

 * 520 * 540 * 560 * 580 *
Q9SEV0.pro : VPEIADFLIQRYPKYNVLSEFEEGLSIPKLTLSSQK-------------LINEGFRFEYGINEMYDQMIEYFESKGLIKAK-------------- : 340
TR3386|c0_ : IVELAKFLKKRYPQYNVPTDFGDFPEKAKLILTSDK-------------LRNEGFSFKYEIEDIYDQSIEYFKTVGLLDK--------------- : 337
P31687.pro : ELEGLLVSHPSIADAAVVPQKDVAAGEVPVAFVVRSNGFDLTEEAVKEFIAKQVVFYKRLHKVYFVHAIPKSPSGKILRKDLRAKLETAATQTP- : 562
Q42982.pro : ELESLLIAHPSIADAAVVPQKDDVAGEVPVAFVVRAADSDITEESIKEFISKQVVFYKRLHKVHFIHAIPKSASGKILRRELRAKLAAC------ : 569
Q9S777.pro : ELESLLINHHSIADAAVVPQNDEVAGEVPVAFVVRSNGNDITEEDVKEYVAKQVVFYKRLHKVFFVASIPKSPSGKILRKDLKAKLC-------- : 561
O24145.pro : EIEALLLNHPNISDAAVVPMKDEQAGEVPVAFVVRSNGSAITEDEVKDFISKQVIFYKRVKRVFFVETVPKSPSGKILRKDLRARLAAG-VPN-- : 547
P41636.pro : ELEALLVAHPSIADAAVVPQKHEEAGEVPVAFVVKS--SEISEQEIKEFVAKQVIFYKKIHRVYFVDAIPKSPSGKILRKDLRSRLAAK------ : 537
O24146.pro : ELEALLLNHPNISDAAVVPMKDEQAGEVPVAFVVRSNGSTITEDEVKDFISKQVIFYKRIKRVFFVDAIPKSPSGKILRKDLRAKLAAG-LPN-- : 542
P31685.pro : ELEALLINHPDISDAAVVPMIDEQAGEVPVAFVVRSNGSTITEDEVKDFISKQVIFYKRIKRVFFVETVPKSPSGKILRKDLRARLAAG-ISN-- : 545
P31684.pro : ELEALLINHPDISDAAVVPMIDEQAGEVPVAFVVRSNGSTITEDEVKDFISKQVIFYKRIKRVFFVETVPKSPSGKILRKDLRARLAAG-ISN-- : 545
P14913.pro : ELEALLLTHPTISDAAVVPMIDEKAGEVPVAFVVRTNGFTTTEEEIKQFVSKQVVFYKRIFRVFFVDAIPKSPSGKILRKDLRAKIASGDLPK-- : 544
O24540.pro : ELEALLLTHPCISDAAVVPMKDEAAGEVPVAFVVKSNGHNITEDEIKQFISKQVIFYKRINRVFFVEAIPKAPSGKILRKDLRARLAAAALPTN- : 553
P14912.pro : ELEALLLTHPTISDAAVVPMIDEKAGEVPVAFVVRTNGFTTTEEEIKQFVSKQVVFYKRIFRVFFVDAIPKSPSGKILRKDLRARIASGDLPK-- : 544
Q9S725.pro : ELESLLIGHPEINDVAVVAMKEEDAGEVPVAFVVRSKDSNISEDEIKQFVSKQVVFYKRINKVFFTDSIPKAPSGKILRKDLRARLANG-LMN-- : 556
Q42524.pro : ELEALLIGHPDITDVAVVAMKEEAAGEVPVAFVVKSKDSELSEDDVKQFVSKQVVFYKRINKVFFTESIPKAPSGKILRKDLRAKLANG-L---- : 561
Q9M0X9.pro : ELEGLLVSHPDILDAVVIPFPDEEAGEVPIAFVVRSPNSSITEQDIQKFIAKQVAPYKRLRRVSFISLVPKSAAGKILRRELVQQVRS----KM- : 544
Q0DV32.pro : ELEGLLLSHPEILDAVVIPFPDAKAGEVPIAYVVRSPDSSLTEVDVQKFIEKQVAYYKRLKRVTFVGSVPKSASGKILRRQLIAQVRS----SKL : 552
Q84P21.pro : ELEALLLTHPEITDAAVIPFPDKEVGQFPMAYVVRKTGSSLSEKTIMEFVAKQVAPYKRIRKVAFVSSIPKNPSGKILRKDLIKIATSNS--KL- : 546
P0C5B6.pro : ELEALLITHPDILDAAVIPFPDKEAGQYPMAYVVRKHESNLSEKQVIDFISKQVAPYKKIRKVSFINSIPKTASGKTLRKDLIKLATS----KL- : 550
Q3E6Y4.pro : ELEALLIAHPHILDAAVIPFPDREAGQYPMAYVARKPESNLSEKEVIDFISNQVAPYKKIRKVAFISSIPKTASGKTLRKDLIKLSTS----KL- : 552
Q84P25.pro : ELEALLLAHPEIADAAVIPIPDMKAGQYPMAYIVRKVGSNLSESEIMGFVAKQVSPYKKIRKVTFLASIPKNPSGKILRRELTKLTTS----KL- : 565
Q10S72.pro : ELEALLLTHPEVTDVAVIPFPDREVGQFPMAYIVRKKGSNLSEREVMEFVAKQVAPYKKVRKVAFVTDIPKNASGKILRKDLIKLATS----KL- : 552
Q84P24.pro : DLEAVLVSHPLIIDAAVTAAPNEECGEIPVAFVVRRQETTLSEEDVISYVASQVAPYRKVRKVVMVNSIPKSPTGKILRKELKRILTNSVSSRL- : 566
TR10614|c0 : ELEALLVNHPSIADAAVVPQKDDMTGEVPVAFVVPSNGFELSEDAVKEFIAKQVVFYKRLHKVYFIHAIPKSPSGKILRKDLRAKLASPPSS--- : 568
TR1945|c0_ : ELEALLVNHPNISDAAVVPMKDDAAGEIPVAFVVRLNGSQITEDEIKQYISKQVVFYKRIGKVFFVDSIPKAPSGKILRKDLRARLAAG-IPN-- : 546
TR9038|c0_ : ELEGLLLSHPEILDAVVIPFPDTEAGEVPIAYVVRSPSSSLNEEDVKKFIAKQVAPFKRLRKVSFIQNVPKSASGKILRRELIDKVRS----KM- : 543
 e E l hp daaV p g p6a v e 6 k2v 5 v 6pk gk 6 l


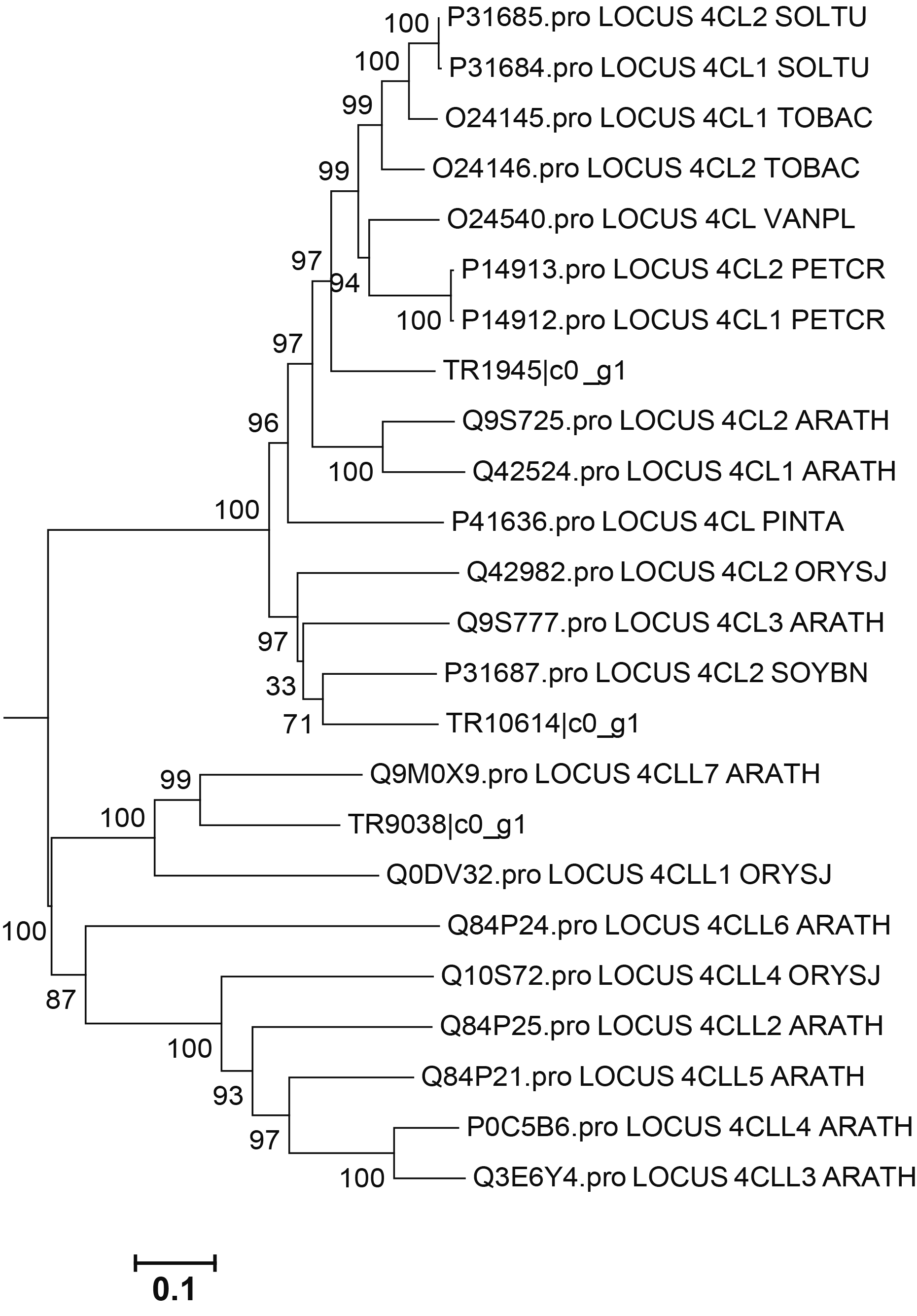

Supplement: S5 Fig — (DOCX) [file pone.0182348.s019.docx]
