# Supplementary material for: Understanding the molecular mechanisms underlying the effects of light intensity on flavonoid production by RNA-seq analysis in Epimedium pseudowushanense B.L.Guo
Source: PLoS One. 2017 Aug 7;12(8):e0182348. doi: 10.1371/journal.pone.0182348 (PMC5546586; doi:10.1371/journal.pone.0182348)

**S6 Fig. Sequence alignment of caffeoyl-CoA O-methyltransferase proteins from *E. pseudowushanense* and various other plants and phylogenetic relationships of caffeoyl-CoA O-methyltransferase proteins from *E. pseudowushanense* and various other plants.**

* 20 * 40 * 60 * 80 * 100
Q8H9B6.pro : MASNGE------------NGRHQEVGHKSLLQSDALYQYILETSVYPREPEAMKELREITAKHP---WNLMTTSADEGQFLNMLLKLINAKNTMEIGVFT : 85
Q43095.pro : MATNGEEQQS-------QAGRHQEVGHKSLLQSDALYQYILETSVYPREPECMKELREVTAKHP---WNIMTTSADEGQFLNMLLKLVNAKNTMEIGVYT : 90
O65922.pro : MAANGEEQQT-------QAGRHQEVGHKSLLQSDALYQYILETSVYPREPECMKELRELTAKHP---WNIMTTSADEGQFLNMLLKLINAKNTMEIGVFT : 90
O65862.pro : MATNGEEQQS-------QAGRHQEVGHKSLLQSDALYQYILETSVYPREPECMKELREVTAKHP---WNIMTTSADEGQFLNMLLKLVNAKNTMEIGVYT : 90
O24144.pro : MAT---------------NGRHQEVGHKSLLQSDALYQYILETSVYPREPEPMKELREITAKHP---WNLMTTSADEGQFLSMLIKLINAKNTMEIGVFT : 82
O24151.pro : MATDGE------------NGRHQEVGHKSLLQSDALYQYILETSVYPREPEPMKELREITAKHP---WNLMTTSADEGQFLSMLIKLINAKNTMEIGVFT : 85
O24149.pro : MATNGE------------NGRHQEVGHKSLLQSDALYQYILETSVYPREPEPMKELREITAKHP---WNLMTTSADEGQFLSMLLKLINAKNTMEIGVFT : 85
Q43237.pro : MATNQE------------AGRHQEVGHKSLLQSDALYQYILETSVYPREPESMKELRELTAQHP---WNIMTTSADEGQFLNMLLKLINAKNTMEIGVYT : 85
O24150.pro : MATNGE------------NGRHQEVGHKSLLQSDALYQYILETSVYPREPEPMKELREITAKHP---WNIMTTSADEGQFLSMLLKLINAKNTMEIGVFT : 85
P28034.pro : MASNGES-------------KHSEVGHKSLLQSDALYQYILETSVYPREPEAMKELREVTAKHP---WNLMTTSADEGQFLNMLLKLINAKNTMEIGVYT : 84
Q9C9W3.pro : MANEIP----------------TKG----ILKSEALKQYIMETSAYPREHELLKELRKATVQKYG-NLSEMEVPVDEGHFLSMLVKIMNAKNTIEIGVFT : 79
P93711.pro : MAFVLP----------------AKG----ILQSEALKQYIYETSAYPGEHEQLKELREATTKKYG-SLSGMSVPVDEGRFLSMLLKLMNAKRTLEVGVFT : 79
Q9C9W4.pro : MDGRLP----------------DKG----ILKSEALKQYIMETTAYPREHELLKELREATIQRYG-NLSEMGVPVDESLFLSMLVKIINAKNTIEIGVFT : 79
Q43161.pro : MLTKTMG------------NFFTEVKDTGLLQSEQLHQYILDTSVFPRESEHLKELRKATESHP---MSFMGTSPLAGQLLSFMLKTVKPKKTIEVGVFT : 85
Q9C5D7.pro : MAKD-EA--------------------KGLLKSEELYKYILETSVYPREPEVLRELRNITHNHP---QAGMATAPDAGQLMGMLLNLVNARKTIEVGVFT : 76
Q40313.pro : MATNEDQKQT-------ESGRHQEVGHKSLLQSDALYQYILETSVFPREHEAMKELREVTAKHP---WNIMTTSADEGQFLSMLLKLINAKNTMEIGVYT : 90
C7AE94.pro : MSSSSH----------------RG-----ILKTEALTKYLLETSAYPREHEQLKGLREATVEKHK-YWSLMNVPVDEGLFISMLLKIMNAKKTIELGVFT : 78
O04854.pro : MATAGEESQT-------QAGRHQEVGHKSLLQSDALYQYILETSVYPREPEPMKELREITAKHP---WNIMTTSADEGQFLNMLLKLINAKNTMEIGVFT : 90
TR10281|c0 : MATNQSEQTTTTTTTTTQAMRHAEVGHKSLLQSDALYQYILETSVYPREPQAMKELREITANHP---WNLMTTSADEGQFLAMLLKLINAKNTMEIGVFT : 97
TR1231|c0_ : MDTSHT----------------KC-----LLQSDALQQYILETSVYPREHEQLKELRKATFEKYEPRKASMEIPADEGQLLSMLLKMMNAKKTIEIGVFT : 79
TR17306|c0 : MASNSS----------------DGAGYTIILQSNNLLQYILKTSVYPREDEHLKKIREATSMTFNDYRSTMYLAPDEGQFLSMLLKIMNAKKTLEIGVFT : 84
 Ma 6Lq3 aL qY6 eT3v5PrE 2 64e6Re T M degqf6 m66k 6na4 T6E6GV5T

 * 120 * 140 * 160 * 180 * 200
Q8H9B6.pro : GYSLLATAMALPDDGKILAMDINRENYEIGLPVIEKAGLAHKIDFREGPALPVLDQMIEDGKYHGSYDFIFVDADKDNYLNYHKRLIDLVKVGGLIGYDN : 185
Q43095.pro : GYSLLATALAIPEDGKILAMDINRENYELGLPVIQKAGVAHKIDFKEGPALPVLDQMIEDGKYHGSFDFIFVDADKDNYINYHKRLIELVKVGGLIGYDN : 190
O65922.pro : GYSLLATALAIPEDGKILAMDINRENYELGLPVIQKAGLEHKIEFKEGPALPVLDQMIEDGKYHGTYDFIFVDADKDNYINYHKRLIELVKVGGLIGYDN : 190
O65862.pro : GYSLLATALAIPEDGKILAMDINRENYELGLPVIQKAGVAHKIDFKEGPALPVLDQMIEDGKCHGSFDFIFVDADKDNYINYHKRLIELVKVGGLIGYDN : 190
O24144.pro : GYSLLATAMALPDDGKILAMDINRENYEIGLPVIEKAGLAHKIEFKEGPALPVLDQMIEDGKYHGSYDFIFVDADKDNYLNYHKRLIDLVKIGGLIGYDN : 182
O24151.pro : GYSLLATAMALPDDGKILAMDINRENYEIGLPVIEKAGLAHKIEFKEGPALPVLDQMIEDGKYHGSYDFIFVDADKDNYLNYHKRLIDLVKIGGLIGYDN : 185
O24149.pro : GYSLLATAMALPDDGKILAMDINRENYEIGLPIIEKAGLAHKIVFREGPALPVLDQMIEDGKYHGSYDFIFVDADKDNYLNYHKRLIDLVKVGGLIGYDN : 185
Q43237.pro : GYSLLATALALPDDGKILAMDINKENYELGLPVIQKAGVAHKIDFKEGPALPVLDQMIEDGKYHGSFDFIFVDADKDNYLNYHKRLIDLVKVGGIIGYDN : 185
O24150.pro : GYSLLATAMALPDDGKILAMDINRDNYEIGLPVIEKAGLAHKIEFKEGPALPVLDQMIEDGKYHGSYDFIFVDADKDNYLNYHKRLIDLVKVGGLIGYDN : 185
P28034.pro : GYSLLATALALPDDGKILAMDINRENYEIGLPIIEKAGVGHKIDFREGPALPVLDHMLEDGKYHGTFDFVFVDADKDNYINYHKRLIDLVKIGGLIGYDN : 184
Q9C9W3.pro : GYSLLTTALALPEDGRITAIDIDKEAYEVGLEFIKKAGVDHKINFIHSDGLKALDQLVN-DK-C-EFDFAFADADKSSYVNFHERLLKLVKVGGIIAFDN : 176
P93711.pro : GYSLLSTALALPEDGQVTAIDKDRGAYEIGLPFIQKAGVEDKINFIQSEAPPILNEMLCNDK-QPEFDFAFVDADKSSYKHYHEQLLKLVKIGGIIAYDN : 178
Q9C9W4.pro : GYSLFTVALALPEDGRITAIDIDQAGYNLGLEFMKKAGVDHKINFIQSDAVRGLDQLLNGEK-Q-EYDFAFVDADKTNYVYFLEKLLKLVKVGGIIAFDN : 177
Q43161.pro : GYSLLATALSIPDDGKITAVDIDREAYNVGLALIKKAGVESKISFIVSDAMTLLDDLLADGRYQGSYDFAFVDADKTNYVNYHERLIELVKVGGIIAYDN : 185
Q9C5D7.pro : GYSLLLTALTLPEDGKVIAIDMNRDSYEIGLPVIKKAGVEHKIDFKESEALPALDELLNNKVNEGGFDFAFVDADKLNYWNYHERLIRLIKVGGIIVYDN : 176
Q40313.pro : GYSLLATALAIPEDGKILAMDINKENYELGLPVIKKAGVDHKIDFREGPALPVLDEMIKDEKNHGSYDFIFVDADKDNYLNYHKRLIDLVKVGGVIGYDN : 190
C7AE94.pro : GYSLLATALALPQDGKIIAVDPDKEAYQTGVPFIKKAGVEHKINFIQSDAMSVLNDLIADGKEEGTLDFAMVDADKENYLNYHELLLKLVRVGGIIAYDN : 178
O04854.pro : GYSLLATALALPDDGKILAMDINRENYELGLPVIQKAGVADKIDFREGPALPILDQLIEDGK-QGSFDFIFVDADKDNYLNYHKRLIELVKVGGLIGYDN : 189
TR10281|c0 : GYSLLATALALPEDGKILAMDINRENYEIGLPVIEKAGVAHKIDFREGPALPVLDQMIEDGKFHGSYDFIFVDADKDNYINYHKRLIDLVKIGGVIGYDN : 197
TR1231|c0_ : GYSLLATALALPEDGKIIAIDPDKEAYELGLPFIKKAGVEHKINYIQSDAISVLKDLLSNDKHEWEFDFAFVDADKINYINYYEHLMKLVKVGGTIAFDN : 179
TR17306|c0 : GYSLLTTALALPEDGKITAIDPDRESYEIGLPFIKMAGVEHKIDFIQSDAMSVIHDMLKDGK-ESEFDFVFVDADKPNYPNYHDLLMKLVKVGGIIAYDN : 183
 GYSLl tA6a6P DG 6 A6D 1 e Y G6p 6 kAG6 hKI 5 a 6 66 DF fvDADK nY n5h L6 L646GG I 5DN

 * 220 * 240 * 260
Q8H9B6.pro : TLWNGSVVAPPDAPLRKYVRYYRDFVLELNKALAADPRIEICQLPVGDGITLCRRIS--- : 242
Q43095.pro : TLWNGSVVAPPDAPMRKYVRYYRDFVLELNKALAADPRIEICMLPVGDGITLCRRIQ--- : 247
O65922.pro : TLWNGSVVAPADAPMRKYVRYYRDFVLELNKALAADPRIEICMLPVGDGITLCRRIK--- : 247
O65862.pro : TLWNGSVVAPPDAPMRKYVRYYRDFVLELNKALAADPRIEICMLPVGDGITLCRRIQ--- : 247
O24144.pro : TLWNGSVVAPPDAPLRKYVRYYRDFVLELNKALAADSRIEICQLPVGDGITLCRRIS--- : 239
O24151.pro : TLWNGSVVAPPDAPLRKYVRYYRDFVLELNKALAADSRIEICQLPVGDGITLCRRIS--- : 242
O24149.pro : TLWNGSVVAPPDAPLRKYVRYYRDFVLELNKALAADSRIEICQLPVGDGITLCRRIS--- : 242
Q43237.pro : TLWNGSVVAPPDAPLRKYVRYYRDFVLELNKALAADPRIEICMLPVGDGITLCRRLS--- : 242
O24150.pro : TLWNGSVVAPPDAPLRKYVRYYRDFVLELNKALAADSRIEICQLPVGDGITLCRRIS--- : 242
P28034.pro : TLWNGSVAQPADAPMRKYVRYYRDFVIELNKALAADPRIEICMLPVGDGVTLCRRIS--- : 241
Q9C9W3.pro : TLWFGFVAED-EDGVPEHMREYRAALIEFNKKLALDPRVEVSQISIGDGITLCRRLV--- : 232
P93711.pro : TLWYGLVAKEVDDEVPEPLRMVRTVIMEFNKLLSSDLRVEISQISIGDGVTLCRRLC--- : 235
Q9C9W4.pro : TLWFGTLIQK-ENEVPGHMRAYREALLEFNKILARDPRVEIAQISIGDGLTLCRRLI--- : 233
Q43161.pro : TLWGGTVALP-ESEVPDFMKNNWVCVTKLNEILGSDARIDIAHLPVGDGITFCRRVY--- : 241
Q9C5D7.pro : TLWGGSVAEP-DSSTPEWRIEVKKATLELNKKLSADQRVQISQAALGDGITICRRLY--- : 232
Q40313.pro : TLWNGSVVAPPDAPLRKYVRYYRDFVLELNKALAVDPRIEICMLPVGDGITICRRIK--- : 247
C7AE94.pro : TLWFGSVARSEEEEMMDFERAGRVHLMKLNKFLASDPRVELSHLSIGDGVALCRRLY--- : 235
O04854.pro : TLWNGSVVAPPDAPLRKYVRYYRDFVLELNKALAADPRIEICMLPVGDGITLCRRIQLSI : 249
TR10281|c0 : TLWSGSVVAPADAPMRKYVRYYRDFVLELNKALAADPRIEICQLPVGDGITLCRRIS--- : 254
TR1231|c0_ : TLWFGSVALKDGDEIPEQLKPGRVHTIKLNSYLAADPRIEISQVPIGDGVTLCRRLY--- : 236
TR17306|c0 : TLWFGTVG-HAEDEVRESVRVDRKTIMELNSLVASDPRVELSHLSIGDGLTLCKRLY--- : 239
 TLW G 6 elNk 6a D R6 6 6GDG6t C4R6


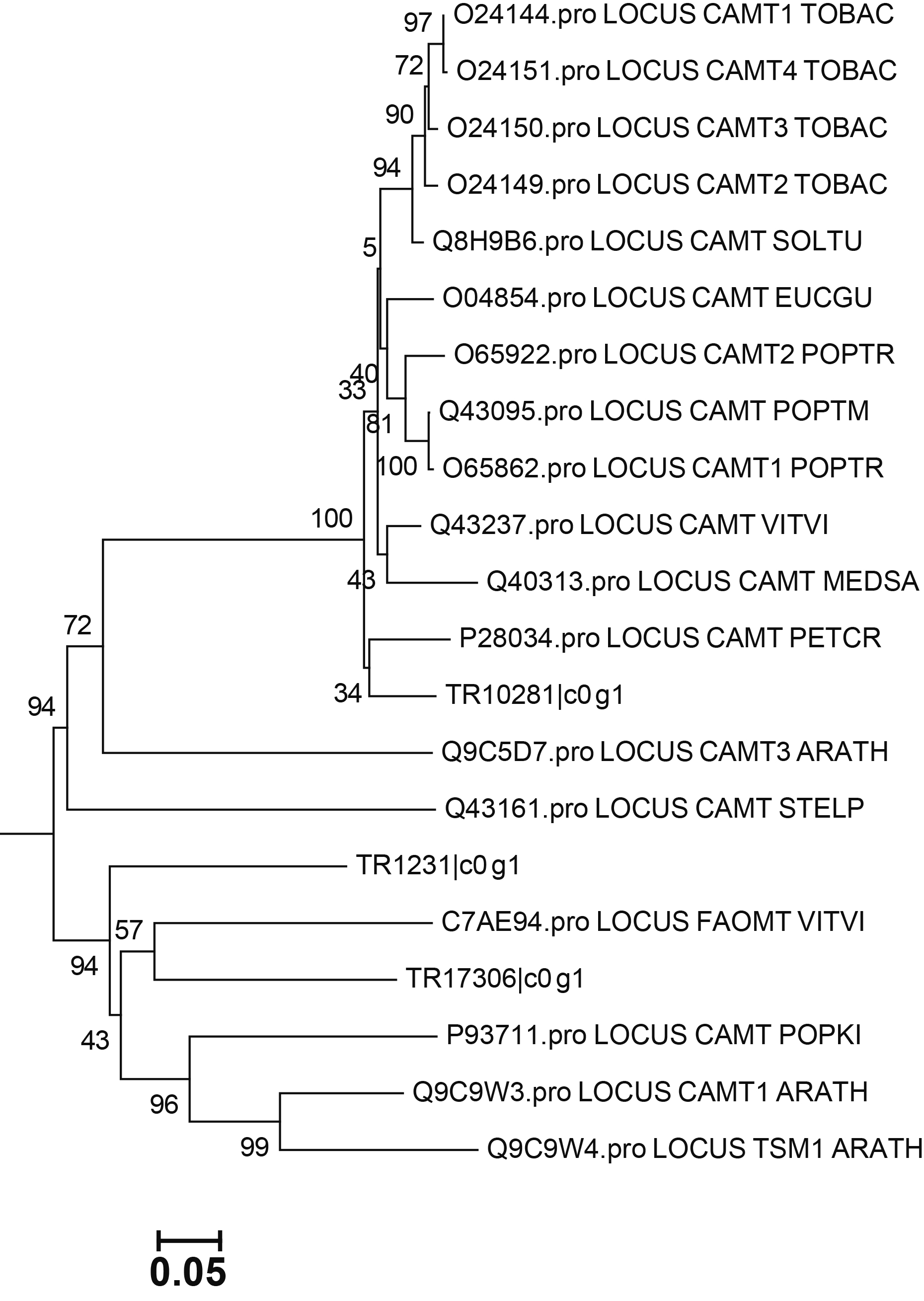

Supplement: S6 Fig — (DOCX) [file pone.0182348.s020.docx]
