# Supplementary material for: Understanding the molecular mechanisms underlying the effects of light intensity on flavonoid production by RNA-seq analysis in Epimedium pseudowushanense B.L.Guo
Source: PLoS One. 2017 Aug 7;12(8):e0182348. doi: 10.1371/journal.pone.0182348 (PMC5546586; doi:10.1371/journal.pone.0182348)

**S7 Fig. Sequence alignment of chalcone synthase (CHS) proteins from *E. pseudowushanense* and various other plants, and phylogenetic relationships of chalcone synthase (CHS) proteins from *E. pseudowushanense* and various other plants.**

* 20 * 40 * 60 * 80 * 100
P51090.pro : ----MVS-----VAEIRKAQRAEGPATVLAIGTATPANCVYQADYPDYYFRITNSEHMTELKEKFKRMCEKSMINKRYMHLTEEILKENPNVCAYMAPSL : 91
P51075.pro : ----MAS-----VEEIRKAQRAHGPATVLAIGTATPSNCITQADYPDYYFRITKSDHMTELKEKFKRMCDKSMIKKRYMYLNEEILNENPNMCAYMAPSL : 91
P48385.pro : ----MAS--TIDIAAIREAQRRQGPATILAIGTATPSNCVYQADYPDYYFRITKSEHMVDLKEKFKRMCDKSMIRKRYMHLTEEYLKENPSLCEYMAPSL : 94
Q9XJ57.pro : ----MAT-----VQEIRNAQRADGPATVLAIGTATPAHSVNQADYPDYYFRITKSEHMTELKEKFKRMCDKSMIKKRYMYLTEEILKENPNMCAYMAPSL : 91
P06515.pro : ----MVT-----VEEVRRAQRAEGPATVLAIGTATPANCVDQSTYPDYYFRITNSEHMTELKEKFKRMCDKSNIKRRYMHLTEEILKENPAMCEYMAPSL : 91
P48390.pro : ----MAS--SVDMKAIRDAQRAEGPATILAIGTATPANCVYQADYPDYYFRITKSEHMVDLKEKFKRMCDKSMIRKRYMHITEEYLKQNPNMCAYMAPSL : 94
Q9SBL5.pro : MAAATVT-----VEEVRKAQRATGPATVLAIGTATPANCVHQADYPDYYFRITKSEHMTELKEKFKRMCDKSQIRKRYMHLTEEYLAENPNMCAYMAPSL : 95
P16107.pro : ----MANHHNAEIEEIRNRQRAQGPANILAIGTATPSNCVYQADYPDYYFRITNSEHMTDLKLKFKRMCEKSMIRKRYMHITEEYLKENPNVCAYEAPSL : 96
P48386.pro : ----MVT-----VEDIRRAQRAEGPATVMAIGTATPPNCVDQSTYPDYYFRITNSEHKAELKEKFKRMCDKSMIKKRYMYLTEEILKENPQVCEYMAPSL : 91
Q8RVK9.pro : ----MVT-----VEEFRKAQRAEGPATIMAIGTATPANCVLQSEYPDYYFRITNSEHKTELKEKFKRMCDKSMIRKRYMHLTEEILKENPNLCAYEAPSL : 91
TR11916|c0 : ----MVT-----VEEIRNAQRAKGPATVLAIGTATPSNCVMQADYPDYYFRITKSEHMTELKEKFKRMCDKSNIRKRYMHLNEEILKENPAMCEYMAPSL : 91
P48387.pro : ----MVT-----VEEVRRAQRAEGPATVMAIGTATPPNCVDQSTYPDYYFRITNSEHKTELKEKFQRMCDKSMIKKRYMYLTEEILKENPNVCAYMAPSL : 91
P22928.pro : ----MVT-----VEEIRRAQRAEGPATIMAIGTATPSNCVDQSTYPDYYFRITNSEHKTELKEKFQRMCDKSMIKKRYMHLTEEILKENPNICEYMAPSL : 91
P08894.pro : ----MVT-----VEEYRKAQRAEGPATVMAIGTATPTNCVDQSTYPDYYFRITNSEHKTDLKEKFKRMCEKSMIKKRYMHLTEEILKENPSMCEYMAPSL : 91
Q9FUB7.pro : ----MVT-----VEEVRKAQRAEGPATVMAIGTAVPPNCVDQATYPDYYFRITNSEHKAELKEKFQRMCDKSQIKKRYMYLNEEVLKENPNMCAYMAPSL : 91
B0LDU6.pro : ----MVT-----VDEVRKAQRAEGPATVLAIGTATPPNCIDQSTYPDYYFRITNSEHKTELKEKFQRMCDKSMIKKRYMYLTEEILKENPSMCEYMAPSL : 91
Q9AU11.pro : ----MVT-----VDEVRKAQRAEGPATILAIGTATPPNCVDQSTYPDYYFRITKSEHKTELKEKFQRMCDKSMIKKRYMYLTEEILKENPSMCEYMAPSL : 91
Q9ZRR8.pro : ----MVT-----VEEVRKAQRAEGPATVLAIGTATPPNCLDQSTYPDYYFRITNSEHKTELKEKFQRMCDKSMIKKRYMYLTEEILKEHPNMCAYMAPSL : 91
TR18393|c0 : ----MVT-----VEEVRKAQRAEGPATVMAIGTATPSNCVLQSEYPDYYFRITNSEHKVELKEKFKRMCDKSQIKKRYMHLTEEILKENPTLCEYMAPSL : 91
Q9ZS40.pro : ----MAN-HNAEIEEIRKRQRAQGPANILAIGTATPSNCVYQADYPDYYFRITNSEHMSDLKLKFKRMCEKSMIRKRYMHITEEYLKENPNVCAYEAPSL : 95
Q9FSB8.pro : --MAAVT-----VEEIRKAQRADGPAAVLAIGTATPANYVTQADYPDYYFRITKSEHMTELKEKFKRMCDKSMIRKRYMYLTEDILKENPNMCAYMAPSL : 93
Q9FSB9.pro : --MAAVT-----VEAIRKAQRADGPAAVLAIGTATPANYVTQADYPDYYFRITKSEHMTELKEKFKRMCDKSMIRKRYMHLTEDILKENPNMCAYMAPSL : 93
TR9672|c0_ : ----MVS-----VEEIRNAQRAKGPATVLAIGTSTPSNCVTQADYPDYYFRITKSEHMTDLKEKFKRMCDKSQIKKRYMHITEEILKENPSMCEYMAPSL : 91
 m 6 e R aQRa GPAt66AIGTatP nc6 Q YPDYYFRIT SeH LKeKF RMCdKS I 4RYM 6tEe Lk2nP 6C YmAPSL

 * 120 * 140 * 160 * 180 * 200
P51090.pro : DARQDMVVVEVPKLGKEAAAKAIKEWGQPKSKITHLVFCTTSGVDMPGADYQLTKLLGLKPSVKRLMMYQQGCFAGGTVLRLAKDLAENNAGSRVLVVCS : 191
P51075.pro : DARQTIVVVEVPKLGKEAATKAIKEWGQPKSKITHLVFCTTSGVDMPGADYQLTKLLGLRPSVKRLMMYQQGCFAGGTVLRLAKDLAENNKGARVLVVCS : 191
P48385.pro : DARQDVVVVEVPKLGKEAATKAIKEWGQPKSKITHLIFCTTSGVDMPGADYQLTKLLGLRPSVKRFMMYQQGCFAGGTVLRLAKDLAENNKGARVLVVCS : 194
Q9XJ57.pro : DARQDIVVVEVPKLGKEAATKAIKEWGQPKSKITHLIFCTTSGVDMPGADYQLTKLIGLRPSVKRFMMYQQGCFAGGTVLRLAKDLAENNKGARVLVVCS : 191
P06515.pro : DARQDIVVVEVPRLGKEAAQKAIKEWGQPKSKITHLVFCTTSGVDMPGADYQLTKLLGLRPSVKRFMMYQQGCFAGGTVLRMAKDLAENNAGARVLVVCS : 191
P48390.pro : DVRQDLVVVEVPKLGKEAAMKAIKEWGHPKSKITHLIFCTTSGVDMPGADYQLTKLLGLRPSVKRFMMYQQGCFAGGTVLRLAKDLAENNKGARVLVVCS : 194
Q9SBL5.pro : DARQDIVVVEVPKLGKAAAQKAIKEWGQPKSKITHLVFCTTSGVDMPGADYQLTKMLGLRPSVKRLMMYQQGCFAGGTVLRVAKDLAENNRGARVLVVCS : 195
P16107.pro : DARQDLVVVEVPRLGKEAASKAIKEWGQPKSKITHLIFCTTSGVDMPGADYQLTKLLGLRPSVKRFMMYQQGCFAGGTVLRLAKDLAENNAGARVLVVCS : 196
P48386.pro : DARQDMVVVEVPKLGKEAATKAIKEWGQPKSKITHLVFCTTSGVDMPGADYQLTKLLGLRPSVKRLMMYQQGCFAGGTVLRLAKDLAENNKGARVLVVCS : 191
Q8RVK9.pro : DARQDMVVVEVPKLGKEAATKAIKEWGQPKSKITHLVFCTTSGVDMPGADYQLTKLLGLRPSVKRLMMYQQGCFAGGTVLRLAKDLAENNKGARVLVVCS : 191
TR11916|c0 : DARQDMVVVEVPKLGKEAATKAIKEWGQPKSKITHLVFCTTSGVDMPGADYQLTKLLGLRPSVKRLMMYQQGCFAGGTVLRLAKDLAENNAGARVLVVCS : 191
P48387.pro : DARQDMVVVEVPKLGKEAATKAIKEWGQPKSKITHLVFCTTSGVDMPGADYQLTKLLGLRPSVKRLMMYQQGCFAGGTVLRLAKDLAENNKGARVLVVCS : 191
P22928.pro : DARQDIVVVEVPKLGKEAAQKAIKEWGQPKSKITHLVFCTTSGVDMPGADYQLTKLLGLRSSVKRLMMYQQGCFAGGTVLRLAKDLAENNKGARVLVVCS : 191
P08894.pro : DARQDIVVVEVPKLGKEAAQKAIKEWGQPKSKITHLVFCTTSGVDMPGCDYQLTKLLGLRPSVKRLMMYQQGCFAGGTVLRLAKDLAENNKGARVLVVCS : 191
Q9FUB7.pro : DARQDIVVVEVPKLGKEAAVKAIKEWGQPKSKITHLVFCTTSGVDMPGADYQLTKLLGLRPSVKRLMMYQQGCFAGGTVLRLAKDLAENNKGARVLVVCS : 191
B0LDU6.pro : DARQDMVVVEIPKLGKEAATKAIKEWGQPKSKITHLVFCTTSGVDMPGADYQLTKLLGLRPSVKRLMMYQQGCFAGGTVLRLAKDLAENNRGARVLVVCS : 191
Q9AU11.pro : DARQDMVVVEIPKLGKEAATKAIKEWGQPKSKITHLVFCTTSGVDMPGADYQLTKLLGLRPSVKRLMMYQQGCFAGGTVLRLAKDLAENNKGARVLVVCS : 191
Q9ZRR8.pro : DARQDMVVVEIPKLGKEAAVKAIKEWGQPKSKITHLVFCTTSGVDMPGADYQLTKLLGLRPSVKRLMMYQQGCFAGGTVLRLAKDLAENNRGARVLVVCS : 191
TR18393|c0 : DARQDMVVVEVPKLGKEAATKAIKEWGQSKSKITHLVFCTTSGVDMPGADYQLTKLLGLRPSVKRLMMYQQGCFAGGTVLRLAKDLAENNKGARVLVVCS : 191
Q9ZS40.pro : DARQDLVVVEVPRLGKEAAAKAIKEWGHPKSKITHLIFCTTSGVDMPGADYQLTKLLGLRPSVKRFMMYQQGCFAGGTVLRLAKDLAENNTGARVLVVCS : 195
Q9FSB8.pro : DARQDIVVVEVPKLGKEAAVKAIKEWGQPKSKITHLIFCTTSGVDMPGCDYQLTKLLGLRPSVKRFMMYQQGCFAGGTVLRLAKDLAENNRGARVLVVCS : 193
Q9FSB9.pro : DARQDIVVVEVPKLGKEAAVKAIKEWGQPKSKITHLIFCTTSGVDMPGCDYQLTKLLGLRPSVKRFMMYQQGCFAGGTVLRLAKDLAENNRGARVLVVCS : 193
TR9672|c0_ : DARQDMVVVEVPKLGKEAAAKAIKEWGQPKSKITHLVFCTTSGVDMPGADYQLTKLLGLRPSVKRLMMYQQGCFAGGTVLRLAKDLAENNAGARVLVVCS : 191
 DaRQd6VVVE6P4LGKeAA KAIKEWGqpKSKITHL6FCTTSGVDMPGaDYQLTK66GL4pSVKR MMYQQGCFAGGTVLR6AKDLAENN GaRVLVVCS

 * 220 * 240 * 260 * 280 * 300
P51090.pro : EITAVTFRGPSDTHLDSLVGQALFGDGAAAVIIGADPDTKIELPLFELVSAAQTILPDSEGAIDGHLREVGLTFHLLKDVPGLISKNIEKSLVEAFTPIG : 291
P51075.pro : EITAVTFRGPTDTHLDSLVGQALFGDGAAAVIVGADPDTSVERPLFELISAAQTILPDSDGAIDGHLREVGLTFHLLKDVPGIISKNIEKSLAEAFAPLG : 291
P48385.pro : EITAVTFRGPNDTHLDSLVGQALFGDGAAAVIVGADPDLTTERPLFEMISAAQTILPDSEGAIDGHLREVGLTFHLLKDVPGLISKNIEKALTQAFSPLG : 294
Q9XJ57.pro : EITAVTFRGPADTHLDSLVGQALFGDGAAAVIVGADPDTSVERPLYQLVSTSQTILPDSDGAIDGHLREVGLTFHLLKDVPGLISKNIEKSLSEAFAPLG : 291
P06515.pro : EITAVTFRGPADTHLDSLVGQALFGDGAAAVIVGSDPVVGVERPLFQIVTAAQTLLPDSHGAIDGHLREVGLTFHLLKDVPGLISKNIEKSLKEAFDPLG : 291
P48390.pro : EITAVTFRGPNDTHLDSLVGQALFGDGAAAVIVGSDPDLTTERPLFEMVSAAQTILPDSEGAIDGHLREVGLTFHLLKDVPGLISKNIEKALTTAFSPLG : 294
Q9SBL5.pro : EITAVTFRGPSESHLDSMVGQALFGDGAAAVIVGADPDERVERPLFQLVSASQTILPDSEGAIDGHLREVGLTFHLLKDVPGLISKNIERSLEEAFKPLG : 295
P16107.pro : EITAVTFRGPSDSHLDSLVGQALFGDGAAAVILGSDPDLSVERPLFQLISAAQTILPDSDGAIDGHLREVGLTFHLLKDVPGLISKNIEKSLKEAFGPIG : 296
P48386.pro : EITAVTFRGPSDTHLDSLVGQALFGDGAAAIIVGSDPIPEVEKPLFELVSAAQTILPDSDGAIDGHLREVGLTFHLLKDVPGLISKNIEKSLAEAFQPLG : 291
Q8RVK9.pro : EITAVTFRGPNDTHLDSLVGQALFGDGSAALIVGSDPIPEVEKPIFELVSAAQTILPDSDGAIDGHLREVGLTFHLLKDVPGLISKNIEKSLNEAFKPLG : 291
TR11916|c0 : EITAVTFRGPSDTHLDSLVGQALFGDGAAAVIVGADPDLKVERPLFELVSAGQTILPDSEGAIDGHLREVGLTFHLLKDVPGLISKNIEKSLNEAFSPIG : 291
P48387.pro : EITAVTFRGPSDAHLDSLVGQALFGDGAAAIIVGSDPIPEVEKPLFELVSAAQTILPDSDGAIDGHLREVGLTFHLLKDVPGLISKNIEKSLNEAFQPLN : 291
P22928.pro : EITAVTFRGPNDTHLDSLVGQALFGDGAAAIIIGSDPLPGVERPLFELVSASQTLLPDSEGAIDGHLREVGLTFHLLKDVPGLISKNIQKSLVEAFQPLG : 291
P08894.pro : EITAVTFRGPNDTHLDSLVGQALFGDGAGAIIIGSDPIPGVERPLFELVSAAQTLLPDSHGAIDGHLREVGLTFHLLKDVPGLISKNIEKSLEEAFKPLG : 291
Q9FUB7.pro : EITAVTFRGPTDTHLDSLVGQALFGDGAAAIIIGSDPIPEVEKPLFELVSAAQTILPDSEGAIDGHLREVGLTFHLLKDVPGLISKNVEKSLTEAFKPLG : 291
B0LDU6.pro : EIXAVTFRGPSDTHLDSLVGQALFGDGAAAIIVGADPLPKIERPLFELVSAAQTILPDSDGAIDGHLREVGLTFHLLKDVPGLISKNIEKSLNEAFKPLD : 291
Q9AU11.pro : EITAVTFRGPSDTHLDSLVGQALFGDGAAAIIVGSDPLPDIERPLFELVSAAQTILPDSDGAIDGHLREVGLTFHLLKDVPGLISKNIEKSLNEAFKPLD : 291
Q9ZRR8.pro : EITAVTFRGPSDTHLDSLVGQALFGDGAAAIIVGADPLPEVEKPLFEVVSTAQTILPDSDGAIDGHLREVGVTFHLLKDVPGLISKNIEKSLVEAFQPLG : 291
TR18393|c0 : EITAVTFRGPNDTHLDSLVGQALFGDGAAAIIIGSDPIEGVEKPLFEIVSAAQTILPDSDGAIDGHLREVGLTFHLLKDVPGLISKNIQKSLDEAFAPLG : 291
Q9ZS40.pro : EITAVTFRGPSDSHLDSLVGQALFGDGAAAVIVGSDPDLSVERPLFQLISAAQTILPDSDGAIDGHLREVGLTFHLLKDVPGLISKNIEKSLKEAFGPIG : 295
Q9FSB8.pro : EITAVTFRGPADTHLDSLVGQALFGDGAAAVIVGADPNESIERPLYQLVSAAQTILPDSDGAIDGHLREVGLTFHLLKDVPGLISKNIEKSLKEAFGPIG : 293
Q9FSB9.pro : EITAVTFRGPADTHLDSLVGQALFGDGAAAVIVGADPDESIERPLYQLVSAAQTILPDSDGAIDGHLREVGLTFHLLKDVPGLISKNIEKSLKEAFGPIG : 293
TR9672|c0_ : EITAVTFRGPSDTHLDSLVGQALFGDGAAAVILGADPDLSVERPLFELVSAAQTILPDSDGAIDGHLREVGLTFHLLKDVPGLISKNIEKSLVEAFTPIG : 291
 EItAVTFRGP d HLDS6VGQALFGDGaaA6I6G DP E P652663aaQT6LPDS GAIDGHLREVG6TFHLLKDVPG6ISKN624sL AF P6g

 * 320 * 340 * 360 * 380 * 400
P51090.pro : ISDWNSLFWIAHPGGPAILDQVELKLGLKEEKLRATRHVLSEYGNMSSACVLFILDEMRKKSIEEGKGSTGEGLEWGVLFGFGPGLTVETVVLHSVSAPA : 391
P51075.pro : ISDWNSLFWIAHPGGPAILDQVESKLGLKEEKLRATRHVLSEYGNMSSACVLFILDEMRRNSLEGGKVTTGEGLEWGVLFGFGPGLTVETVVLHSVPVPV : 391
P48385.pro : ITDWNSIFWIAHPGGPAILDQVELKLGLKEEKMRATRHVLSEYGNMSSACVLFIIDEMRKKSAEDGAATTGEGLDWGVLFGFGPGLTVETVVLHSLPTTM : 394
Q9XJ57.pro : ISDWNSIFWIAHPGGPAILDQVESKLGLKGEKLKATRQVLSEYGNMSSACVLFILDEMRKKSVEEAKATTGEGLDWGVLFGFGPGLTVETVVLHSVPIKA : 391
P06515.pro : ISDWNSVFWIAHPGGPAILDQVEEKLGLKPEKLRSTRQVLSEYGNMSSACVLFILDEMRKSSAKEGMSTTGEGLDWGVLFGFGPGLTVETVVLHSVPLN- : 390
P48390.pro : INDWNSIFWIAHPGGPAILDQVELKLGLKEEKLRATRHVLSEYGNMSSACVLFIIDEMRKKSSENGAGTTGEGLEWGVLFGFGPGLTVETVVLHSVPTTV : 394
Q9SBL5.pro : ITDYNSIFWVAHPGGPAILDQVEAKVGLKKERMRATRHVLSEYGNMSSACVLFILDEMRKRSAEDGQATTGEGLDWGVLFGFGPGLTVETVVLHSVPITT : 395
P16107.pro : ISDWNSLFWIAHPGGPAILDQVELKLGLKEEKMRATRQVLSDYGNMSSACVLFILDEMRKKSIEEGKATTGEGLDWGVLFGFGPGLTVETVVLHSVPATF : 396
P48386.pro : ISDWNSLFWIAHPGGPAILDQVELKLGLKEEKLRATRHVLSEYGNMSSACVLFILDEMRKKSAADGLKTTGEGLEWGVLFGFGPGLTVETVVLHSLST-- : 389
Q8RVK9.pro : ISDWNSLFWIAHPGGPAILDQVESKLALKTEKLRATRHVLSEYGNMSSACVLFILDEMRRKCVEDGLNTTGEGLEWGVLFGFGPGLTVETVVLHSVAI-- : 389
TR11916|c0 : ISDWNSLFWIAHPGGPAILDQVEIKLRLKEEKLKSTRHVLSEYGNMSSACVLFILDDMRKKSLEQGKSTYGEGLEWGVLFGFGPGLTVETVVLHSVPTVA : 391
P48387.pro : ITDWNSLFWIAHPGGPAILDQVELKLALKPEKLRATRHVLSEYGNMSSACVLFILDEMRKSSAKKGLKTTGEGLDWGVLFGFGPGLTVETVVLHSVST-- : 389
P22928.pro : ISDWNSIFWIAHPGGPAILDQVELKLGLKPEKLRATRHVLSEYGNMSSACVLFILDEMRKASSKEGLGTTGEGLEWGVLFGFGPGLTVETVVLHSVST-- : 389
P08894.pro : ISDWNSLFWIAHPGGPAILDQVEIKLGLKPEKLKATRNVLSDYGNMSSACVLFILDEMRKASAKEGLGTTGEGLEWGVLFGFGPGLTVETVVLHSVAT-- : 389
Q9FUB7.pro : ISDWNSLFWIAHPGGPAILDQVEAKLSLKPEKLRATRHVLSEYGNMSSACVLFILDEMRRKSKEDGLKTTGEGIEWGVLFGFGPGLTVETVVLHSVAIN- : 390
B0LDU6.pro : ITDWNSLFWIAHPGGPAILDQVETKLGLKPEKLEATRHILSEYGNMSSACVLFILDEVRRKSATNGLKTTGEGLEWGVLFGFGPGLTVETVVLHSVGVTA : 391
Q9AU11.pro : ITDWNSLFWIAHPGGPAILDQVEAKLGLKPEKLEATRNILSEYGNMSSACVLFILDEVRRKSVANGHKTTGEGLEWGVLFGFGPGLTVETVVLHSVAAST : 391
Q9ZRR8.pro : ISDWNSLFWIAHPGGPAILDQVEEKLALKPEKLGATRHVLSEYGNMSSACVLFILDEMRRKSAEKGLKTTGEGLDWGVLFGFGPGLTVETVVLHSLTT-- : 389
TR18393|c0 : ITDWNSLFWIAHPGGPAILDQVEEKLHLKPEKLRATRHVLSEYGNMSSACVLFIMDEMRKSSAKQGLKTTGEGIEWGVLFGFGPGLTVETVVLHSVAA-- : 389
Q9ZS40.pro : ISDWNSLFWIAHPGGPAILDQVELKLGLKEEKMRATRQVLSDYGNMSSACVLFILDEMRKKSIEEGKATTGDGLDWGVLFGFGPGLTVETVVLHSVPATI : 395
Q9FSB8.pro : ISDWNSIFWIAHPGGPAILDQVEAKLGLKEEKLRATRQVLSEYGNMSSACVLFILDEMRKKCAEEGRATTGEGLDWGVLFGFGPGLTVETVVLRSVPINA : 393
Q9FSB9.pro : ISDWNSIFWIAHPGGPAILDQVEAKLGLKEEKLRATRQVLSEYGNMSSACVLFILDEMRKNCAEEGRATTGEGLDWGVLFGFGPGLTVETVVLRSVPIKA : 393
TR9672|c0_ : ITDWNSLFWIAHPGGPAILDQVELKLGLKEEKLRATRHVLSEYGNMSSACVLFILDEMRRKSEEEGKPTFGEGLEWGVLFGFGPGLTVETVVLHSVAPVA : 391
 I D5NS6FW6AHPGGPAILDQVE K6 LK E46 aTR 6LSeYGNMSSACVLFI6De6R4 s g 3tGeG6 WGVLFGFGPGLTVETVVLhS6


P51090.pro : AH---- : 393
P51075.pro : EASH-- : 395
P48385.pro : AIAT-- : 398
Q9XJ57.pro : ------ : -
P06515.pro : ------ : -
P48390.pro : TVAV-- : 398
Q9SBL5.pro : GAAITA : 401
P16107.pro : TH---- : 398
P48386.pro : ------ : -
Q8RVK9.pro : ------ : -
TR11916|c0 : TQ---- : 393
P48387.pro : ------ : -
P22928.pro : ------ : -
P08894.pro : ------ : -
Q9FUB7.pro : ------ : -
B0LDU6.pro : ------ : -
Q9AU11.pro : ------ : -
Q9ZRR8.pro : ------ : -
TR18393|c0 : ------ : -
Q9ZS40.pro : TH---- : 397
Q9FSB8.pro : ------ : -
Q9FSB9.pro : ------ : -
TR9672|c0_ : AK---- : 393


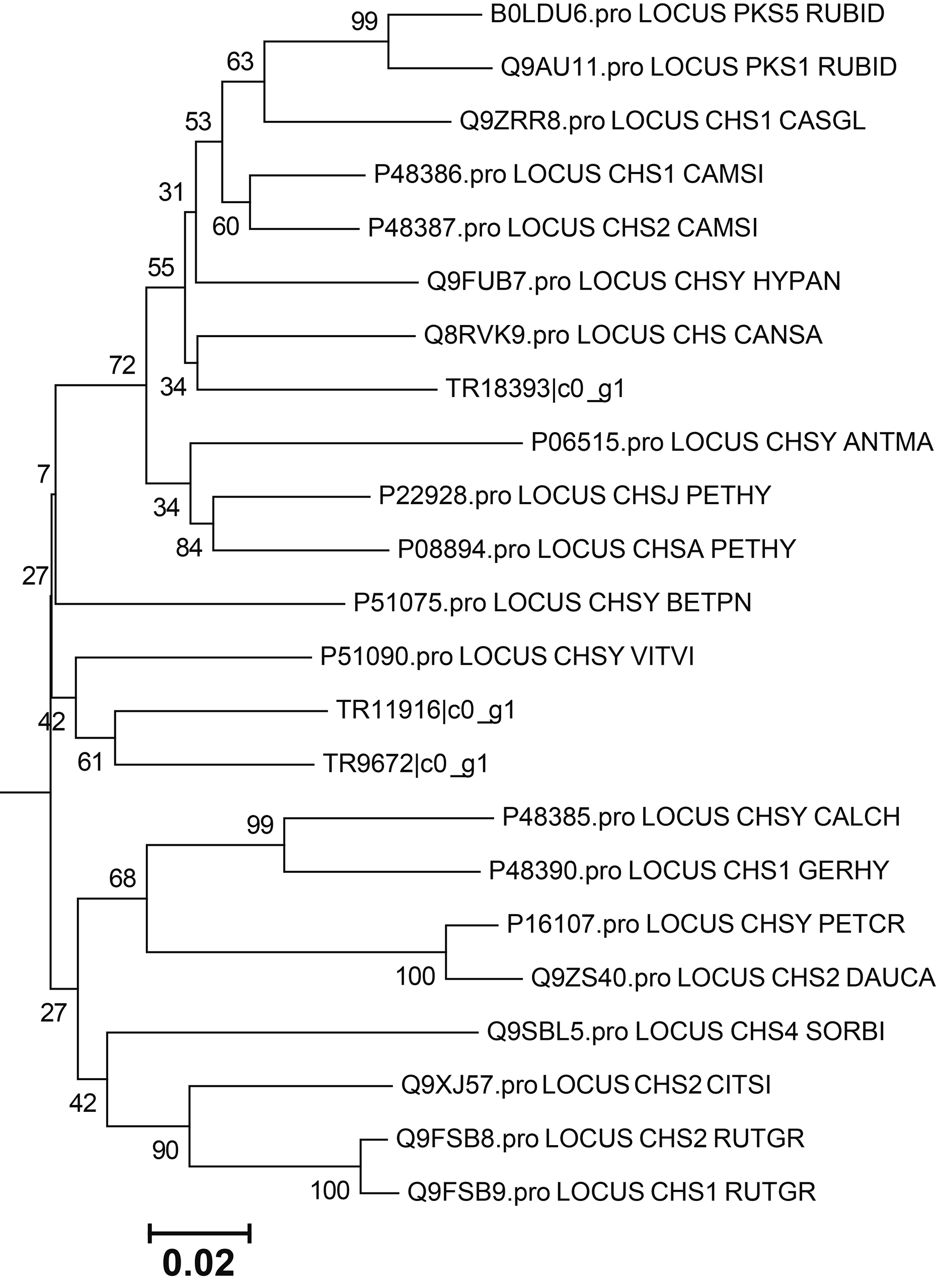

Supplement: S7 Fig — (DOCX) [file pone.0182348.s021.docx]
