# Supplementary material for: Understanding the molecular mechanisms underlying the effects of light intensity on flavonoid production by RNA-seq analysis in Epimedium pseudowushanense B.L.Guo
Source: PLoS One. 2017 Aug 7;12(8):e0182348. doi: 10.1371/journal.pone.0182348 (PMC5546586; doi:10.1371/journal.pone.0182348)

**S8 Fig. Sequence alignment of chalcone isomerase (CHI) proteins from *E. pseudowushanense* and various other plants, and phylogenetic relationships of chalcone isomerase (CHI) proteins from *E. pseudowushanense* and various other plants.**

* 20 * 40 * 60 * 80 * 100
Q45QI7.pro : MSPSQSPSVAQVQIESHVFPPT-VKPPGTSKPFFLGGAGERGLEIQGKFIKFTAIGVYLEDSAIPSLAVKWKGKTAEELTDSVDFFRDIVSGPFEKFTQV : 99
A5HBK6.pro : MAPP--PSLAGLQVEATAFPPS-VKPPGSSNTLFLGGAGVRGLEIQGNFVKFTAIGVYLEDNAVPLLAVKWKGKTAEELSESVEFFRDIVTGPFEKFIQV : 97
Q9ZWR1.pro : MNPS--PSVTELQVENVTFTPS-VQPPGSTKSHFLGGAGERGLEIEGKFVKFTAIGVYLEDDAVPLLAGKWKGKTAEELTESVEFFRDVVTGPFEKFMKV : 97
P51117.pro : MSQV--PSVTAVQVENVLFPPS-VKPPGSTNDLFLGGAGVRGLEIQGKFVKFTAIGVYLENSAVPTLAVKWKGKTVEELADSVDFFRDVVTGPFEKFTKV : 97
A5ANT9.pro : MSPV--PSVTAVQVENVLFPPS-VKPPGSTNDLFLGGAGVRGLEIQGKFVKFTAIGVYLESSAVPTLAVKWKGKTVEELADSVDFFRDVVTGPFEKFTKV : 97
Q4AE11.pro : MAQ----SVTGIQIGGMSFPPS-VKPPGSGNTFFLGGAGVRGMEIQGNFVKFTAIGVYLEDKAVPALAVKWKGKTAEELTESVEFFREIVTGPFEKFTQV : 95
Q4AE12.pro : MAQ----SVTGIQIGGTSFPPA-VKPPGSGNTLFLGGAGVRGMEIQGNFVKFTAIGVYLEDKAVPALSVKWKGKTAEELTESVEFFREIVTGPFEKFTQV : 95
O65333.pro : MAPFT-KSVTEVQVESVIFPPE-VKPPGSSKTLFLGGAGVRGIEIQGKFIKFTAIGVYLEDNAVPSLAVKWKGKSAQELTESVEFFRDIVTGPMEKFTRV : 98
Q8H0G1.pro : -MAL--PSVTALQVENVAFPPTLIKPPASANTLFLGGAGERGLHIQDKFVKFTAIGIYLQDTAVPSLAVKWKGKPVDELTESVQFFRDIVTGPFEKFMQV : 97
A2IBF8.pro : MSTS--LSVTELQVENFTFPPT-VKPPGSTKTLFLGGAGERGLEIQGKFIKFTAIGVYLEDSAVNCLGVKWKGKSAVELTESVEFFRDVVTGDFEKFIRV : 97
TR19370|c0 : MAK------SSLQVEGFTFSPS-LKPPGSSNTLFLGGAGERGLQIQDKFIKFTAIGVYLEEAALPLLAEKWKGKSAEELTDSVEFFSDIVTGPYEKFTII : 93
 m s 6Q6e FpP 6kPPg3 FLGGAG RG6 I2g F6KFTAIG6YL2 A6p LavKWKGK EL SV FFrd6V3Gp EKF 6

 * 120 * 140 * 160 * 180 * 200
Q45QI7.pro : TMILPLTGQQYSEKVTENCVAYWKAVGTYTDAEAKAIEKFIEVFKDETFPPGGSILFTQSPLGSLTIAFSKDGSLPETGTVVMENKQLSEAVLESIIGKH : 199
A5HBK6.pro : TTILPLTGQQYSEKVSENCVAFWKSVGIYTDAEGKAIEKFIEVFKDQNFPPGASILFTQSPKGSLTICFSKDASVPEAANAVIENKLLSEAVLESILGKH : 197
Q9ZWR1.pro : TMILPLTGAQYSEKVAENCIAIWKFFGIYTDAEAKAIEKFTEVFKDEIFPPGSSILFTQS-PGSLTISFSKDGSIPKDGVAVIESNLLSEAVLESMIGKN : 196
P51117.pro : TTILPLTGRQYSDKVSENCVAFWKSVGIYTDAEAKAIEKFNEVLKDETFPPGNSILFTHSPLGALTMSFSKDGSLPEVGNAVIENKLLTEAVLESIIGKH : 197
A5ANT9.pro : TTILPLTGRQYSDKVSENCVAFWKSVGIYTDAEAKAIEKFNEVLKDETFPPGNSILFTHSPLGALTMSFSKDGSLPEVGNAVIENKLLTEAVLESIIGKH : 197
Q4AE11.pro : TMILPLTGQQYSEKVSENCVAIWKKFGIYTDAEAKAIEKFIEVFKDQTFPPGASILFTQSPDGSLTIGFSKDGCIPEVGNAVIENKLLSESVLESIIGKP : 195
Q4AE12.pro : TMILPLTGQQYSEKVSENCVAIWKKFGIYTDAEAKAIEKFIEVFKDQTFPPGASILFTQSPNGSLMIGISKDGSIPEVGNAVIENKLLSESVLESIIGKQ : 195
O65333.pro : TTILPLTGQQYSEKVSENCVAAWKSLGIYSDAEAKAIEKFIEIFKDQTFPPAASNLFTQSPLGSLTMSFSKDGSIPEVGNAVLENKLLSEAVLESIIGKH : 198
Q8H0G1.pro : TMILPLTGQQYSEKVSENCVAIWKHLGIYTDEEGKAIDKFVSVFKDQTFPPGSSILFTVLPKGSLAISFSKDGSIPEVESAVIDNKLLSEAVLESMIGAH : 197
A2IBF8.pro : TMILPLTGQQYSEKVSENCVAIWKSLGIYTDAEAKAIEKFIEVFKDENFPPGSSILFTISGQGSLTIGFSKDSSVPEGGKVVIENKLLANSVLESVIGKN : 197
TR19370|c0 : TMILPLTGQQYAEKVIENCVANWKAIGIYNEAQEKAAEKFQEVFKDETFPPGTSILFTQSPKGTLTIAFSKDGSIPEEGVAVIENTPLSRAVLYSIIGEH : 193
 T ILPLTG QYseKV ENC6A WK GiY da2 KAieKF e6fKD2 FPPg SiLFT sp G Lt6 fSKDgs6Pe g aV6enklL e VLeS66Gk

 * 220 * 240 *
Q45QI7.pro : GVSPAAKKSLAARMSELLKE-------KPEAQTAAAAE-------------------- : 230
A5HBK6.pro : GVSPAAKRSLAARLSELLN----------G--------CK------------------ : 219
Q9ZWR1.pro : GVSPAAKKSLAERLSALLN---------VTSDKMK----------------------- : 222
P51117.pro : GVSPEAKKSLAARLSELFCK-------EAGDEKIEAEKVAPVAC-------------- : 234
A5ANT9.pro : GVSPEAKKSLAARLSELFCK-------EAGDEKIEAEKVAPVAC-------------- : 234
Q4AE11.pro : GVSPEARKSVATRLSELLKE---SDHCVAGNGKVD--ECTKEAEVKA----------- : 237
Q4AE12.pro : GVSPEARKSVATRLSELLKE---SDHCVAGNEKVD--ECTKEAENKA----------- : 237
O65333.pro : GVSPEAKQNLATRLVQLLNENSTTDLNESENEKLNSNEVSKEEKPLQVEKSAFKEVEV : 256
Q8H0G1.pro : GVSPAAKQSLASRLSELFKH-------HAEV--------------------------- : 221
A2IBF8.pro : GVSPAAKESLASRLSPLFNDC------GADSEKPQS---------------------- : 227
TR19370|c0 : GVSPVAKKSLASRISELLV--------------------------------------- : 212
 GVSP A4 s6A R6s L


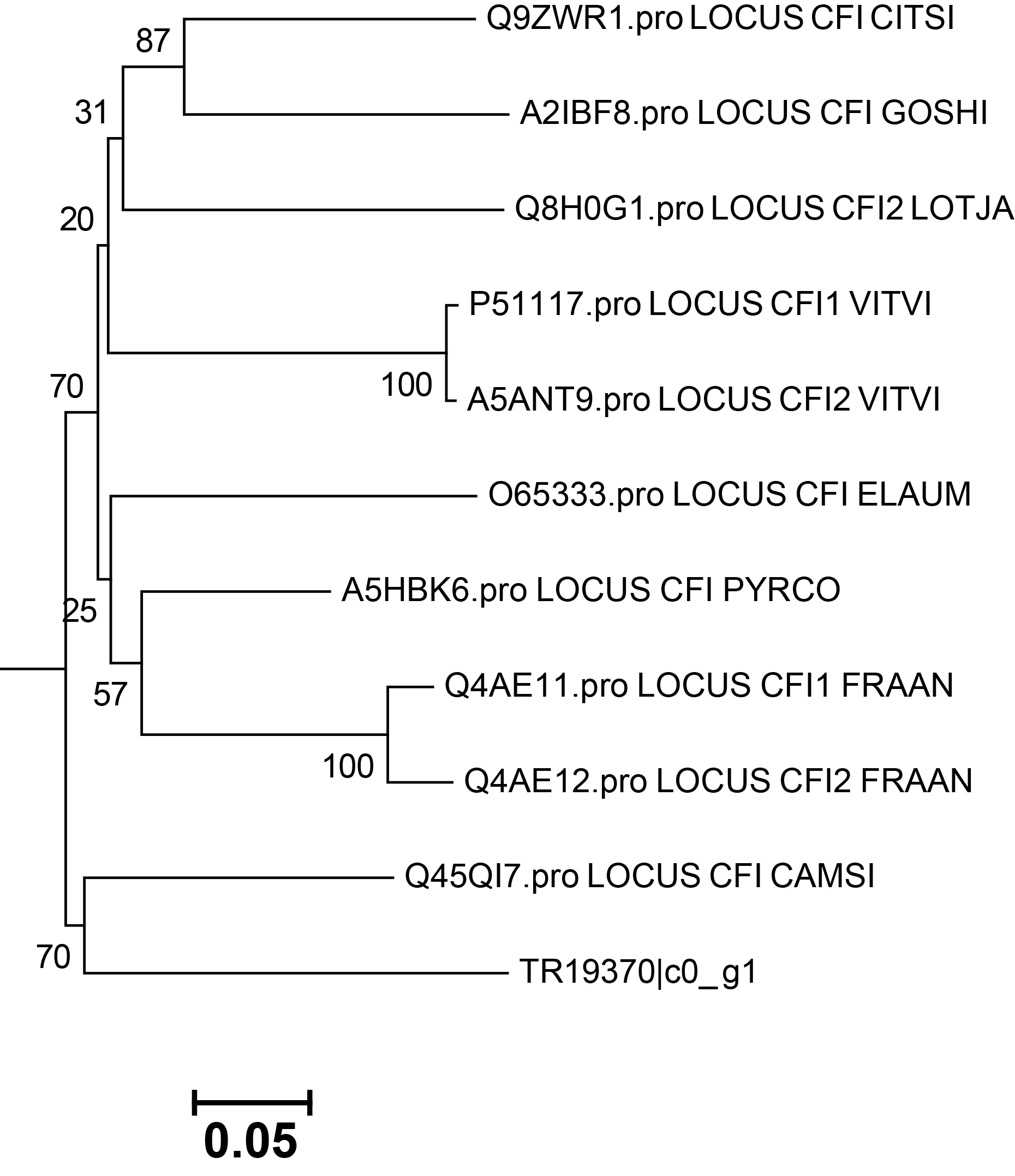

Supplement: S8 Fig — (DOCX) [file pone.0182348.s022.docx]
