# Supplementary material for: Understanding the molecular mechanisms underlying the effects of light intensity on flavonoid production by RNA-seq analysis in Epimedium pseudowushanense B.L.Guo
Source: PLoS One. 2017 Aug 7;12(8):e0182348. doi: 10.1371/journal.pone.0182348 (PMC5546586; doi:10.1371/journal.pone.0182348)

**S9 Fig. Sequence alignment of leucoanthocyanidin dioxygenase proteins from *E. pseudowushanense* and various other plants, and phylogenetic relationships of leucoanthocyanidin dioxygenase proteins from *E. pseudowushanense* and various other plants.**

* 20 * 40 * 60 * 80 * 100
P51091.pro : MVSS--DS-VNSRVETLAGSGISTIPKEYIRPKDELVN-IGDIFEQEKN----NEGPQVPTIDLKEIESDN----EKVRAKCREKLKKAAVDWGVMHLVN : 88
P51092.pro : MVNA--VVTTPSRVESLAKSGIQAIPKEYVRPQEELNG-IGNIFEEEKK----DEGPQVPTIDLKEIDSED----KEIREKCH-QLKKAAMEWGVMHLVN : 88
Q96323.pro : MVAV--ER-----VESLAKSGIISIPKEYIRPKEELES-INDVFLEEKK----EDGPQVPTIDLKNIESDD----EKIRENCIEELKKASLDWGVMHLIN : 84
O04274.pro : MVTS--AMGPSPRVEELARSGLDTIPKDYVRPEEELKSIIGNILAEEKS----SEGPQLPTIDLEEMDSRD----EEGRKKCHEELKKAATDWGVMHLIN : 90
P51093.pro : MVTS--VA---PRVESLSSSGIQSIPKEYIRPQEELTS-IGNVFEEEKK----DEGPQVPTIDLKDIESEDEVVRREIRERCREELKKAAMEWGVMHLVN : 90
P41213.pro : MESSPLLQLPAARVEALSLSGLSAIPPEYVRPADERAG-LGDAFDLARTHANDHTAPRIPVVDISPFLDSSS--QQQQRDECVEAVRAAAADWGVMHIAG : 97
TR9962|c0_ : MAPT--IATAGTRVESLSSSGMTFIPKEYVRPQEELDS-IGDVFEEERRY---EEGPQIPIIDLKGLNSED----KEIRERCREELNKAAMDWGVMHIVN : 90
 M rVE L SG6 IPkeY6RP El 6g1 f e4 gPq6P 6D6 s R C e 6 kAa WGVMH6 n

 * 120 * 140 * 160 * 180 * 200
P51091.pro : HGISDELMDKVRKAGKAFFDLPIEQKEKYANDQASGKIQGYGSKLANNASGQLEWEDYFFHCVYPEDKRDLSIWPQTPADYIEATAEYAKQLRELATKVL : 188
P51092.pro : HGISDELINRVKVAGETFFDQPVEEKEKYANDQANGNVQGYGSKLANSACGQLEWEDYFFHCAFPEDKRDLSIWPKNPTDYTPATSEYAKQIRALATKIL : 188
Q96323.pro : HGIPADLMERVKKAGEEFFSLSVEEKEKYANDQATGKIQGYGSKLANNASGQLEWEDYFFHLAYPEEKRDLSIWPKTPSDYIEATSEYAKCLRLLATKVF : 184
O04274.pro : HGIPEELIDRVKAAGKEFFELPVEEKEAYANDQAAGNVQGYGSKLANNASGQLEWEDYFFHCVYPEHKTDLSIWPTKPPDYIPATSEYAKQLRALATKIL : 190
P51093.pro : HGISDDLINRVKVAGETFFNLPMEEKEKYANDQASGKIAGYGSKLANNASGQLEWEDYFFHLIFPEDKRDMTIWPKTPSDYVPATCEYSVKLRSLATKIL : 190
P41213.pro : HGIPAELMDRLRAAGTAFFALPVQDKEAYANDPAAGRLQGYGSRLATNTCGQREWEDYLFHLVHPDGLADHALWPAYPPDYIAATRDFGRRTRDLASTLL : 197
TR9962|c0_ : HGIPEELLNRLRVAGKAFFDLPIEEKEKHANDQVTGQIQGYGSKLANNASGQLEWEDYFFHLIFPEDKRDMSIWPKNPSDYMEATSEYATRLRSLATDVL : 190
 HGI L6 464 AG FF lp62 KE yANDqa G 6qGYGS4LAnna GQlEWEDYfFH Pe k D 6WP P DY AT e5 R LA3 6l

 * 220 * 240 * 260 * 280 * 300
P51091.pro : KVLSLG-LGLDEG-RLEKEVG-------GLEELLLQMKINYYPKCPQPELALGVEAHTDVSALTFILHNMVPGLQLFYEGKWVTAKCVPNSIVMHIGDTL : 279
P51092.pro : TVLSIG-LGLEEG-RLEKEVG-------GMEDLLLQMKINYYPKCPQPELALGVEAHTDVSALTFILHNMVPGLQLFYEGQWVTAKCVPNSIIMHIGDTI : 279
Q96323.pro : KALSVG-LGLEPD-RLEKEVG-------GLEELLLQMKINYYPKCPQPELALGVEAHTDVSALTFILHNMVPGLQLFYEGKWVTAKCVPDSIVMHIGDTL : 275
O04274.pro : SVLSIG-LGLEKG-RLEKEVG-------GAEDLIVQMKINFYPKCPQPELALGWEAHTDVSALTFILHNMVPGLQLFYEDKWVTAKCVPNSIIMHIGDTL : 281
P51093.pro : SVLSLG-LGLEEG-RLEKEVG-------GMEELLLQKKINYYPKCPQPELALGVEAHTDVSALTFILHNMVPGLQLFYEGKWVTAKCVPNSIIMHIGDTI : 281
P41213.pro : AILSMGLLGTDRGDALEKALTTTTTRTAADDDLLLQLKINYYPRCPQPELAVGVEAHTDVSALSFILHNGVPGLQVLHGARWVTARHEPGTIIVHVGDAL : 297
TR9962|c0_ : SALSLA-LGLEEN-RLETEVG-------GMEELLLQLKINYYPRCPQPDLALGVEAHTDISALTFLFTNMVPGLQVFYGGKWVTAKCVEDSIVMHIGDTV : 281
 LS6g LGl rLEke6g g e L66Q KIN5YP4CPQPeLA6GvEAHTD6SAL3F6lhNmVPGLQ6fy WVTA4cvp 3I66H6GDt6

 * 320 * 340 * 360 * 380 * 400
P51091.pro : EILSNGKYKSILHRGMVNKEKVRISWAVFCEPPKEKIILKPLPETVSE-DEPAMFPPRTFAEHIQHKLFRKSQE-----------A---LLPK------- : 357
P51092.pro : EILSNGKYKSILHRGVVNKEKVRFSWAIFCEPPKEKIILKPLPETVTE-AEPPRFPPRTFAQHMAHKLFRKDDKDAAVEHKVFNEDELDTAAEHKVLKKD : 378
Q96323.pro : EILSNGKYKSILHRGLVNKEKVRISWAVFCEPPKDKIVLKPLPEMVSV-ESPAKFPPRTFAQHIEHKLFGKEQE-----------ELVSEKND------- : 356
O04274.pro : EILSNGKYKSILHRGLVNKEKVRISWAVFCEPPKEKIVLQPLPETVSE-VEPPRFPPRTFAQHLKHKLFRKTDG-----------D-LDEKPTY------ : 362
P51093.pro : EILSNGKYKSILHRGLVNKEKVRISWAVFCEPPKEKIILKAHCQRRCLRLSHHSSHLAPFPNIFSTSSSGRPRR-------------LYSPNEL------ : 362
P41213.pro : EILSNGRYTSVLHRGLVNREAVRISWVVFCEPPPDSVLLHPLPELVTE-GHPARFTPRTFKQHLDRKLFKKKQQ-----------HKAKAEEEDGGNGDH : 385
TR9962|c0_ : EILSNGKYKSILHRGLVNKEKVRISWAVFCEPPKDKIVLKPLEELVTD-SEPARFPPRTFAQHIEHKLFKKTQA------------ALLSKE-------- : 360
 EILSNG4YkS6LHRG6VN4EkVRiSWa6FCEPPk k66L pl 2 v p f prtF h klf 4

 * 420 * 440 *
P51091.pro : ---------------------------------------------------- : -
P51092.pro : NQDAVAENKDIKEDEQCGPAEHKDIKEDGQGAAAENKVFKENNQDVAAEESK : 430
Q96323.pro : ---------------------------------------------------- : -
O04274.pro : ---------------------------------------------------- : -
P51093.pro : ---------------------------------------------------- : -
P41213.pro : HRHEPPPQTN------------------------------------------ : 395
TR9962|c0_ : ---------------------------------------------------- : -


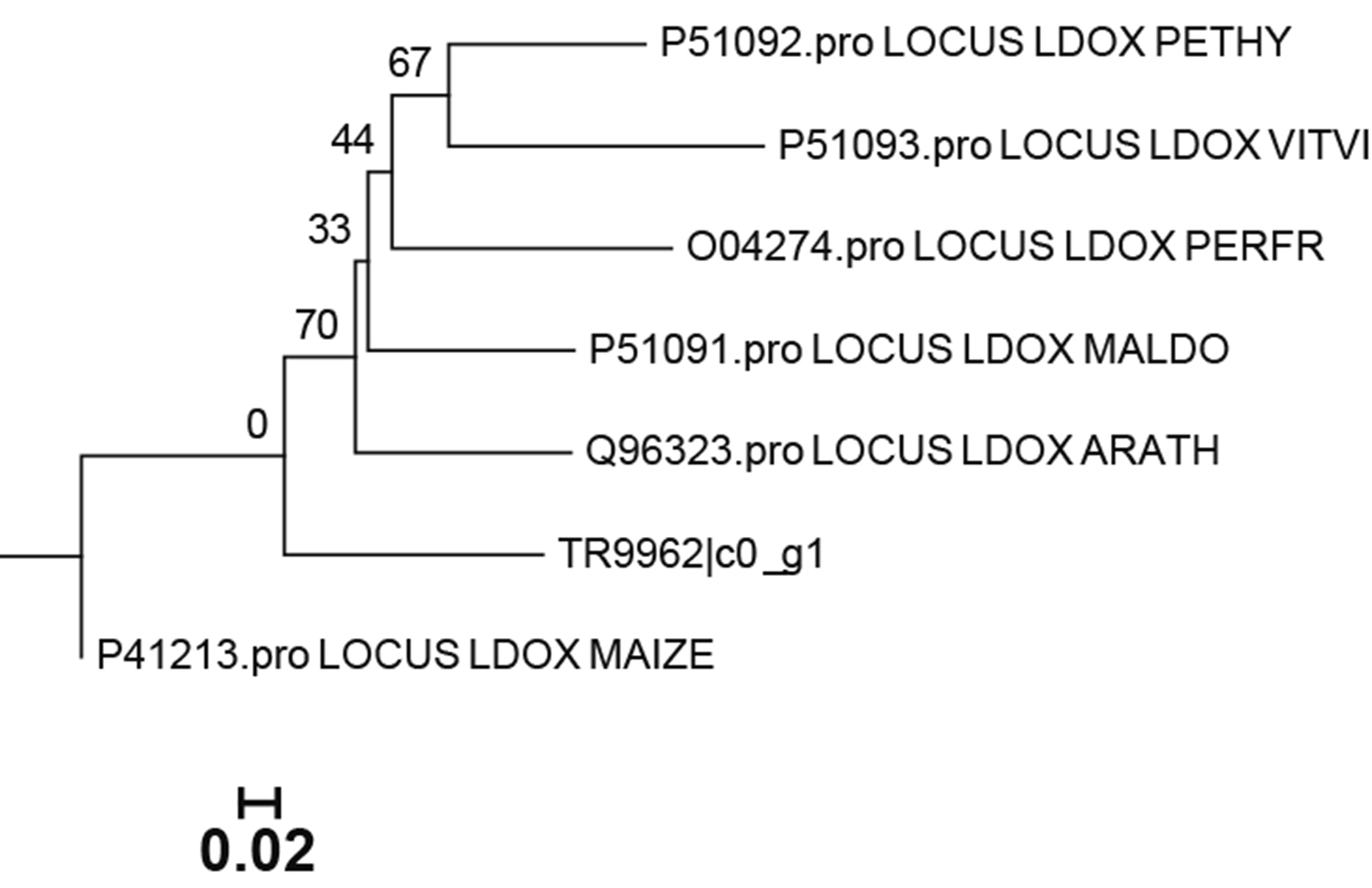

Supplement: S9 Fig — (DOCX) [file pone.0182348.s023.docx]
