# Supplementary material for: Understanding the molecular mechanisms underlying the effects of light intensity on flavonoid production by RNA-seq analysis in Epimedium pseudowushanense B.L.Guo
Source: PLoS One. 2017 Aug 7;12(8):e0182348. doi: 10.1371/journal.pone.0182348 (PMC5546586; doi:10.1371/journal.pone.0182348)

**S10 Fig. Sequence alignment of flavonol synthase (FLS) proteins from *E. pseudowushanense* and various other plants, and phylogenetic relationships of flavonol synthase (FLS) proteins from *E. pseudowushanense* and various other plants.**

* 20 * 40 * 60 * 80 * 100
Q9ZWQ9.pro : --------------MEVE-RVQAIASLSHSNGTIPAEFIRPEKEQPASTTYHGPAPEIPTIDLD--DPVQDRLVRSIAEASREWGIFQVTNHGIPSDLIC : 83
Q9M547.pro : --------------MEVQ-RVQEIASLSKVIDTIPAEYIRSENEQPVISTVHGVVLEVPVIDLS--DSDEKKIVGLVSEASKEWGIFQVVNHGIPNEVIR : 83
Q07512.pro : MKTAQGVSAT--LTMEVA-RVQAIASLSKCMDTIPSEYIRSENEQPAATTLHGVVLQVPVIDLR--DPDENKMVKLIADASKEWGIFQLINHGIPDEAIA : 95
Q7XZQ6.pro : --------------MEVE-RVQAISKMSRCMDTIPSEYIRSESEQPAVTTMQGVVLQVPVIDLGSSNNTEENLVELIAEASREWGIFQVVNHGIPDDAIA : 85
Q41452.pro : MKTIQGQSATTALTMEVA-RVQAISSITKCMDTIPSEYIRSENEQPAATTLQGVVLEVPVIDISNVDDDEEKLVKEIVEASKEWGIFQVINHGIPDEVIE : 99
Q96330.pro : --------------MEVE-RVQDISSSSLLTEAIPLEFIRSEKEQPAITTFRGPTPAIPVVDLS--DPDEESVRRAVVKASEEWGLFQVVNHGIPTELIR : 83
Q9XHG2.pro : --------------MGVE-SVERERESNE--GTIPAEFIRSENEQPGITTVHGKVLEVPIIDFS--DPDEEKLIVQITEASSNWGMYQIVNHDIPSEVIS : 81
Q9FFQ5.pro : --------------MEME-KNQHISS-----------------------------LDIPVIDLS--NPDEELVASAVVKASQEWGIFQVVNHGIPTELIL : 54
O04395.pro : --------------------------------------------------------QVPVVDLS--CPDEELVARTVVKASEDWGVFQVVNHGIPTELIQ : 42
Q9FFQ4.pro : --------------MEEE-RDHNASESS-----LP-----SLSKQLESSTLGGSAVDVPVVDLS--VSDEDFLVREVVKASEEWGVFQVVNHGIPTELMR : 73
TR6321|c0_ : -------------MAEVQTRVQAIANMSSIIDTIPAEFIRSEKEQPALTTFTGPTPEIPTIDLS--DPDQDNLVRVIADASREWGLFQIVNHGLPVEAIK : 85
 me p q t g 6P 6D d2 6 6 AS eWG65Q6 NHg6P e 6

 * 120 * 140 * 160 * 180 * 200
Q9ZWQ9.pro : KLQAVGKEFFE-LPQEEKEVYSRPADAKDVQGYGTKLQKEVEG----KKSWVDHLFHRVWPPSSINYRFWPKNPPSYRAVNEEYAKYMREVVDKLFTYLS : 178
Q9M547.pro : KLQEVGKHFFE-LPQEEKELIAKPEGSQSIEGYGTRLQKEVDG----KKGWVDHLFHKIWPPSAINYQFWPKNPPAYREANEEYAKRLQLVVDNLFKYLS : 178
Q07512.pro : DLQKVGKEFFEHVPQEEKELIAKTPGSNDIEGYGTSLQKEVEG----KKGWVDHLFHKIWPPSAVNYRYWPKNPPSYREANEEYGKRMREVVDRIFKSLS : 191
Q7XZQ6.pro : KLQKVGKEFFE-LPQQEKEVIAKPEGYQGVEGYGTKLQKELGG----KKGWVDHLFHIIWPKSAVNYNFWPNNPPLYREANEEYAVALRGVVDKLFEALS : 180
Q41452.pro : NLQKVGKEFFEEVPQEEKELIAKKPGAQSLEGYGTSLQKEIEG----KKGWVDHLFHKIWPPSAINYRYWPKNPPSYREANEEYAKWLRKVADGIFRSLS : 195
Q96330.pro : RLQDVGRKFFE-LPSSEKESVAKPEDSKDIEGYGTKLQKDPEG----KKAWVDHLFHRIWPPSCVNYRFWPKNPPEYREVNEEYAVHVKKLSETLLGILS : 178
Q9XHG2.pro : KLQAVGKEFFE-LPQEEKEAYAKPPDSASIEGYGTKLFKEISEGDTTKKGWVDNLFNKIWPPSVVNYQFWPKNPPSYREANEEYAKHLHNVVEKLFRLLS : 180
Q9FFQ5.pro : RLLQVGMEFFE-LPETEKEAVAKPEDSLDIEGYRTKYQKDLEG----RNAWVDHLFHRIWPPSRVNHKFWPKNPPEYIEVNEEYASHIKKLSEKIMEWLS : 149
O04395.pro : RLQKVGREFFE-LPEAEKRSCAREAG--SVEGYGRRIELDIKK----RKGIVDQIYLSTWPPSSVNYRYWPKSPPDYREVNEEYARHVKTLSEKIMEWLS : 135
Q9FFQ4.pro : QLQMVGTQFFE-LPDAEKETVAKEED---FEGYKKNYLGGINN-------WDEHLFHRLSPPSIINYKYWPKNPPQYREVTEEYTKHMKRLTEKILGWLS : 162
TR6321|c0_ : NLQEAGKTFFE-LPAEEKELYARPPGANHLEGYGTKLQKEMEG----KKTWVDYLFHNIWPPARINYQFWPKNPASYREANEEYAKHLRLVADKLFSYLS : 180
 Lq vG FFE 6P EKe a4 2GYgt k k wvd 65h wPps 6Ny 5WPknPp Yre nEEYa 6 6 6 LS

 * 220 * 240 * 260 * 280 * 300
Q9ZWQ9.pro : LGLGVEGGVLKEAAGGDDIEYMLKINYYPPCPRPDLALGVVAHTDLSALTVLVPNEVPGLQVFKDDRWIDAKYIPNALVIHIGDQIEILSNGKYKAVLHR : 278
Q9M547.pro : LGLDLEPNSFKDGAGGDDLVYLMKINYYPPCPRPDLALG-VAHTDMSAITVLVPNEVPGLQVYKDGHWYDCKYIPNALIVHIGDQVEIMSNGKYKSVYHR : 277
Q07512.pro : LGLGLEGHEMIEAAGGDEIVYLLKINYYPPCPRPDLALGVVAHTDMSYITILVPNEVQGLQVFKDGHWYDVKYIPNALIVHIGDQVEILSNGKYKSVYHR : 291
Q7XZQ6.pro : LGIGLEKHELKKASGGDDLIYMLKINYYPPCPRPDLALGVVAHTDMSAITILVPNEVQGLQVHKDDHWYDVKYIPNALIIHIGDQIEIMSNGKYKSVYHR : 280
Q41452.pro : LGLGLEGHEMMEAAGSEDIVYMLKINYYPPCPRPDLALGVVAHTDMSYITLLVPNEVQ---VFKDGHWYDVNYIPNAIIVHIGDQVEILSNGKYKSVYHR : 292
Q96330.pro : DGLGLKRDALKEGLGGEMAEYMMKINYYPPCPRPDLALGVPAHTDLSGITLLVPNEVPGLQVFKDDHWFDAEYIPSAVIVHIGDQILRLSNGRYKNVLHR : 278
Q9XHG2.pro : LGLGLEGQELKKAAGGDNLEYLLKINYYPPCPRPDLALGVVAHTDMSTVTILVPNDVQGLQACKDGRWYDVKYIPNALVIHIGDQMEIMSNGKYTSVLHR : 280
Q9FFQ5.pro : EGLGLRHEALKEGLGGETIEYLMKINYYPPCPDPELVVGAPDHTDVNGITLLVANEALGLQAFKDNQWIDAEYTTSGIIVIIGDQFLRMSNGKYKSVEHR : 249
O04395.pro : EGLGLGREAIKEVNG---CWYVMNINHYPPYPHSDSFNGLEPHTDINGLTLIITNEIPGLQVFKDDHWIEVEYIPSAIIVNIGDQIMMLSNGKYKNVLHK : 232
Q9FFQ4.pro : EGLGLQRETFTQSIGGDTAEYVLRVNFYPPTQDTELVIGAAAHSDMGAIALLIPNEVPGLQAFKDEQWLDLDYIDSAVVVIIGDQLMRMTNGRLKNVLHR : 262
TR6321|c0_ : LGIGLEPNVLKDALGGEEVEYLLKINYYPPCPRPDLTLGVAPHTDMSAITILVPNEVAGLQVFKDDRWFDAKYIPNALIIHIGDQIQILSNGKYKSVFHR : 280
 G6g6 Gg Y66 6N YPPcp pdl G H3D6 6t666pNe glq KD W d Yip a666 IGDQ 63NG4yk V H4

 * 320 * 340 * 360
Q9ZWQ9.pro : TTVNKDKTRMSWPVFLEPPADTVVGPLPQLVD-DENPPKYKAKKFKDYSYCKLN-----KLPQ-- : 335
Q9M547.pro : TTVNKEKTRMSWPVFLEPPPDHEVGPIPKLVN-EENPAKFKTKKYKDYAYCKLN-----KLPQ-- : 334
Q07512.pro : TTVNKDKTRMSWPVFLEPPSEHEVGPIPKLLS-EANPPKFKTKKYKDYVYCKLN-----KLPQ-- : 348
Q7XZQ6.pro : TTVNKDKTRMSWPVFLEPPPELLTGPISKLIT-DENPAKFKTKKYKDYVYCKLN-----KLPQ-- : 337
Q41452.pro : TTVNKYKTRMSWPVFLEPSSEHEVGPIPNLIN-EANPPKFKTKKYKDYVYCKLN-----KLPQ-- : 349
Q96330.pro : TTVDKEKTRMSWPVFLEPPREKIVGPLPELTG-DDNPPKFKPFAFKDYSYRKLN-----KLPLD- : 336
Q9XHG2.pro : TTVNKDKTRISWPVFLEPPADHVVGPHPQLVN-AVNQPKYKTKKYGDYVYCKIN-----KLPQ-- : 337
Q9FFQ5.pro : AKMDKEKTRISWPVFVESSLDQVFGPLPELITGDENVPKFKPYVYKDYKFRKLK-----KLLLD- : 308
O04395.pro : TTVDKEKTRMSWPVLVSPTYDMVVGPLPELTS-EDDPPKFKPIAYKDYVHNKIT-----FLKNKS : 291
Q9FFQ4.pro : AKSDKDKLRISWPVFVAPRADMSVGPLPEFTG-DENPPKFETLIYNDYIDQKIRGWALEDLPVY- : 325
TR6321|c0_ : TTVNKDKTRMSWPVFCSPPPEHVIGPLPQLVD-EENPAKFKTKKYKDYEYCKLN-----KLPQ-- : 337
 tt 1K KtR6SWPVf p GP p l 1p K5k 5kDY K6 kLp


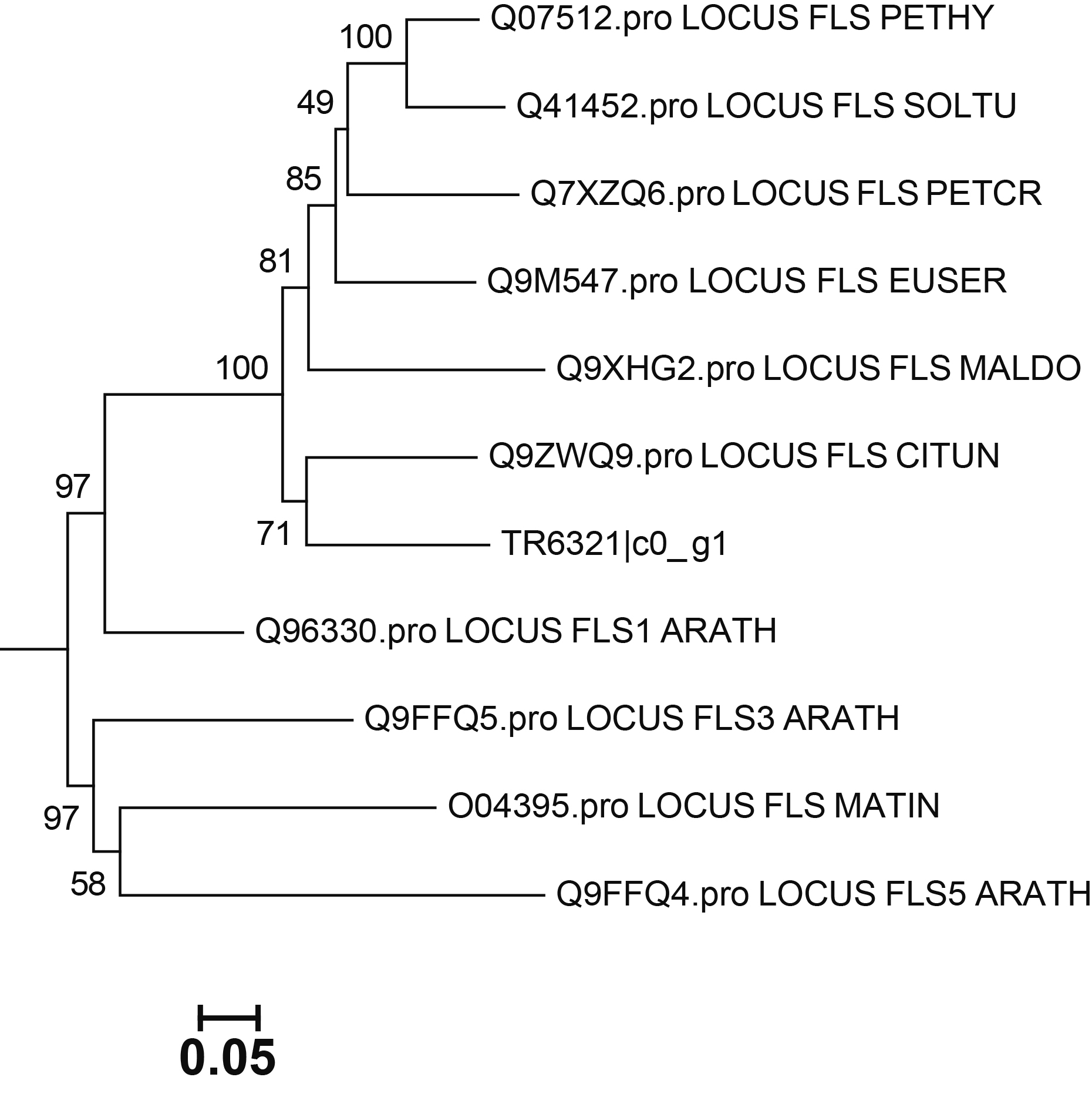

Supplement: S10 Fig — (DOCX) [file pone.0182348.s024.docx]
