# Supplementary material for: Understanding the molecular mechanisms underlying the effects of light intensity on flavonoid production by RNA-seq analysis in Epimedium pseudowushanense B.L.Guo
Source: PLoS One. 2017 Aug 7;12(8):e0182348. doi: 10.1371/journal.pone.0182348 (PMC5546586; doi:10.1371/journal.pone.0182348)

**S11 Fig. Sequence alignment of flavonoid 3'-monooxygenase proteins from *E. pseudowushanense* and various other plants, and phylogenetic relationships of flavonoid 3'-monooxygenase proteins from *E. pseudowushanense* and various other plants.**

* 20 * 40 * 60 * 80 * 100
Q9SD85.pro : ----MATLFLTILLATVLFLILRIFSHRRNRSHNNR----LPPGPNPWPIIGNLPHMGTKPHRTLSAMVTTYGPILHLRLGFVDVVVAASKSVAEQFLKI : 92
Q9SBQ9.pro : ----MEILSLILYTVIFSFLLQFILRSFFRKRYPLP----LPPGPKPWPIIGNLVHLGPKPHQSTAAMAQTYGPLMYLKMGFVDVVVAASASVAAQFLKT : 92
P37120.pro : ----MVILPSELIGATIIYIIVYIIIQKLIATGSWRRR-RLPPGPEGWPVIGALPLLGGMPHVALAKMAKKYGPIMYLKVGTCGMVVASTPNAAKAFLKT : 95
P48418.pro : -----MMLLTELGAATSIFLIAHIIISTLISKTTGRH---LPPGPRGWPVIGALPLLGAMPHVSLAKMAKKYGAIMYLKVGTCGMAVASTPDAAKAFLKT : 92
O04790.pro : -MAVGNGVLLHIAASLMLFFHVQKLVQYLWMNSRRH---RLPPGPIGWPVLGALRLLGTMPHVALANMAKKYGPVMYLKVGSCGLAVASTPEAAKAFLKT : 96
Q96418.pro : -MAVGNGVLLHIARSLMLFFHVQKLVQYLWMNSRRH---RLPPGPIGWPVLGALPLLGTMPHVALANMAKKYGPVMYLKVGSCGLAVASTPEAAKAFLKT : 96
P48419.pro : -----MVLLSELAAATLIFLTTHIFISTLLSITNGRR---LPPGPRGWPVIGALPLLGAMPHVSLAKMAKKYGAIMYLKVGTCGMVVASTPDAAKAFLKT : 92
O04773.pro : MSIDISTLFYELVAAISLYLATYSFIRFLFKPSHHHH---LPPGPTGWPIIGALPLLGTMPHVSLADMAVKYGPIMYLKLGSKGTVVASNPKAARAFLKT : 97
Q96581.pro : MSPIYTTLTLHLATALFLFFHVQKLVHYLHGKATGHRCRRLPPGPTGWPILGALPLLGNMPHVTFANMAKKYGSVMYLKVGSHGLAIASTPDAAKAFLKT : 100
Q42798.pro : ---------MAYQVLLICLVSTIVFAYILWRKQSKKN---LPPSPKALPIIGHLHLVSPIPHQDFYKLSTRHGPIMQLFLGSVPCVVASTAEAAKEFLKT : 88
TR11481|c3 : ----MTPLELVLATLIIATILYTLINSLLNP-APRG----LPPGPKPWPIIGNLLHLGRVPHHSLAALAKKYGPLMHLRLGSVHVIVAASSSVATQIFKT : 91
 l LPPgP wP66G L 6g PH a 6a yG 66 L 6G 6A A flKt

 * 120 * 140 * 160 * 180 * 200
Q9SD85.pro : HDANFASRP-PNSGAKHMAYNYQD--LVFAPYGHRWRLLRKISSVHLFSAKALEDFKHVRQEEVGTLTRELVRVGTK----PVNLGQLVNMCVVNALGRE : 185
Q9SBQ9.pro : HDANFSSRP-PNSGAEHMAYNYQD--LVFAPYGPRWRMLRKICSVHLFSTKALDDFRHVRQDEVKTLTRALASAGQK----PVKLGQLLNVCTTNALARV : 185
P37120.pro : LDINFSNRP-PNAGATHMAYNAQD--MVFAPYGPRWKLLRKLSNLHMLGGKALENWANVRANELGHMLKSMFDASHVGE--RIVVADMLTFAMANMIGQV : 190
P48418.pro : LDINFSNRP-PNAGATHLAYNAQD--MVFAHYGPRWKLLRKLSNLHMLGGKALENWANVRANELGHMLKSMSDMSREGQ--RVVVAEMLTFAMANMIGQV : 187
O04790.pro : LDMNFSNRP-PNAGATHLAYNAQD--MVFADYGPRWKLLRKLSNIHILGGKALQGWEEVRKKELGYMLYAMAESGRHGQ--PVVVSEMLTYAMANMLGQV : 191
Q96418.pro : LDMNFSNRP-PNAGATHLAYNAQD--MVFADYGPRWKLLRKLSNIHILGGKALQGWEEVRKKELGYMLYAMAESGRHGQ--PVVVSEMLTYAMANMLGQV : 191
P48419.pro : LDLNFSNRP-PNAGATHLAYGAQD--MVFAHYGPRWKLLRKLSNLHMLGGKALENWANVRANELGHMLKSMFDMSREGE--RVVVAEMLTFAMANMIGQV : 187
O04773.pro : HDANFSNRP-IDGGPTYLAYNAQD--MVFAEYGPKWKLLRKLCSLHMLGPKALEDWAHVKVSEVGHMLKEMYEQSSKSVPVPVVVPEMLTYAMANMIGRI : 194
Q96581.pro : LDLNFSNRP-PNAGATHLAYNAQD--MVFAHYGPKWKLLRKLSNLHMLGGKALENWADVRKTELGYMLKAMFESSQNNE--PVMISEMLTYAMANMLSQV : 195
Q42798.pro : HEINFSNRPGQNVAVKGLAYDSQDFLFAFAPFGPYWKFMKKLCMSELLSGRMMDQFLPVRQQETKRFISRVFRKGVAG--EAVDFGDELMTLSNNIVSRM : 186
TR11481|c3 : HDVNFSSRP-PNSGAKHIAYNYQD--LVFAPYGPKWRMLRKICSVHLFSAKALDDFRHIRQEEVLVLINALFQAGKA----PADLAKLLNVCTTNALGRV : 184
 d NFs RP p1 ga h6AY QD vFA 5Gp W4 64K6 h6 4a6 5 64 E 6 6 N 6

 * 220 * 240 * 260 * 280 * 300
Q9SD85.pro : MIGRRLFGAD-A-------DHKADEFRSMVTEMMALAGVFNIGDFVPSLDWLDLQGVAGKMKRLHKRFDAFLSSILKEHEMNGQD-------QKHTDMLS : 270
Q9SBQ9.pro : MLGKRVFADGSGD-----VDPQAAEFKSMVVEMMVVAGVFNIGDFIPQLNWLDIQGVAAKMKKLHARFDAFLTDILEEHKGKIFG--------EMKDLLS : 272
P37120.pro : MLSKRVFVEK---------GKEVNEFKNMVVELMTVAGYFNIGDFIPQIAWMDLQGIEKGMKKLHKKFDDLLTKMFEEHEATSNE-R-----KGKPDFLD : 275
P48418.pro : MLSKRVFVDK---------GVEVNEFKDMVVELMTIAGYFNIGDFIPCLAWMDLQGIEKRMKRLHKKFDALLTKMFDEHKATTYE-R-----KGKPDFLD : 272
O04790.pro : MLSKRVFGSQ---------GSESNEFKDMVVELMTVAGYFNIGDFIPSIAWMDLQGIQGGMKRLHKKFDALLTRLLEEHTASAHE-R-----KGSPDFLD : 276
Q96418.pro : MLSKRVFGSQ---------GSESNEFKDMVVELMTVAGYFNIGDFIPSIAWMDLQGIQGGMKRLHKKFDALLTRLLEEHTASAHE-R-----KGSPDFLD : 276
P48419.pro : ILSKRVFVNK---------GVEVNEFKDMVVELMTTAGYFNIGDFIPCLAWMDLQGIEKGMKRLHKKFDALLTKMFDEHKATSYE-R-----KGKPDFLD : 272
O04773.pro : ILSRRPFVITSKLDSSASASASVSEFQYMVMELMRMAGLFNIGDFIPYIAWMDLQGIQRDMKVIQKKFDVLLNKMIKEHTESAHD-R-----KDNPDFLD : 288
Q96581.pro : ILSRRVFNKK---------GAKSNEFKDMVVELMTSAGYFNIGDFIPSIGWMDLQGIEGGMKRLHKKFDVLLTRLLDDHKRTSQE-R-----KQKPDFLD : 280
Q42798.pro : TLSQKTSEND----------NQAEEMKKLVSNIAELMGKFNVSDFIWYLKPFDLQGFNRKIKETRDRFDVVVDGIIKQRQEERRKNKETGTAKQFKDMLD : 276
TR11481|c3 : MLGRRVFGDGSG-------DEKSEEFKQMVVEMMVLAGVFNIGDFVPSLEWLDLQGVASKMKKLHNRFDEFLNKILEEHKESNSEDG-----RAHTDLLS : 272
 6 4 f Ef 6V e6m aG FN6gDF6p 6 w D6QG 6K h 4FD 6 6 h D L

 * 320 * 340 * 360 * 380 * 400
Q9SD85.pro : TLISLKGTDLDGDGGSLTDTEIKALLLNMFTAGTDTSASTVDWAIAELIRHPDIMVKAQEELDIVVGRDRPVNESDIAQLPYLQAVIKENFRLHPPTPLS : 370
Q9SBQ9.pro : TLISLKNDDADNDGGKLTDTEIKALLLNLFVAGTDTSSSTVEWAIAELIRNPKILAQAQQEIDKVVGRDRLVGELDLAQLTYLEAIVKETFRLHPSTPLS : 372
P37120.pro : FIMAN---RDNSEGERLSITNIKALLLNLFTAGTDTSSSVIEWALTEMMKNPTIFKKAQQEMDQIIGKNRRFIESDIPNLPYLRAICKEAFRKHPSTPLN : 372
P48418.pro : VVMEN---GDNSEGERLSTTNIKALLLNLFTAGTDTSSSAIEWALAEMMKNPAILKKAQAEMDQVIGRNRRLLESDIPNLPYLRAICKETFRKHPSTPLN : 369
O04790.pro : FVVAN---GDNSEGERLQTVNIKALLLNMFTAGTDTSSSVIEWALAELLKNPIILRRAQEEMDGVIGRDRRFLEADISKLPYLQAICKEAFRKHPSTPLN : 373
Q96418.pro : FVVAN---RDNSEGERLHTVNIKALLLNMFTAGTDTSSSVIEWALAELLKNPIILKRAQEEMDGVIGRDRRFLEADISKLPYLQAICKEAFRKHPSTPLN : 373
P48419.pro : CVMEN---RDNSEGERLSTTNIKALLLNLFTAGTDTSSSAIEWALAEMMKNPAILKKAQGEMDQVIGNNRRLLESDIPNLPYLRAICKETFRKHPSTPLN : 369
O04773.pro : ILMAAT--QENTEGIQLNLVNVKALLLDLFTAGTDTSSSVIEWALAEMLNHRQILNRAHEEMDQVIGRNRRLEQSDIPNLPYFQAICKETFRKHPSTPLN : 386
Q96581.pro : FVIAN---GDNSDGERLNTDNIKALLLNLFTAGTDTSSSIIEWALAELLKNRTLLTRAQDEMDRVIGRDRRLLESDIPNLPYLQAICKETFRKHPSTPLN : 377
Q42798.pro : VLLDMH--EDENAEIKLDKKNIKAFIMDIFVAGTDTSAVSIEWAMAELINNPDVLEKARQEIDAVVGKSRMVEESDIANLPYLQAIVRETLRLHPGGPLV : 374
TR11481|c3 : VLIGLK-DDADGEGGKLTDTNIKALLLDLFTAGTDTSSSTVEWAIAELIRNPKLLAQAQEELNQVVGRDRLVSESDLGQLTFFQAIIKETFRLHPSTPLS : 371
 66 g L n6KAl6616FtAGTDTSss 6eWA6aE66 np 6 Aq E61 66G R 2 D6 Lp5l A6 4E fR HPstPL

 * 420 * 440 * 460 * 480 * 500
Q9SD85.pro : LPH-IASESCEINGYHIPKGSTLLTNIWAIARDPDQWS-DPLAFKPERFLPGGEKSGVDVKGSDFELIPFGAGRRICAGLSLGLRTIQFLTATLVQGFDW : 468
Q9SBQ9.pro : LPR-IASESCEINGYFIPKGSTLLLNVWAIARDPNAWA-DPLEFRPERFLPGGEKPKVDVRGNDFEVIPFGAGRRICAGMNLGIRMVQLMIATLIHAFNW : 470
P37120.pro : LPR-VSSDACTIDGYYIPKNTRLSVNIWAIGRDPDVWE-NPLEFIPERFLSE-KNAKIEHRGNDFELIPFGAGRRICAGTRMGIVMVEYILGTLIHSFDW : 469
P48418.pro : LPR-ISNEPCIVDGYYIPKNTRLSVNIWAIGRDPQVWE-NPLEFNPERFLSG-RNSKIDPRGNDFELIPFGAGRRICAGTRMGIVMVEYILGTLVHSFDW : 466
O04790.pro : LPR-IASQACEVNGHYIPKGTRLSVNIWAIGRDPSVWE-NPNEFNPDRFLER-KNAKIDPRGNDFELIPFGAGRRICAGTRLGILLVEYILGTLVHSFVW : 470
Q96418.pro : LPR-IASQACEVNGHYIPKGTRLSVNIWAIGRDPSLWE-NPNEFNPDRFLER-KNAKIDPRGNDFELIPFGAGRRICAGTRLGILLVEYILGTLVHSFDW : 470
P48419.pro : LPR-ISNEPCIVDGYYIPKNTRLSVNIWAIGRDPEVWE-NPLEFYPERFLSG-RNSKIDPRGNDFELIPFGAGRRICAGTRMGIVMVEYILGTLVHSFDW : 466
O04773.pro : LPR-ISTEACEVDGFHIPKNTRLIVNIWAIGRDPKVWE-NPLDFTPERFLSE-KHAKIDPRGNHFELIPFGAGRRICAGARMGAASVEYILGTLVHSFDW : 483
Q96581.pro : LPRNCIRGHVDVNGYYIPKGTRLNVNIWAIGRDPSVWGDNPNEFDPERFLYG-RNAKIDPRGNHFELIPFGAGRRICAGTRMGILLVEYILGTLVHSFDW : 476
Q42798.pro : VRE--SSKSAVVCGYDIPAKTRLFVNVWAIGRDPNHWE-KPFEFRPERFIRD-GQNQLDVRGQHYHFIPFGSGRRTCPGASLAWQVVPVNLAIIIQCFQW : 470
TR11481|c3 : LPR-MASESCEIDGYHIPKNSTLLVNVWAIARDPDVWS-EPLEFKPERFLPGGKNAHMDVKGTDFEVIPFGAGRRICAGMSMGMRMVQYVTATLVHGFDW : 469
 6pr c 6 G IPk 3 L N6WAI RDP W P eF PeRF6 6d 4G 5e IPFGaGRRiCaG 6g 6 t66h F W

 * 520 * 540
Q9SD85.pro : ELAGGVTPEKLNMEESYGLTLQRAVPLVVHPKPRLAPNVYGLGSG- : 513
Q9SBQ9.pro : DLVSGQLPEMLNMEEAYGLTLQRADPLVVHPRPRLEAQAYIG---- : 512
P37120.pro : KLPND--VVDINMEETFGLALQKAVPLEAIVTPRLSFDIYQSSEPF : 513
P48418.pro : KLPSE--VIELNMEEAFGLALQKAVPLEAMVTPRLQLDVYVP---- : 506
O04790.pro : ELPSS--VIELNMDESFGLALQKAVPLAAMVTPRLPLHIYSP---- : 510
Q96418.pro : ELPSS--VIELNMDEPFGLALQKAVPLAAMVTPRLPLHIYCP---- : 510
P48419.pro : KLPSE--VIELNMEEAFGLALQKAVPLEAMVTPRLPIDVYAPLA-- : 508
O04773.pro : KLPDG--VVEVNMEESFGIALQKKVPLSAIVTPRLPPSSYTV---- : 523
Q96581.pro : KLGFS--EDELNMDETFGLALQKAVPLAAMVIPRLPLHVYAP---- : 516
Q42798.pro : KLVGG--NGKVDMEEKSGITLPRANPIICVPVPRINPFPTI----- : 509
TR11481|c3 : EMPEGQMVEKLNMEESYGLTLQRAAPLVVHPRPRLAPHVYQTNIV- : 514
 6 61M E G6 Lq4a P6 PR6 y


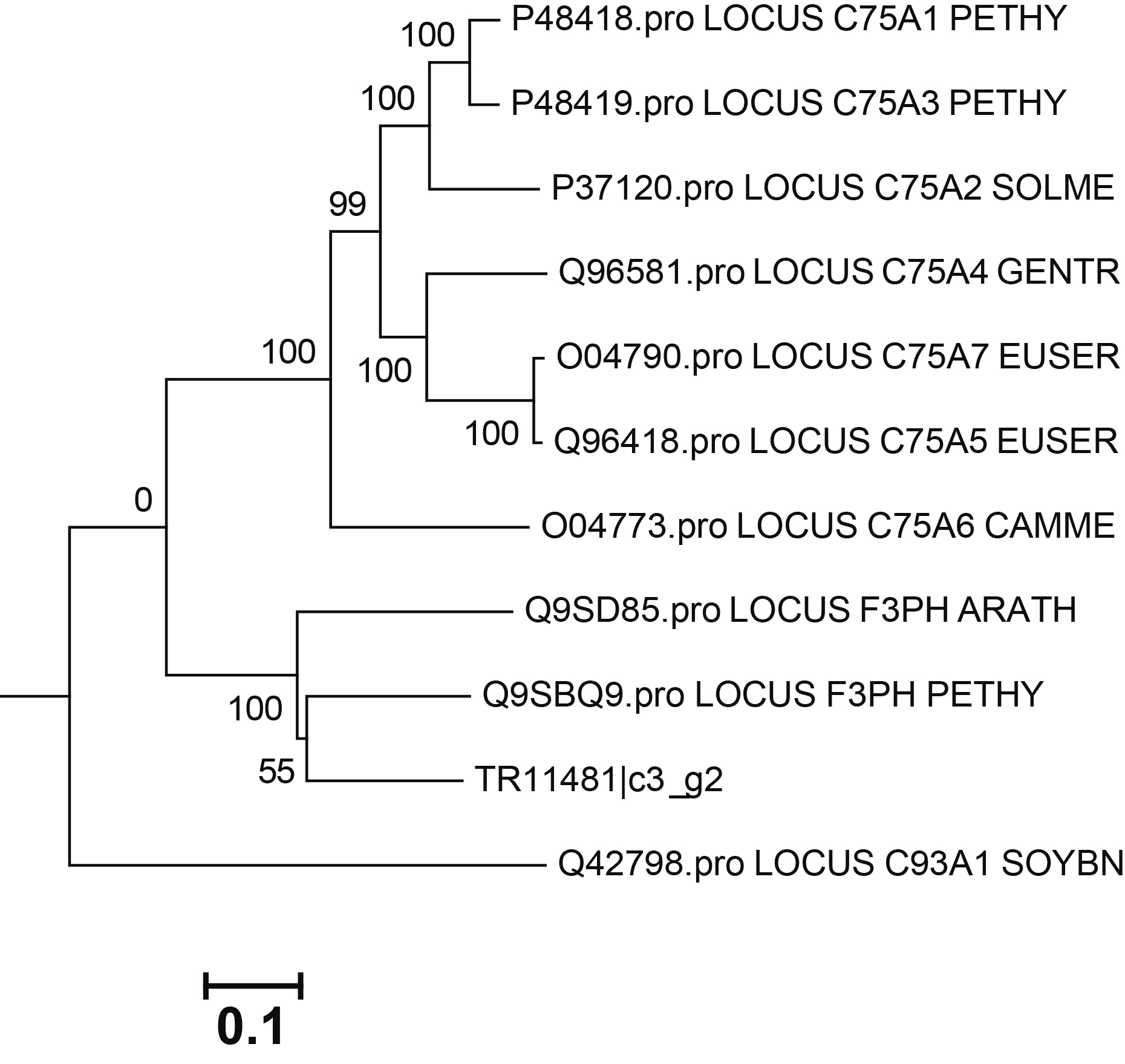

Supplement: S11 Fig — (DOCX) [file pone.0182348.s025.docx]
