# Supplementary material for: Understanding the molecular mechanisms underlying the effects of light intensity on flavonoid production by RNA-seq analysis in Epimedium pseudowushanense B.L.Guo
Source: PLoS One. 2017 Aug 7;12(8):e0182348. doi: 10.1371/journal.pone.0182348 (PMC5546586; doi:10.1371/journal.pone.0182348)

**S12 Fig. Sequence alignment of anthocyanidin reductase proteins (ANR) from *E. pseudowushanense* and various other plants, and phylogenetic relationships of anthocyanidin reductase (ANR) proteins from *E. pseudowushanense* and various other plants.**

* 20 * 40 * 60 * 80 * 100
AFG28175.p : -MATQHPIGKKTACVVGGTGFVASLLVKLLLQKGYAVNTTVRDPDNQKKVSHLLELQKLGDLKIFRADLTDELSFEAPIAGCDFVFHVATPVHFASEDPE : 99
NP_0012678 : -MATQHPIGKKTACVVGGTGFVASLLVKLLLQKGYAVNTTVRDPDNQKKVSHLLELQELGDLKIFRADLTDELSFEAPIAGCDFVFHVATPVHFASEDPE : 99
XP_0102410 : MATQPETVLKRTACVVGGSGFVASLLVKLLLEKGYSVNTTVRDPDNLKKVSHLLELQSLGELKLFRADLTDEGSFDAAVSGCDVVFHVATPVHFASPDPE : 100
AAT68773.p : --MEAQPTAPKAACVVGGTGFVAATLIKLLLEKGYAVNTTVRDPGNQKKTSHLLALKGSGNLKIFRADLTDEQSFDTPVAGCDLVFHVATPVNFASEDPE : 98
TR3386|c0_ : --MDTKPTVVSRTCVTGGTGFMASLLVKHLLEKGYAVNTTARDPENLKKVSHLLELQKLGDLKIFKADLTEEGSFEAAVADCDFVFHVATPVHFESEDPE : 98
 p aCVvGG3GF6AslL6KlLL2KGYaVNTTvRDP N KKvSHLLeLq lG LK6F4ADLTdE SF a 6agCD VFHVATPVhFaSeDPE

 * 120 * 140 * 160 * 180 * 200
AFG28175.p : NDMIKPAIQGVVNVMKACTRAKSVKRVILTSSAAAVTINQLDGTGLVVDEKNWTDIEFLTSAKPPTWGYPASKTLAEKAAWKFAEENNIDLITVIPTLMA : 199
NP_0012678 : NDMIKPAIQGVVNVMKACTRAKSVKRVILTSSAAAVTINQLDGTGLVVDEKNWTDIEFLTSAKPPTWGYPASKTLAEKAAWKFAEENNIDLITVIPTLMA : 199
XP_0102410 : NDMIKPAIQGVLNVLTACKKSKSVKRVILTSSAAAISINKQTGTGLVMDESCWSDTEFLTSEKPPTWGYPVSKTLAEKEAWKFAQENQIDLVTIIPSLMA : 200
AAT68773.p : NDMIKPAIQGVVNVLKACAKAGTVKRVILTSSAAAVSINKLNGTGLVMDESHWTDTEFLNSAKPPTWGYPLSKTLAEKAAWKFAEENNINLITVIPTLMA : 198
TR3386|c0_ : NDMIKPAIQGTVDILRACAKAKTVKRVILTSSAAAVSINKLNGTGLVMNEENWTDVEFLASAKPPTWGYPASKALAEKAAWKYAEENKIDLITVIPSLMA : 198
 NDMIKPAIQGv6166 AC 4ak3VKRVILTSSAAA63IN l GTGLV61E W3D EFL SaKPPTWGYP SKtLAEKaAWK5A2EN I1L6T6IP3LMA

 * 220 * 240 * 260 * 280 * 300
AFG28175.p : GSSLTSDVPSSIGLAMSLITGNEFLINGMKGMQMLSGSVSIAHVEDVCRAHIFVAEKESASGRYICCAANTSVPELAKFLSKRYPQYKVPTDFGDFPSKS : 299
NP_0012678 : GSSLTSDVPSSIGLAMSLITGNEFLINGMKGMQMLSGSVSIAHVEDVCQAHIFVAEKESASGRYICCAANTSVPELAKFLSKRYPQYKVPTDFGDFPPKS : 299
XP_0102410 : GPSLTLDVPSSACLAMSLLTGNEFLINGMKGMQMLSGSISMAHVEDVCRAHIFLAENEMASGRYICCPINTSVTQLAKFLSERYPQYKVPTDFGDFPTKA : 300
AAT68773.p : GPSLTADVPSSIGLAMSLITGNEFLINGLKGMQMLSGSISISHVEDVCRAHVFVAEKESASGRYICCAVSTSVPELAKFLNKRYPEYNVPTDFGDFPSKA : 298
TR3386|c0_ : GPALTPSVPSSICLAMSLLTGNEFLINGMKGMQMLSGSISVTHVEDVVRAHVFLAEKESASGRYICCHINTSIVELAKFLKKRYPQYNVPTDFGDFPEKA : 298
 G sLT dVPSSi LAMSL6TGNEFLING6KGMQMLSGS6S6 HVEDVcrAH6F6AEkEsASGRYICC nTS6 2LAKFL kRYP2Y VPTDFGDFP K

 * 320 * 340
AFG28175.p : KLIISSDKLVKEGFSFKYGIEEIYDESVEYFKAKGLLQN- : 338
NP_0012678 : KLIISSEKLVKEGFSFKYGIEEIYDESVEYFKAKGLLQN- : 338
XP_0102410 : KLILSSEKLIKEGFSFKYGIEEIYDQCVDYFKTKGLLQN- : 339
AAT68773.p : KLILSSEKLTKEGFSFKYGIEEIYDQSVEYFKAKGILKN- : 337
TR3386|c0_ : KLILTSDKLRNEGFSFKYEIEDIYDQSIEYFKTVGLLDK- : 337
 KLI63S KL kEGFSFKYgIEeIYD2s6eYFK kG6L n


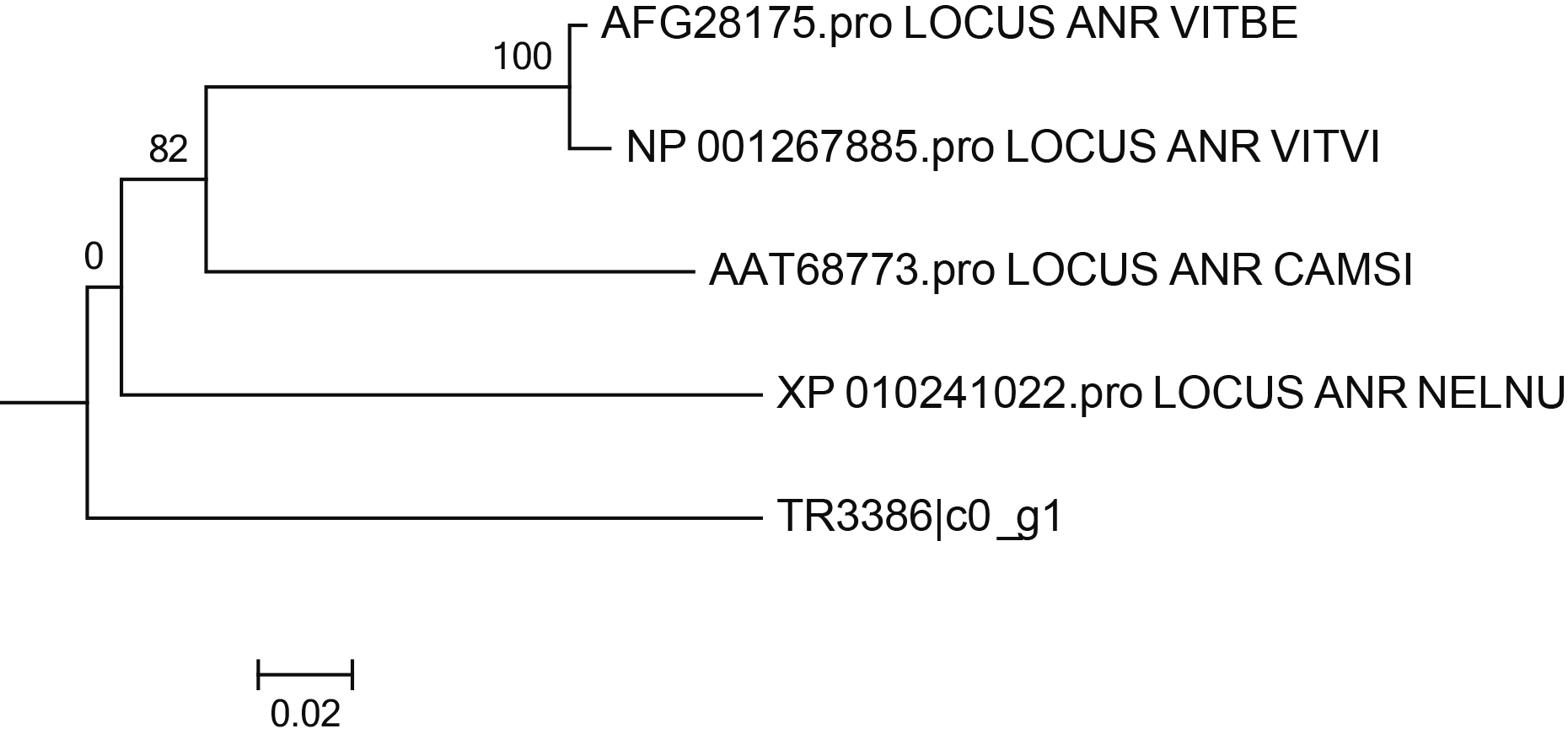

Supplement: S12 Fig — (DOCX) [file pone.0182348.s026.docx]
