# Supplementary material for: Understanding the molecular mechanisms underlying the effects of light intensity on flavonoid production by RNA-seq analysis in Epimedium pseudowushanense B.L.Guo
Source: PLoS One. 2017 Aug 7;12(8):e0182348. doi: 10.1371/journal.pone.0182348 (PMC5546586; doi:10.1371/journal.pone.0182348)

**S13 Fig. Sequence alignment of naringenin 3-dioxygenase proteins from *E. pseudowushanense* and various other plants, and phylogenetic relationships of naringenin 3-dioxygenase proteins from *E. pseudowushanense* and various other plants.**

* 20 * 40 * 60 * 80 * 100
Q06942.pro : ---MAPAT------TLTSIAHEKTLQQ-KFVRDEDERPKVAYNDFSNEIPIISLAGIDEVEG-RRGEICKKIVAACEDWGIFQIVDHGVDAELISEMTGL : 89
P41090.pro : ---MAPTT-------LTALAGEKTLQS-SFVRDEDERPKVAYNDFSNEIPVISLTKESMKLAAVVDEICRKIVEACEDWGIFQVVNHGVDSNLISEMTRL : 89
Q9S818.pro : -----MAPG-----TLTELAGESKLNS-KFVRDEDERPKVAYNVFSDEIPVISLAGIDDVDG-KRGEICRQIVEACENWGIFQVVDHGVDTNLVADMTRL : 88
Q7XZQ7.pro : ---MAPST-------LTALAQEKTLNS-KFVRDEDERPKIAYNKFSDEIPVISLAGIDDDSVDKRSQICRKIVEACEDWGIFQVVDHGIDIDLISEMTRL : 89
Q07353.pro : IPRVTPST-------LTALAEEKTLQT-SFIRDEDERPKVAYNQFSNEIPIISLEGIDDETG-KRAEICDKIVKACEDWGVFQVVDHGVDAEVISQMTTF : 91
Q05965.pro : ------APG-----TLTELAGESKLNS-KFVRDEDERPKVAYNEFSDEIPVISLAGIDDVDG-KRGEICREIVEACENWGIFQVVDHGVDTSLVADMTRL : 87
Q05963.pro : ---MAAP--------ISLKWEEHSLHENKFVRDEDERPKVPYNTFSNEIPVISLAGID--GC-RRAEICDEIVKACEDWGIFQVVDHGVDTKLLSDMTGL : 86
Q05964.pro : --MVAEKPK-----TLTSLEGDDKLNS-NFVRDEDERPKVAYNEFSNDIPVISLAGIDGE---KRGEICRKIVEACEDWGIFQVVDHGVGDDLIADMTRL : 89
Q7XZQ8.pro : ---MAPTT-------ITALAKEKTLNL-DFVRDEDERPKVAYNQFSNEIPIISLAGLDDDSDGRRPEICRKIVKACEDWGIFQVVDHGIDSGLISEMTRL : 89
P28038.pro : MAPVSNETF-----LPTEAWGEATLRP-SFVRDEDERPKVAHDRFSDAVPLISLHGIDGA---RRAQIRDRVAAACEDWGIFQVIDHGVDADLIADMTRL : 91
TR4169|c0_ : ---MAPAAAPSKPSTLTALVDEKKLQQ-SFVRDEDERPKVAYNVFSNEIPVISLAGIDEVDG-RRSEICKKIVDACEDWGIFQVVDHGVDTDLITEMTKL : 95
 3 e L F6RDEDERPK6ay1 FS1e6P6ISL g d r 2Ic 6v ACE1WG6FQ661HG6d 66 MT l

 * 120 * 140 * 160 * 180 * 200
Q06942.pro : AREFFALPSEEKLRFDMSGGKKGGFIVSSHLQGEAVQDWREIVTYFSYPIRHRDYSRWPDKPEAWREVTKKYSDELMGLACKLLGVLSEAMGLDTEALTK : 189
P41090.pro : AREFFALPPEENVRFDMSGGKKGGFIVSSHLQGEAVQDWREIVTYFSYPLRTRDYSRWPDKPEGWRSVTQEYSEKLMGLACKLLEVLSEAMDLDKDALTN : 189
Q9S818.pro : ARDFFALPPEDKLRFDMSGGKKGGFIVSSHLQGEAVQDWREIVTYFSYPVRNRDYSRWPDKPEGWVKVTEEYSERLMSLACKLLEVLSEAMGLEKESLTN : 188
Q7XZQ7.pro : ARQFFALPAEEKLRFDMTGGKKGGFIVSSHLQGEAVQDWREIVTYFSYPIQARDYSRWPDKPEGWRSITEMYSDELMALACKLLEVLSEAMGLEKEGLTK : 189
Q07353.pro : AKEFFALPPEEKLRFDMSGGKKGGFIVSSHLQGEVVQDWREIVTYFSYPTRARDYSRWPDKPEGWIAVTQKYSEKLMELACKLLDVLSEAMGLEKEALTK : 191
Q05965.pro : ARDFFALPPEEKLRFDMSGGKKGGFIVSSHLQGEAVQDWREIVTYFSYPVRNRDYSRWPDKPQGWAKVTEEYSEKLMGLACKLLEVLSEAMGLEKESLTN : 187
Q05963.pro : ARDFFHLPTQEKLRFDMTGGKKGGFIVSSHLQGEAVQDWREIVTYFSYPIKARDYSRWPDKPNEWRAVTEEYSKVLMGLACKLLEVLSEAMGLEKEALTK : 186
Q05964.pro : AREFFALPAEEKLRFDMSGGKKGGFIVSSHLQGEVVQDWREIVTYFSYPTNSRDYTRWPDKPEGWIKVTEEYSNKLMTLACTLLGVLSEAMGLELEALTK : 189
Q7XZQ8.pro : SREFFALPAEEKLEYDTTGGKRGGFTISTVLQGDDAMDWREFVTYFSYPINARDYSRWPKKPEGWRSTTEVYSEKLMVLGAKLLEVLSEAMGLEKGDLTK : 189
P28038.pro : AREFFALPAEDKLRYDMSGGKKGGFIVSSHLQGEAVQDWREIVTYFSYPVKARDYGRWPEKPAGWCAVVERYSERLMGLSCNLMGVLSEAMGLETEALAK : 191
TR4169|c0_ : AREFFALPPEDKLRFDMTGGKKGGFIVSSHLQGEAVSDWREIVTYFSYPIRARDYSRWPDTPAAWRAVTQEYSDKLMGLACKLLEVLSEAMDLEKEALTK : 195
 a4 FFaLP 2 k6r5Dm3GGK4GGFi6S3hLQGe vqDWREiVTYFSYP RDY RWPdkP W t YS LM LackL6 VLSEAMgLe e Lt

 * 220 * 240 * 260 * 280 * 300
Q06942.pro : ACVDMDQKVVVNFYPKCPQPDLTLGLKRHTDPGTITLLLQDQVGGLQATRDDGKTWITVQPVEGAFVVNLGDHGHLLSNGRFKNADHQAVVNSNSSRLSI : 289
P41090.pro : ACVDMDQKVVVNFYPQCPQPDLTLGLKRHTDPGTITLLLQDQVGGLQATRDGGKTWITVQPVEGAFVVNLGDHGHYLSNGRFKNADHQAVVNSNHSRLSI : 289
Q9S818.pro : ACVDMDQKIVVNYYPKCPQPDLTLGLKRHTDPGTITLLLQDQVGGLQATRDNGKTWITVQPVEGAFVVNLGDHGHFLSNGRFKNADHQAVVNSNSSRLSI : 288
Q7XZQ7.pro : ACVDMDQKVIVNYYPKCPQPNLTLGLKRHTDPGTITLLLQDQVGGLQATRDGGKTWITVQPVEGAFVVNLGDHGHYLSNGRFKNADHQAVVNSNSSRMSI : 289
Q07353.pro : ACVDMDQKVVVNFYPKCPEPDLTLGLKRHTDPGTITLLLQDQVGGLQATKDNGKTWITVQPVEGAFVVNLGDHGHFLSNGRFKNADHQAVVNSNSSRLSI : 291
Q05965.pro : ACVDMDQKIVVNYYPKCPQPDLTLGLKRHTDPGTITLLLQDQVGGLQATRDDGNTWITVQPVEGAFVVNLGDHGHFLSNGRFKNADHQAVVNSNSSRLSI : 287
Q05963.pro : ACVDMDQKVVVNYYPKCPQPDLTLGLKRHTDPGTITLLLQDQVGGLQATRDGGESWITVKPVEGAFVVNLGDHGHYLSNGRFKNADHQAVVNSSTSRLSI : 286
Q05964.pro : ACVDMDQKIVVNYYPKCPQPDLTLGLKRHTDPGTITLLLQDQVGGLQATRDGGKTWITVQPVPGAFVVNLGDHGHFLSNGRFKNADHQAVVNSECSRLSI : 289
Q7XZQ8.pro : ACVDMEQKVLINYYPTCPQPDLTLGVRRHTDPGTITILLQDMVGGLQATRDGGKTWITVQPVEGAFVVNLGDHGHYLSNGRFRNADHQAVVNSTSSRLSI : 289
P28038.pro : ACVDMDQKVVVNFYPRCPQPDLTLGLKRHTDPGTITLLLQDLVGGLQATRDGGKNWITVQPISGAFVVNLGDHGHFMSNGRFKNADHQAVVNGESSRLSI : 291
TR4169|c0_ : ACVDMDQKVVVNFYPKCPQPDLTLGLKRHTDPGTITLLLQDQVGGLQATRDDGKTWITVQPVEGAFVVNLGDHGHYLSNGRFKNADHQAVVNSNYSRLSI : 295
 ACVDMdQK666N5YP CP2P1LTLG64RHTDPGTIT6LLQDqVGGLQAT4D Gk WITVqP6eGAFVVNLGDHGH 6SNGRF4NADHQAVVNs SR6SI

 * 320 * 340 * 360 * 380 *
Q06942.pro : ATFQNPAQEAIVYPLSVREGEKPILEAPITYTEMYKKKMSKDLELARLKKLAKEQQSQ----DLEKAKVD----------TKPVDDIFA- : 364
P41090.pro : ATFQNPAPEATVYPLKIREGEKAVLEGPITFAEMYRRKMSKDLELARLKKLAKEQQLQ----DVEKAKLE----------SKPIDQILA- : 364
Q9S818.pro : ATFQNPAPDATVYPLKVREGEKAILEEPITFAEMYKRKMGRDLELARLKKLAKEERDHK---EVDKP----------------VDQIFA- : 358
Q7XZQ7.pro : ATFQNPAPNATVYPLKIREGEKAVMEEPITFAEMYKRKMSRDIEMATLKKLAKEKVLQDQEVEKAKLQMT----------PKSADEIFA- : 368
Q07353.pro : ATFQNPAPEAIVYPLKIREGEKSIMDEPITFAEMYRRKMSKDLELARLKKQAKEQQLQA-EVAAEKAKLE----------SKPIEEILA- : 369
Q05965.pro : ATFQNPAPEATVYPLKVREGEKAIMEEPITFAEMYKRKMGRDLELARLKKLAKEEHNHK---EAAKP----------------LDQILA- : 357
Q05963.pro : ATFQNPAPEAIVYPLKINEGEKSIMEEPMTFMEMYKKKMSTDLELARLKKLAKDKQQ-----DLEVVKPI----------QN----IFA- : 356
Q05964.pro : ATFQNPSPDATVYPLAIREGENSIMEEPITFADLYRRKMAKDLEIARHKRLAKEEMPFK---ELDEAK----------FESKSIDQILA- : 365
Q7XZQ8.pro : ATFQNPAQNAIVYPLKIREGEKAILDEAITYAEMYKKCMTKHIEVATRKKLAKEKRLQD---EKAKLEMK----------SKSADENLA- : 365
P28038.pro : ATFQNPAPDARVWPLAVREGEEPILEEPITFTEMYRRKMERDLDLAKRKKQAKDQLMQQ---QLQLQQQQAVAAAPMPTATKPLNEILA- : 377
TR4169|c0_ : ATFQNPAPDATVYPLKIREGEKPILDAPITFAEMYKKKMSRDLELAALKKLAKEQKLE----DLEKVDLK----------PK--E-IFA- : 367
 ATFQNPap A V5PL 6rEGEk 66 p6T5 e6Y44kM d6e6A K4lAKe i A


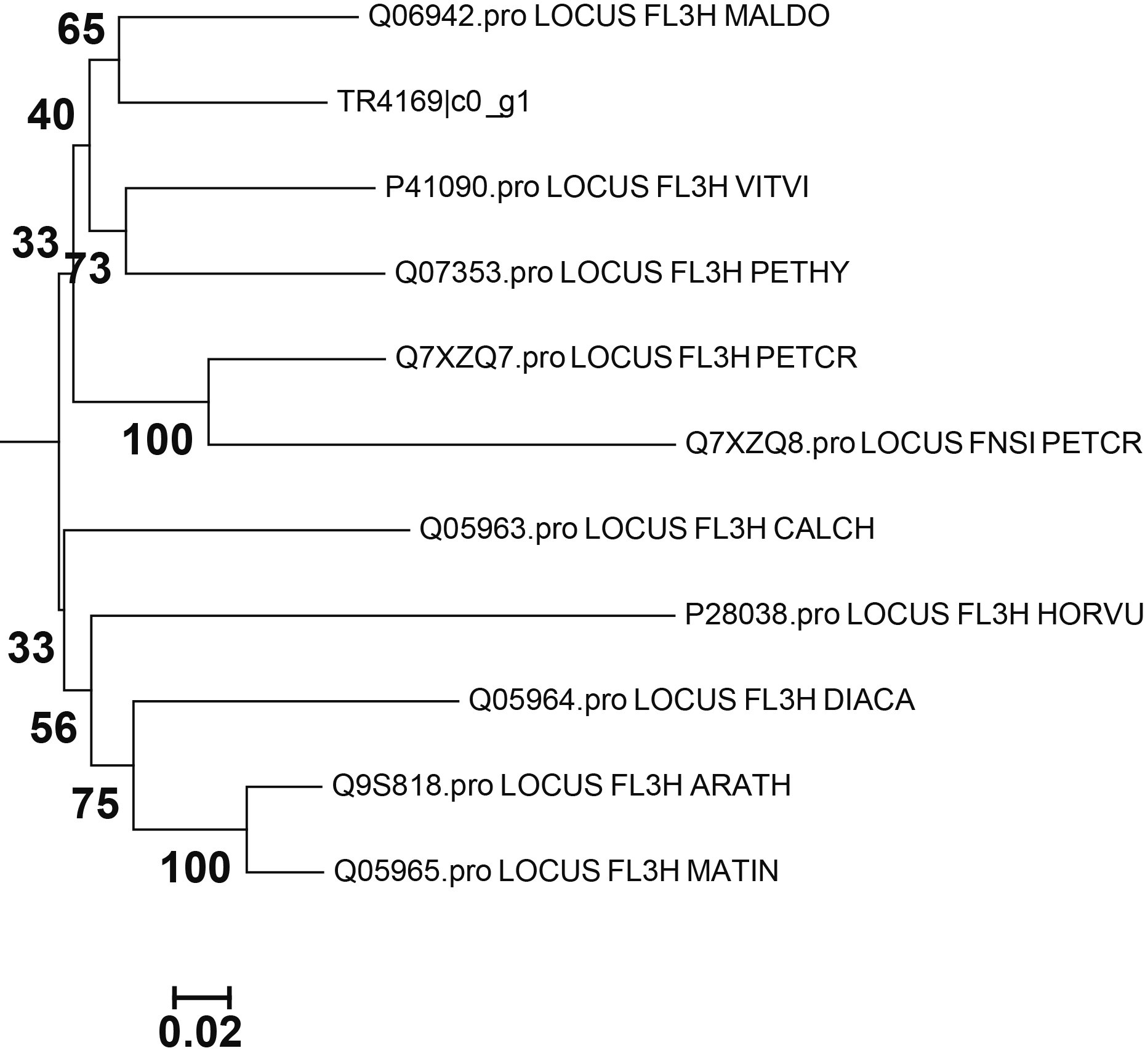

Supplement: S13 Fig — (DOCX) [file pone.0182348.s027.docx]
