# Supplementary material for: Understanding the molecular mechanisms underlying the effects of light intensity on flavonoid production by RNA-seq analysis in Epimedium pseudowushanense B.L.Guo
Source: PLoS One. 2017 Aug 7;12(8):e0182348. doi: 10.1371/journal.pone.0182348 (PMC5546586; doi:10.1371/journal.pone.0182348)

**S14 Fig. Sequence alignment of bifunctional dihydroflavonol 4-reductase/flavanone 4-reductase proteins from *E. pseudowushanense* and various other plants, and phylogenetic relationships of bifunctional dihydroflavonol 4-reductase/flavanone 4-reductase proteins from *E. pseudowushanense* and various other plants.**

* 20 * 40 * 60 * 80 * 100
Q9XES5.pro : --------MGS-----------------ESESVCVTGASGFIGSWLVMRLLEHGYTVRATVRDPTNQKKVKHLLDLPKAETHLTLWKADLADEGSFDEAI : 75
P51110.pro : --------MGS-----------------QSETVCVTGASGFIGSWLVMRLLERRLTVRATVRDPTNVKKVKHLLDLPKAETHLTLWKADLADEGSFDEAI : 75
Q84KP0.pro : --------MGS-----------------ESESVCVTGASGFIGSWLVMRLLEHGYTVRATVRDPTNQKKVKHLLDLPKAETHLTLWKADLADEGSFDEAI : 75
P51102.pro : --------MVS-----------------QKETVCVTGASGFIGSWLVMRLLERGYFVRATVRDPGNLKKVQHLLDLPNAKTLLTLWKADLSEEGSYDDAI : 75
P51104.pro : --------MVSSTINETLDGKHDINKVGQGETVCVTGASGFIGSWLIMRLLERGYTVRATVRDPDNTKKVQHLLDLPNAKTNLTLWKADLHEEGSFDAAV : 92
P51105.pro : --------MEEDSPA----------------TVCVTGAAGFIGSWLVMRLLERGYVVHATVRDPGDLKKVKHLLELPKAQTNLKLWKADLTQEGSFDEAI : 76
P14720.pro : ---MASEAVHAPSPPV------------AVPTVCVTGAAGFIGSWLVMRLLERGYNVHATVRDPENKKKVKHLLELPKADTNLTLWKADLTVEGSFDEAI : 85
P14721.pro : MSPTSLNTSSETAPPS------------ST-TVCVTGAAGFIGSWLVMRLLERGYTVRATVRDPGNMKKVKHLIELPKADTNLTLWKADMTVEGSFDEAI : 87
P51106.pro : --------MDG-----------------NKGPVVVTGASGFVGSWLVMKLLQAGYTVRATVRDPANVEKTKPLLELPGAKERLSIWKADLSEDGSFNEAI : 75
P51103.pro : --------MKEDSPP----------------TVCVTGAAGFIGSWLVMRLLERGYIVRATVRNPGDMKKVKHLLELPKAETNLTLWKADLTQEGSFDEAI : 76
TR19880|c0 : --------MVA-----------------VDETVCVTGAAGFVGSWLVMRLLECGYKVKATVRDPANMKKVKHLLDLPNAKTQLSLFKADLVDEGSFDEAI : 75
 VcVTGA GF6GSWL6M4LL2 gy V ATVR1P 1 kKvkhL6 LP A t L 65KAD6 eGS51eA6

 * 120 * 140 * 160 * 180 * 200
Q9XES5.pro : QGCSGVFHVATPMDFESKDPENEVIKPTINGLLDILKACQKAKT-VRKLVFTSSAGTVNVEEHQKPVYDESNWSDVEFCRSVKMTGWMYFVSKTLAEQAA : 174
P51110.pro : KGCTGVFHVATPMDFESKDPENEVIKPTIEGMLGIMKSCAAAKT-VRRLVFTSSAGTVNIQEHQLPVYDESCWSDMEFCRAKKMTAWMYFVSKTLAEQAA : 174
Q84KP0.pro : QGCSGVFHVATPMDFESRDPENEVIKPTINGLLDILKACQKAKT-VRKLVFTSSAGTVNVEEHQKPVYDESNWSDVEFCRSVKMTGWMYFVSKTLAEQAA : 174
P51102.pro : NGCDGVFHVATPMDFESKDPENEVIKPTVNGMLGIMKACVKAKT-VRRFVFTSSAGTVNVEEHQKNVYDENDWSDLEFIMSKKMTGWMYFVSKTLAEKAA : 174
P51104.pro : DGCTGVFHIATPMDFESKDPENEMIKPTINGMLDILKSCVKAK--LRRVVFTSSGGTVNVEATQKPVYDETCWSALDFIRSVKMTGWMYFVSKILAEQAA : 190
P51105.pro : QGCHGVFHLATPMDFESKDPENEIIKPTIEGVLSIIRSCVKAKT-VKKLVFTSSAGTVNGQEKQLHVYDESHWSDLDFIYSKKMTAWMYFVSKTLAEKAA : 175
P14720.pro : QGCQGVFHVATPMDFESKDPENEVIKPTVRGMLSIIESCAKANT-VKRLVFTSSAGTLDVQEQQKLFYDQTSWSDLDFIYAKKMTGWMYFASKILAEKAA : 184
P14721.pro : QGCEGVFHLATSMEFDSVDPENEVIKPTIDGMLNIIKSCVQAKT-VKKFIFTTSGGTVNVEEHQKPVYDETDSSDMDFINSKKMTGWMYFVSKILAEKAG : 186
P51106.pro : AGCTGVFHVATPMDFDSQDPENEVIKPTVEGMLSIMRACKEAGT-VKRIVFTSSAGSVNIEERPRPAYDQDNWSDIDYCRRVKMTGWMYFVSKALAEKAA : 174
P51103.pro : EGCHGVFHVATPMDFESKDPENEIIKPTIEGILSIIRSCAKAKT-VKKLVYTSSAGTVNVQETQLPVYDESHWSDLDFIYSKKMTAWMYFVSKTLAEKAA : 175
TR19880|c0 : AGCTGVFHVATPMDFESQDPENEVIKPTIEGMLNIMRSCAKAKNTIRRIVFTSSAGTVNVEEHQKPEYNEECWSDMEFVRSKKMTAWMYFVSKTLAEKAA : 175
 GC GVFH6ATpMdFeS DPENE6IKPT6 G6L I6 C Akt 644 65T3SaG361 2e q Y12 wSd6 5 KMT WMYFvSK LAE Aa

 * 220 * 240 * 260 * 280 * 300
Q9XES5.pro : WKYAKENNIDFITIIPTLVIGPFLMPSMPPSLITGLSPILRNESHYGIIKQGQYVHLDDLCLSHIYLYEHPKAEGRYICSSHDATIHELVKMLREKYPEY : 274
P51110.pro : WKYAKENNIDFITIIPTLVVGPFIMSSMPPSLITALSPITGNEAHYSIIRQGQFVHLDDLCNAHIYLFENPKAEGRYICSSHDCIILDLAKMLREKYPEY : 274
Q84KP0.pro : WKYAKENNIDFITIIPTLVIGPFLMPSMPPSLITGLSPILRNESHYGIIKQGQYVHLDDLCLSHIYLYKHPKAEGRYICSSHDATIHELVKMLREKYPEY : 274
P51102.pro : WDFAEEKGLDFISIIPTLVVGPFITTSMPPSLITALSPITRNEAHYSIIRQGQYVHLDDLCNAHIFLYEQAAAKGRYICSSHDATILTISKFLRPKYPEY : 274
P51104.pro : WKYAAENNLEFISIIPPLVVGPFIMPSMPPSLITALSPITRTESHYTIIKQGQFVHLDDLCMSHIFLYENPKANGRYIASACAATIYDIAKMLREEYPEY : 290
P51105.pro : WDATKGNNISFISIIPTLVVGPFITSTFPPSLVTALSLITGNEAHYSIIKQGQYVHLDDLCECHIYLYENPKAKGRYICSSHDATIHQLAKIIKDKWPEY : 275
P14720.pro : MEEAKKKNIDFISIIPPLVVGPFITPTFPPSLITALSLITGNEAHYCIIKQGQYVHLDDLCEAHIFLYEHPKADGRFICSSHHAIIYDVAKMVREKWPEY : 284
P14721.pro : MEAAKENNIDFISIIPPLVVGPFIMPTFPPSLITALSPITGNEAHYSIIKQCQYVHLDDLCEGHIFLFEYPKAEGRYICSSHDATIYDIAKLITENWPEY : 286
P51106.pro : MEYASENGLDFISIIPTLVVGPFLSAGMPPSLVTALALITGNEAHYSILKQVQLVHLDDLCDAMTFLFEHPEANGRYICSSHDATIHGLARMLQDRFPEY : 274
P51103.pro : MEAAKENNIDFVSIIPPLVVGPFINPTFPPSLITALSLINGAESHYSIIKQGQYVHLDDLCECHIFLYENPEAKGRYICSKQDATIHQLARMIKQKWPEY : 275
TR19880|c0 : WDFAKEHNLDFISIIPTLVIGSFLMPSMPPSLITGLSPITRNESHYSIIKQGQFVHLDDLCIAHIFLFEHPEAKGRYICSSHDDTIFNLAKMLRERFPEY : 275
 a e n6dF63IIP LV6GpF6 PPSL6T Ls I nE HY I64QgQ VHLDDLC hi5L5e p A GR5IcSshdatI 6 4 6 5PEY

 * 320 * 340 * 360 * 380 * 400
Q9XES5.pro : NIPTKFKGIDDNLEPVHFSSKKLREIGFEFKYSLEDMFVGAVDACRAKGLIPIPIP----------AE-K---------------TEAAEESNLVDVKVG : 348
P51110.pro : NIPTEFKGVDENLKSVCFSSKKLTDLGFEFKYSLEDMFTGAVDTCRAKGLLRPS------------------------------------HEKPVDGKT- : 337
Q84KP0.pro : NIPTKFKGIDDNLEPVHFSSKKLREIGFEFKYSLEDMFVGAVDACRAKGLIPIP------------AE-K---------------TEAAEESNLVDVKVG : 346
P51102.pro : NVPSTFEGVDENLKSIEFSSKKLTDMGFNFKYSLEEMFIESIETCRQKGFLPVSLS----------YQSI---------------SEIKTKNENIDVKTG : 349
P51104.pro : NVPTKFKDYKEDMGQVQFSSKKLTDLGFEFKYGLKDMYTAAVESCRAKGLLPLSLE----------HH-L---------------CVFRVT--LIFFK-- : 360
P51105.pro : YIPTKFPGIDEELPIVSFSSKKLIDTGFEFKYNLEDMFKGAIDTCREKGLLPYST----------IKNHIN---------------GNHVNGVHHYIKNN : 350
P14720.pro : YVPTEFKGIDKDLPVVSFSSKKLTDMGFQFKYTLEDMYKGAIDTCRQKQLLPFST------------RSAE---------------DNGHNREAIAISAQ : 357
P14721.pro : HIPDEFEGIDKDIPVVSFSSKKMIGMGFIFKYTLEDMVRGAIDTCREKGMLPYSTKNNKGDEKEPILNSLENNYNIQDKELFPISEEKHINGQENALLSN : 386
P51106.pro : DIPQKFAGVDDNLQPIHFSSKKLLDHGFSFRYTTEDMFDAAIHTCRDKGLIPLG-------------DVP----------------APAAGGKLGALAAG : 345
P51103.pro : HVPTQFAGIDEELPTVSFSSKKLIDMGFKFKYDLEDMFKGAIDSCKEKGFLPYSTNE--------VKKGLF---------------ESSINGNVHGQKGN : 352
TR19880|c0 : NVPTKLGNIDENVSVVKFSSKKLLDLGFQYKYSFEDMFVDAVETCRKKGLLPLAFE----------AEANG--------------QCLESKKCEPAIITA : 351
 6P f g d 6 6 FSSKK6 GF 54Y ledM a6 C4 Kg 6p

 * 420 * 440 * 460
Q9XES5.pro : --------------------------------------------------------------- : -
P51110.pro : --------------------------------------------------------------- : -
Q84KP0.pro : S-------------------------------------------------------------- : 347
P51102.pro : D-----------------------GLTDG-------MKPCNKTETGITGERTDAPMLAQQMCA : 382
P51104.pro : --------------------------------------------------------------- : -
P51105.pro : D-----------------------DDHEK------GLLCCSKEGQ------------------ : 366
P14720.pro : N----------------------YASGKE------NAPVANHTEMLSNVEV------------ : 380
P14721.pro : TQDKELLPTSEEKRVNGLESALLSKIQDKEVLPTSGVKHAKGQENALLPDIANDHTDGRI--- : 446
P51106.pro : E----------------------------------GQAIGAET-------------------- : 354
P51103.pro : Q-----------------------KIGDE------GVKLVN---------------------- : 364
TR19880|c0 : P-----------------------AIPNV-------VSP------------------------ : 360


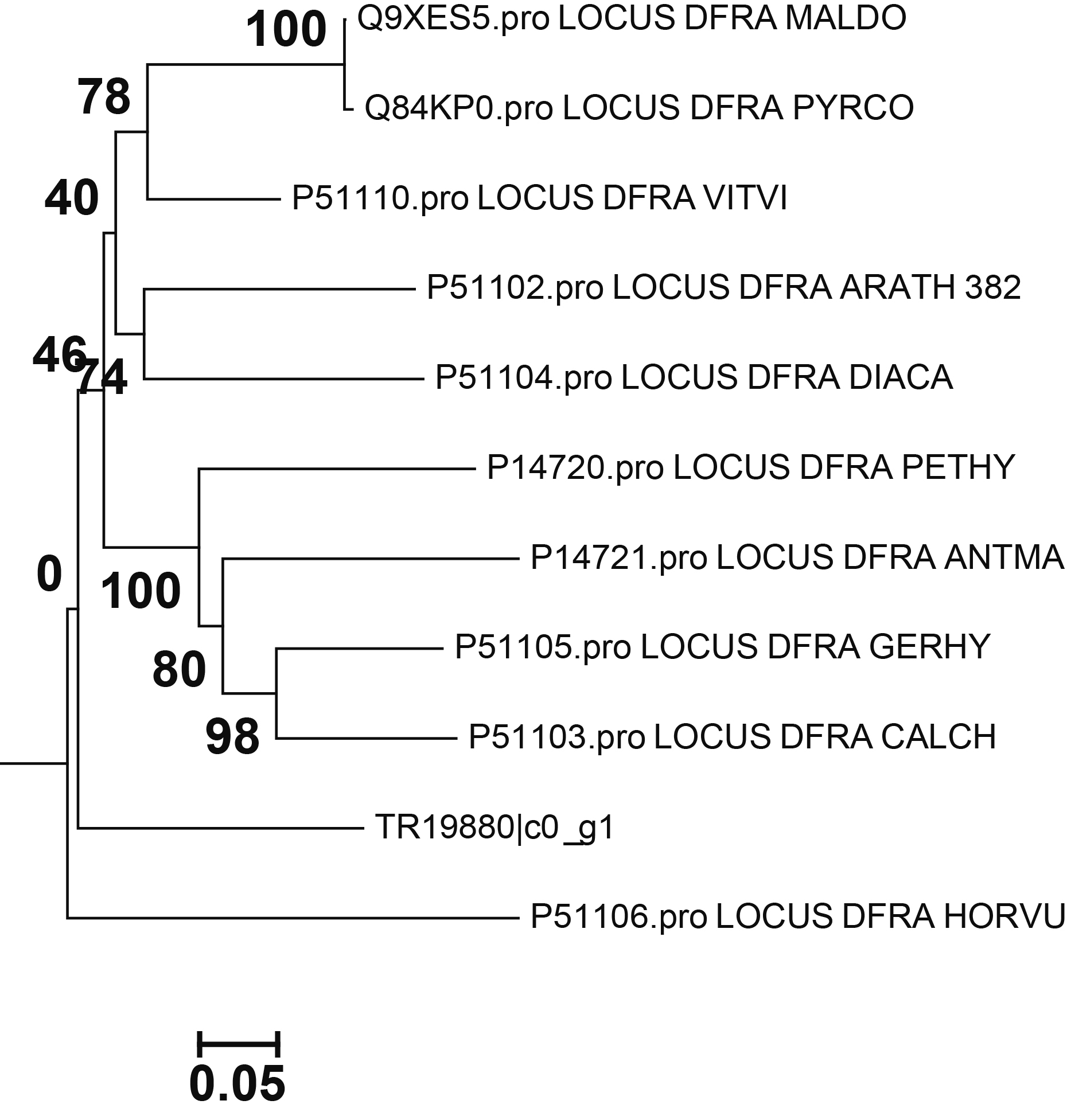

Supplement: S14 Fig — (DOCX) [file pone.0182348.s028.docx]
