# Supplementary material for: Understanding the molecular mechanisms underlying the effects of light intensity on flavonoid production by RNA-seq analysis in Epimedium pseudowushanense B.L.Guo
Source: PLoS One. 2017 Aug 7;12(8):e0182348. doi: 10.1371/journal.pone.0182348 (PMC5546586; doi:10.1371/journal.pone.0182348)

**S15 Fig. Sequence alignment of trans-cinnamate 4-monooxygenase proteins from *E. pseudowushanense* and various other plants, and phylogenetic relationships of trans-cinnamate 4-monooxygenase proteins from *E. pseudowushanense* and various other plants.**

* 20 * 40 * 60 * 80 * 100
Q43240.pro : -MDLLLVEKTLLALFAAIIASIFISKLRGKRFKLPPGPVPVPIFGNWLQVGDDLNHRNLTDLAKKFGEIFLLRMGQRNLVVVSSPNLAKEVLHTQGVEFG : 99
P48522.pro : -MDLLLLEKTLLGLFAAIIVASIVSKLRGKKFKLPPGPIPVPVFGNWLQVGDDLNHRNLSDYAKKFGEIFLLRMGQRNLVVVSSPELAKEVLHTQGVEFG : 99
Q04468.pro : -MDLLLIEKTLVALFAAIIGAILISKLRGKKFKLPPGPIPVPIFGNWLQVGDDLNHRNLTDLAKRFGEILLLRMGQRNLVVVSSPELAKEVLHTQGVEFG : 99
Q42797.pro : -MDLLLLEKTLIGLFLAAVVAIAVSTLRGRKFKLPPGPLPVPIFGNWLQVGDDLNHRNLTDLAKKFGDIFLLRMGQRNLVVVSSPELAKEVLHTQGVEFG : 99
Q43054.pro : -MDLLLLEKTLLGSFVAVLVAILVSKLRGKRFKLPPGPLPVPVFGNWLQVGDDLNHRNLTDLAKKFGDIFLLRMGQRNLVVVSSPDLSKEVLHTQGVEFG : 99
O24312.pro : -MDLLLLEKTLLGSFVAILVAILVSKLRGKRFKLPPGPLPVPVFGNWLQVGDDLNHRNLTDLAKKFGDILLLRMGQRNLVVVSSPELSKEVLHTQGVEFG : 99
Q96423.pro : -MDLLLLEKTLLGLFIAAITAIAISKLRGRRFKLPPGPIPVPIFGNWLQVGDDLNHRNLTDLAKRFGDIFLLRMGQRNLVVVSSPELAKEVLHTQGVEFG : 99
P37115.pro : -MDLLLLEKTLLGLFLAAVVAIVVSKLRGKRFKLPPGPLPVPIFGNWLQVGDDLNHRNLTQLAKRFGDIFLLRMGQRNLVVVSSPDLAKEVLHTQGVEFG : 99
Q43033.pro : MMDFVLLEKALLGLFIATIVAITISKLRGKKLKLPPGPIPVPVFGNWLQVGDDLNQRNLVDYAKKFGDLFMLRMGQRNLVVVSSPELAKDVLHTQGVEFG : 100
P37114.pro : -MDLLLLEKTLLALFIAATIAVTISKLRGKRFKLPPGPIPVPIFGYWLQVGDDLNHRNLTDYAKRFGEIFLLRMGQRNLVVVSSPELAKEVLHTQCVEFG : 99
TR8942|c0_ : -MDLLLLEKSLIASFIAIIVAIAISKLRGKRFKLPPGPFPIPIFGNWLQVGDDLNHRNLTDLARKFGEIFLLRMGQRNLVVVSSPDLAKEVLHTQGVEFG : 99
P37114.pro : MDl6L6EK L6 F A a 6SkLRG44fKLPPGP P6P6FGnWLQVGDDLNhRNL d A44FG 6f6LRMGQRNLVVVSSP LaKeVLHTQgVEFG

 * 120 * 140 * 160 * 180 * 200
Q43240.pro : SRTRNVVFDIFTGKGQDMVFTVYGEHWRKMRRIMTVPFFTNKVVQQYRTGWEAEAAAVVDDVKKNPKAATEGVVIRKRLQLMMYNNMFRIMFDRRFESED : 199
P48522.pro : SRTRNVVFDIFTGKGQDMVFTVYGEHWRKMRRIMTVPFFTNKVVQQYRYGWEEEAARVVEDVKKNPESATNGIVLRRRLQLMMYNNMYRIMFDRRFESED : 199
Q04468.pro : SRTRNVVFDIFTGKGQDMVFTVYGEHWRKMRRIMTVPFFTNKVVQQYRYGWEAEAAAVVDDVKKNPAAATEGIVIRRRLQLMMYNNMFRIMFDRRFESED : 199
Q42797.pro : SRTRNVVFDIFTGKGQDMVFTVYGEHWRKMRRIMTVPFFTNKVVQQYRHGWESEAAAVVEDVKKNPDAAVSGTVIRRRLQLMMYNNMYRIMFDRRFESEE : 199
Q43054.pro : SRTRNVVFDIFTGKGQDMVFTVYGEHWRKMRRIMTVPFFTNKVVQQYRYGWEEEAAQVVEDVKKNPEAATNGIVLRRRLQLMMYNNMYRIMFDRRFESED : 199
O24312.pro : SRTRNVVFDIFTGKGQDMVFTVYGEHWRKMRRIMTVPFFTNKVVQQYRYGWEEEAAQVVEDVKKNPGAATHGIVLRRRLQLMMYNNMYRIMFDRRFESEE : 199
Q96423.pro : SRTRNVVFDIFTGKGQDMVFTVYGEHWRKMRRIMTVPFFTNKVVQQYRFGWESEAASVVDDVRRNPDAAAGGIVLRRRLQLMMYNNMYRIMFDRRFESEE : 199
P37115.pro : SRTRNVVFDIFTGEGQDMVFTVYGEHWRKMRRIMTVPFFTNKVVQQYRHGWEAEAAAVVDDVRKNPDAAVSGLVIRRRLQLMMYNNMYRIMFDRRFESEE : 199
Q43033.pro : SRTRNVVFDIFTGKGQDMVFTVYSEHWRKMRRIMTVPFFTNKVVQQYRFGWEDEAARVVEDVKANPEAATNGIVLRNRLQLLMYNNMYRIMFDRRFESVD : 200
P37114.pro : SRTRNVVFDIFTGKGQDMVFTVYGEHWRKMRRIMTVPFFTNKVVQQYRYGWESEAESVVNDVKNNAEASVGGIVIRKRLQLMMYNIMYRIMFDRRFESEE : 199
TR8942|c0_ : SRTRNVVFDIFTGKGQDMVFTVYGEHWRKMRRIMTVPFFTNKVVQQYRFGWEDEITKVVEDVRKMPEAATNGIVLRKRLQLMMYNNMYRIMFDRRFESEE : 199
 SRTRNVVFDIFTGkGQDMVFTVYgEHWRKMRRIMTVPFFTNKVVQQYR GWE Eaa VV DV4 np aa G V6R RLQL6MYNnM5RIMFDRRFESe

 * 220 * 240 * 260 * 280 * 300
Q43240.pro : DPLFVKLKMLNGERSRLAQSFEYNYGDFIPILRPFLKGYLKLCKEVKEKRFQLFKDYFVDERKKLGSTKSMDNNQ-LKCAIDHILDAKDKGEINEDNVLY : 298
P48522.pro : DPLFVKLKALNGERSRLAQGFEYNYGDFIPILRPFLRGYLRICKEVKERRLQLFKDYFVDERKKFGSTKSMDNNS-LKCAIDHILEAQQKGEINEDNVLY : 298
Q04468.pro : DPLFLKLKALNGERSRLAQSFEYNYGDFIPILRPFLRNYLKLCKEVKDKRIQLFKDYFVDERKKIGSTKKMDNNQ-LKCAIDHILEAKEKGEINEDNVLY : 298
Q42797.pro : DPIFQRLRALNGERSRLAQSFEYNYGDFIPILRPFLKGYLKICKEVKETRLKLFKDYFVDERKKLGSTKSTNNNNELKCAIDHILDAQRKGEINEDNVLY : 299
Q43054.pro : DPLFNKLKALNGERSRLAQSFDYNYGDFIPILRPFLRGYLKICQEVKERRLQLFKDYFVDERKKLASTKNMSNEG-LKCAIDHILDAQKKGEINEDNVLY : 298
O24312.pro : DPLFNKLKALNGERSRLAQSFDYNYGDFIPILRPFLRGYLKICQEVKERRLQLFKDYFVDERKKLASTKNMCNEG-LKCAIDHILDAQKKGEINEDNVLY : 298
Q96423.pro : DPLFVKLKALNGERSRLAQSFEYNYGDFIPILRPFLKGYLKICKEVKERRLKLFKDYFVDERMKLESTKSTSNEG-LKCAIDHILDAQKKGEINEDNVLY : 298
P37115.pro : DPLFQRLKALNGERSRLAQSFEYNYGDFIPILRPFLKGYLKICKEVKETRLKLFKDYFVDERKNIGSTKSTNNEG-LKCAIDHILDAEKKGEINEDNVLY : 298
Q43033.pro : DPLFLKLKALNGERSRLAQSFEYHFGDFIPILRPFLRGYLKLCQEIKDKRLKLFKDYFVDERKKLESIKSVDNNS-LKCAIDHIIEAQQKGEINEDNVLY : 299
P37114.pro : DPLFVKLKALNGERSRLAQSFEYNYGDFIPILRPFLKGYLKVCKEVKDRRLQLFKDYFVDERKKLESTKSTTSNDGLKCAIDHILDAQKKGEINDDNVLY : 299
TR8942|c0_ : DPLFLKLKALNGERSRLAQSFDYNYGDFIPILRPLLRGYLKICKEVKERRLQLFKDYFLDERKRLTSTTTPSNAG-LKCAIDHIIDAQQKGEINEDNVLY : 298
 DP6F 4L4aLNGERSRLAQsF Yn5GDFIPILRPfL4gYL46C E6K R LFKDYF6DERk Stk n LKCAIDHI6 A KGEINeDNVLY

 * 320 * 340 * 360 * 380 * 400
Q43240.pro : IVENINVAAIETTLWSIEWAIAELVNHPEIQAKLRHELVSQLGPGVQVTEPDLHKLPYLQAVIKETLRLRMAIPLLVPHMNLHDAKLGGYDIPAESKILV : 398
P48522.pro : IVENINVAAIETTLWSIEWGIAELVNHPEIQKKLRDELETVLGPGVQITEPDTYKLPYLQAVIKETLRLRMAIPLFLPHMNLHDAKLGGYDIPAESKILV : 398
Q04468.pro : IVENINVAAIETTLWSIEWGIAELVNHPEIQAKLRHELDTKLGPGVQITEPDVQNLPYLQAVVKETLRLRMAIPLLVPHMNLHDAKLGGFDIPAESKILV : 398
Q42797.pro : IVENINVAAIETTLWSIEWGIAELVNHPEIQQKLRDEIDRVLGAGHQVTEPDIQKLPYLQAVVKETLRLRMAIPLLVPHMNLHDAKLGGYDIPAESKILV : 399
Q43054.pro : IVENINVAAIETTLWSIEWGIAELVNHPEIQKKLRHELDTLLGPGHQITEPDTYKLPYLNAVVKETLRLRMAIPLLVPHMNLHDAKLGGFDIPAESKILV : 398
O24312.pro : IVENINVAAIETTLWSIEWGIAELVNHPEIQKKLRHELDTLLGPGHQITEPDTYKLPYLNAVVKETLRLRMAIPLLVPHMNLHDAKLGGFDIPAESKILV : 398
Q96423.pro : IVENINVAAIETTLWSIEWGIAELVNHPEIQKKVRDEIDRVLGPGHQVTEPDMQKLPYLQAVIKETLRLRMAIPLLVPHMNLHDAKLGGYDIPAESKILV : 398
P37115.pro : IVENINVAAIETTLWSIEWGIAELVNHPEIQQKVRDEIDRVLGVGHQVTEPDIQKLPYLQAVVKETLRLRMAIPLLVPHMNLHDAKLGGYDIPAESKILV : 398
Q43033.pro : IVENINVAAIETTLWSIEWGIAELVNNPEIQKKLRHELDTVLGAGVQICEPDVQKLPYLQAVIKETLRYRMAIPLLVPHMNLHDAKLAGYDIPAESKILV : 399
P37114.pro : IVENINVAAIETTLWSIEWGIAELVNHQDIQNKVREEMDRVLGPGHQVTEPDLHKLPYLQAVIKETLRLRMAIPLLVPHMNLHDPKLNGFDIPAESKILV : 399
TR8942|c0_ : IVENINVAAIETTLWSIEWGIAELVNNPKIQQRLREELDANLGPGVPITEPDTYKLPYLQAVIKETLRLRMAIPLLVPHMNLNDAKLNGFDIAAESKILV : 398
 IVENINVAAIETTLWSIEWgIAELVNhpeIQ 46R E6d LG G q6tEPD kLPYLqAV6KETLRlRMAIPLl6PHMNLhDaKL G5DIpAESKILV

 * 420 * 440 * 460 * 480 * 500
Q43240.pro : NAWWLANNPDQWKKPEEFRPERFLEEESKVEANGNDFRYLPFGVGRRSCPGIILALPILGITIGRLVQNFELLPPPGQDKVDTTEKGGQFSLHILKHSTI : 498
P48522.pro : NAWFLANNPEHWKKPEEFRPERFLEEESKVEANGNDFRYLPFGVGRRSCPGIILALPILGITIGRLVQNFELLPPPGKSKIDTSEKGGQFSLHILKHSTI : 498
Q04468.pro : NAWWLANNPDQWKKPEEFRPERFLEEEAKVEANGNDFRYLPFGVGRRSCPGIILALPILGITIGRLVQNFELLPPPGQSKIDTDEKGGQFSLHILKHSTI : 498
Q42797.pro : NAWWLANNPAHWKKPEEFRPERFFEEESLVEANGNDFRYLPFGVGRRSCPGIILALPILGITLGRLVQNFELLPPPGQSQIDTSEKGGQFSLHILKHSTI : 499
Q43054.pro : NAWWLANNPAHWKNPEEFRPERFLEEGAKVEANGNDFRYLPFGVGRRSCPGIILALPILGITLGRLVQNFELLPPPGQSKIDTSEKGGQFSLHILKHSTI : 498
O24312.pro : NAWWLANNPAHWKNPEEFRPERFLEEEAKVEANGNDFRYLPFGVGRRSCPGIILALPILGITLGRLVQNFELLPPPGQSKIDTSEKGGQFSLHILKHSTI : 498
Q96423.pro : NAWWLANNPANWKRPEEFRPERFLEEESHVEANGNDFRYLPFGVGRRSCPGIILALPILGITLGRLVQNFELLPPPGQSKLDTAEKGGQFSLHILKHSTI : 498
P37115.pro : NAWWLANNPAHWKKPEEFRPERFFEEESHVEANGNDFRYLPFGVGRRSCPGIILALPILGITLGRLVQNFELLPPPGQSQIDTSEKGGQFSLHILKHSTV : 498
Q43033.pro : NAWWLANNPAHWNKPDEFRPERFLEEESKVEANGNDFKYIPFGVGRRSCPGIILALPILGIVIGRLVQNFELLPPPGQSKIDTAEKGGQFSLQILKHSTI : 499
P37114.pro : NAWWLPNNPAHWKKPEEFRPERFLEEESHVEANGNDFRYLPFGVGRRSCPGIILALPILGITIGRLVQNVELLPPPGQSKIDTSEKGGQFSLHILKHSTI : 499
TR8942|c0_ : NAWWLANNPEHWKNPEEFRPERFLEEESKVEANGNDFRYLPFGVGRRSCPGIILALPILGITLGRLVQNFELLPPPGQEKLDTTEKGGQFSLHILKHSTI : 498
 NAW5LaNNP Wk PeEFRPERFlEEe VEANGNDF4Y6PFGVGRRSCPGIILALPILGIt6GRLVQNfELLPPPGqsk6DT EKGGQFSLhILKHST6


Q43240.pro : VAKPRVL- : 505
P48522.pro : VLKPRTF- : 505
Q04468.pro : VAKPRSF- : 505
Q42797.pro : VAKPRSF- : 506
Q43054.pro : VAKPRSF- : 505
O24312.pro : VAKPRSF- : 505
Q96423.pro : VAKPRSF- : 505
P37115.pro : VAKPRSF- : 505
Q43033.pro : VCKPRSL- : 506
P37114.pro : VAKPRSF- : 506
TR8942|c0_ : VAKPRVF- : 505
 VaKPR f


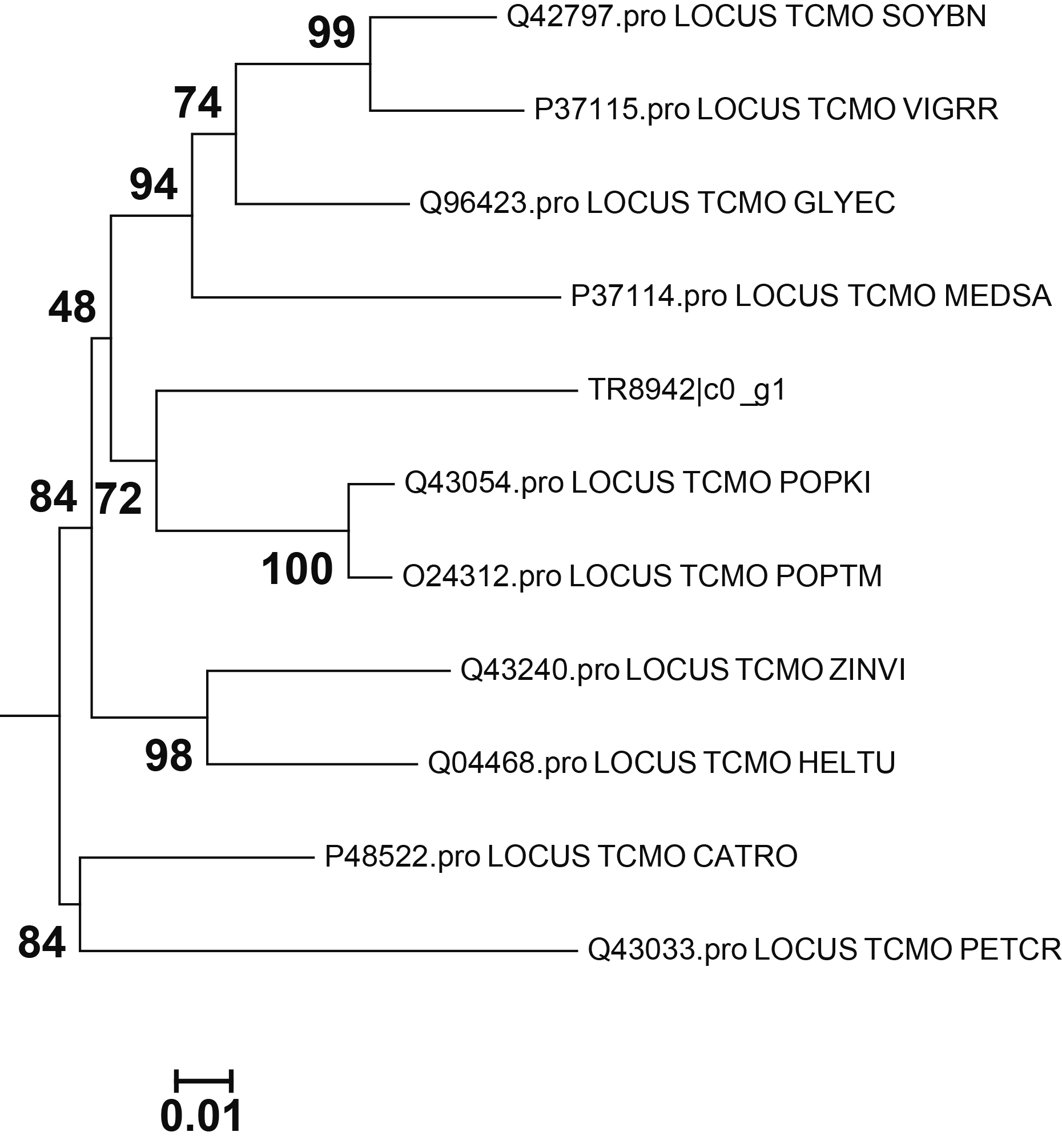

Supplement: S15 Fig — (DOCX) [file pone.0182348.s029.docx]
