# Supplementary material for: Understanding the molecular mechanisms underlying the effects of light intensity on flavonoid production by RNA-seq analysis in Epimedium pseudowushanense B.L.Guo
Source: PLoS One. 2017 Aug 7;12(8):e0182348. doi: 10.1371/journal.pone.0182348 (PMC5546586; doi:10.1371/journal.pone.0182348)

**S16 Fig. Sequence alignment of shikimate O-hydroxycinnamoyltransferase proteins from *E. pseudowushanense* and various other plants, and phylogenetic relationships of shikimate O-hydroxycinnamoyltransferase proteins from *E. pseudowushanense* and various other plants.**

* 20 * 40 * 60 * 80 * 100
XP_0102655 : --------------------------------------------------------------------MIISVKESTMVRPAQETPQRSLWNSNVDLVVP : 32
XP_0022689 : --------------------------------------------------------------------MIINVRESTMVRPAEETPRRSLWNSNVDLVVP : 32
XP_0166888 : ---MHWNTS-------------------FSLKKK----------TQRPEKAKSKPILFPPVDPTSTTTMIVNVKESTMVRPAGETPRRSLWNANVDLVVP : 68
XP_0124454 : MELSIWFRF-------------------FGRKMIGVVNVISLIFNNTLNQAKSKPILFPPVDPTSTTTMIVNVKESTMVRPAGETPRRSLWNANVDLVVP : 81
NP_0013139 : MPLLFALPFSTLILFQVLLQSSPQCSPPFSPTPLPFFCSSSSFAFTSDLQAKSKPILFPPVDPTSTTTMIVNVKESTMVRPAGETPRRSLWNANVDLVVP : 100
XP_0166888 : --------------------------------------------------------------------MIVNVKESTMVRPAGETPRRSLWNANVDLVVP : 32
XP_0124454 : --------------------------------------------------------------------MIVNVKESTMVRPAGETPRRSLWNANVDLVVP : 32
ACC63882.p : --------------------------------------------------------------------MIINVKESTMVQPAEETPRRGLWNSNVDLVVP : 32
XP_0110205 : --------------------------------------------------------------------MIINVKESTMVQPAEETPRRRLWNSNVDLVVP : 32
TR465|c1_g : --------------------------------------------------------------------MIINVKESTMVTPAEETPKTNLWNSNVDLVVP : 32
 MI6nV4ESTMV PA ETP r LWN NVDLVVP

 * 120 * 140 * 160 * 180 * 200
XP_0102655 : RMHTPSVYFYRPTGSTNFFDPKVLKEALSKALVPFYPMAGRLKRDEDGRIEINCNGEGVLFVEAETGSVIDDFGDFAPTMELKQLIPAVDYSGDISSYPL : 132
XP_0022689 : RMHTPSVYFYRPSGAANFFDPQVMKEALSKVLVPFYPMAGRLRRDEDGRIEIDCNAEGVLFVEADTGSVIDDFGDFAPTLELRQLIPTVDYSGDIGSYSL : 132
XP_0166888 : RFHTPSVYFYRPNGAANFFDPQVMKEALSKALVPFYPMAGRLKRDEDGRIEIDCNGEGVLFVEAETNSVIDDFGDFAPTLELRQLIPTVDYSGGISTYPL : 168
XP_0124454 : RFHTPSVYFYRPNGAANFFDPQVMKEALSKALVPFYPMAGRLKRDEDGRIEIDCNGEGVLFVEAETNSVIDDFGDFAPTLELRQLIPTVDYSGGISTYPL : 181
NP_0013139 : RFHTPSVYFYRPNGAANFFDPQVMKEALSKALVPFYPMAGRLKRDEDGRIEIDCNGEGVLFVEAETNSVIDDFGDFAPTLELRQLIPTVDYSGGISTYPL : 200
XP_0166888 : RFHTPSVYFYRPNGAANFFDPQVMKEALSKALVPFYPMAGRLKRDEDGRIEIDCNGEGVLFVEAETNSVIDDFGDFAPTLELRQLIPTVDYSGGISTYPL : 132
XP_0124454 : RFHTPSVYFYRPNGAANFFDPQVMKEALSKALVPFYPMAGRLKRDEDGRIEIDCNGEGVLFVEAETNSVIDDFGDFAPTLELRQLIPTVDYSGGISTYPL : 132
ACC63882.p : RFHTPSVYFYRPTGASNFFDAKVLKEALSKALVPFYPMAGRLRRDDDGRIEIDCNAEGVLFVEAGTASVVADFGDFAPTLELKQLIPTVDYSGGISTYPL : 132
XP_0110205 : RFHTPSVYFYRPTGASNFFDAKVLKEALSKALVPFYPMAGRLRRDDDGRIEIDCNAEGVLFVEAETASVVADFGDFAPTLELKQLIPTVDYSGGISTYPL : 132
TR465|c1_g : RMHTPSVYFYRPNGTSSFFDSKVLKESLSKALVPFYPMAARLKRDEDGRIEIDCGGQGVLFVVAETGSVIDDFGDFAPTLELRQLIPAVDYSGDISSYPL : 132
 R HTPSVYFYRP Ga nFFD V6KEaLSKaLVPFYPMAgRL4RDeDGRIEI1Cn 2GVLFVeAeT SV6dDFGDFAPT6EL4QLIPtVDYSG Is3YpL

 * 220 * 240 * 260 * 280 * 300
XP_0102655 : LVLQITRFKCGGVSLGVGMQHHVADGSSGLHFINTWSDMARGLDLTIPPFIDRTLLRARDPPTPSFHHIEYQPAPPMKHPPETPNSQSGPESTTVTMFKI : 232
XP_0022689 : LILQVTHFKCGGVSLGVGMQHHVADGASGLHFINTWSDMARGLDITIPPFIDRTLLRARDPPQPAFHHIEYQPPPQLKTPLPN------TQNTNVCIFRI : 226
XP_0166888 : LVLQVTYFKCGGASLGVGMQHHAADGYSGLHFINTWSDMVRGLDLTIPPFIDRTLLRARDPPQPVFHHVEYQPPPAMKIPPQS----TGPESTAISIFKL : 264
XP_0124454 : LVLQVTYFKCGGASLGVGMQHHAADGYSGLHFINTWSDMARGLDLTIPPFIDRTLLRARDPPQPVFHHVEYQPPPAMKIPPQS----TGPESTAISIFKL : 277
NP_0013139 : LVLQVTYFKCGGASLGVGMQHHAADGYSGLHFINTWSDMARGLDLTIPPFIDRTLLRARDPPQPVFHHVEYQPPPAMKIPPQS----TGPESTAISIFKL : 296
XP_0166888 : LVLQVTYFKCGGASLGVGMQHHAADGYSGLHFINTWSDMVRGLDLTIPPFIDRTLLRARDPPQPVFHHVEYQPPPAMKIPPQS----TGPESTAISIFKL : 228
XP_0124454 : LVLQVTYFKCGGASLGVGMQHHAADGYSGLHFINTWSDMARGLDLTIPPFIDRTLLRARDPPQPVFHHVEYQPPPAMKIPPQS----TGPESTAISIFKL : 228
ACC63882.p : LVLQVTYFKCGGVSLGVGMQHHAADGFSGLHFVNTWSDMARGLDLTIPPFIDRTLLRARDPPQPAFHHVEYQPPPAMKTVLET----SKPESTAVSIFKL : 228
XP_0110205 : LVLQVTHFKCGGVSLGVGMQHHAADGFSGLHFVNTWSDMARGLDLTIPPFIDRTLLRARDPPQPAFHHVEYQPPPAMKAVPET----SKPESTAVSIFKL : 228
TR465|c1_g : LVLQVTHFKCGGVSLGVGMQHHVADGASGLHFINTWSDMARGLDLTIPPFIDRTLLRARDTPKPVFKHVEYQPPPPMKIPISQP----APTEASVAIFKM : 228
 L6LQ6T FKCGG SLGVGMQHH ADG SGLHF6NTWSDMaRGLD6TIPPFIDRTLLRARDpPqP FhH6EYQPpP 6K p p st 6 6F46

 * 320 * 340 * 360 * 380 * 400
XP_0102655 : SRDQLNILKAKSKDGANSVNYSSYEMLAGHVWRCVCKARGLPDDQDTKLYIATDGRSRLRPPLPPGYFGNGIFTATPIAVSGDLLSKPLTYAAGRIHDAL : 332
XP_0022689 : TRDQLNTLKNKSKEDGNTISYSSYVMLAGHVWRCACKARSLPADQDSKMYIATDGRSRLRPALPPGYFGNVIFTTTPVAVAGELMSKPLWYAASKIHNAL : 326
XP_0166888 : TRDQLNALKAKCKEDGNDVNYSSYEMLSGHVWRSVCKARGLEDDQGTKLYIATDGRARLRPPLPPGYFGNVIFTATPIAVAGDLLSKPTWYAASRIHDAL : 364
XP_0124454 : TRDQLNALKAKCKEDGNDVNYSSYEMLSGHVWRSVCKARGLEDDQGTKLYIATDGRARLRPPLPPGYFGNVIFTATPIAVAGDLLSKPTWYAASRIHDAL : 377
NP_0013139 : TRDQLNALKAKCKEDGNDVNYSSYEMLSGHVWRSVCKARGLEDDQGTKLYIATDGRARLRPPLPPGYFGNVIFTATPIAVAGDLLSKPTWYAASRIHDAL : 396
XP_0166888 : TRDQLNALKAKCKEDGNDVNYSSYEMLSGHVWRSVCKARGLEDDQGTKLYIATDGRARLRPPLPPGYFGNVIFTATPIAVAGDLLSKPTWYAASRIHDAL : 328
XP_0124454 : TRDQLNALKAKCKEDGNDVNYSSYEMLSGHVWRSVCKARGLEDDQGTKLYIATDGRARLRPPLPPGYFGNVIFTATPIAVAGDLLSKPTWYAASRIHDAL : 328
ACC63882.p : TRDQLNTLKAKAKEGGNNIGYSSYEMLAGHVWRSACKARGLPDDQETKLYIATDGRSRLRPTLPPGYFGNVIFTATPIAVAGEIQSKPTWYAAGKIHDSL : 328
XP_0110205 : TRDQLNTLKAKAKEGGNNIGYSSYEMLAGHVWRSACQARGLPDDQETKLYIATDGRSRLRPTLPPGYFGNVIFTATPIAVAGEIQSKPTWYAAGKIHDSL : 328
TR465|c1_g : TRDQLNTLKGKSKEGGNTVNYSSYEMLAGHVWRSVCKARGLPEDQETKMYIATDGRSRLRPALPPGYFGNVIFTTTPIAVSGDLNSKPLTYAASVIHNAL : 328
 3RDQLN LKaK Ke gN 6 YSSYeML GHVWRs CkARgL dDQ 3K6YIATDGR RLRP LPPGYFGNvIFTaTP6AVaG 6 SKP wYAA IH1aL

 * 420 * 440 * 460 * 480 * 500
XP_0102655 : VRMDDEYLRSALDYLELQPDLTVLVRGAHTFRCPNIGITSWVRLPIHDADFGWGRPIFMGPGGIAYEGLAFLLPSPVNDGNLSLAISLQSDHMKVFQKIL : 432
XP_0022689 : ARMDDEYLRSALDYLELQPDLTALVRGAHTFRCPNIGITSWTRLPIYDADFGWGRPIFMGPGGIALEGLAFALPSPTNDGSLSIAISLQEDHMKLFQKYL : 426
XP_0166888 : VRMDDEYLRSALDYLELQPDLSALVRGAHTFRCPNLGITSWVRLPIHDADFGWGRPIFMGPGGIPYEGLSFVIPSPNNDGSLSVAISLQTEHMKVFEKLF : 464
XP_0124454 : VRMDDEYLRSALDYLELQPDLSALVRGAHTFRCPNLGITSWVRLPIHDADFGWGRPIFMGPGGIPYEGLSFVLPSPNNDGSLSVAISLQTEHMNVFEKLF : 477
NP_0013139 : VRMDDEYLRSALDYLELQPDLSALVRGAHTFRCPNLGITSWVRLPIHDADFGWGRPIFMGPGGIPYEGLSFVLPSPNNDGSLSVAISLQTEHMNVFEKLF : 496
XP_0166888 : VRMDDEYLRSALDYLELQPDLSALVRGAHTFRCPNLGITSWVRLPIHDADFGWGRPIFMGPGGIPYEGLSFVIPSPNNDGSLSVAISLQTEHMKVFEKLF : 428
XP_0124454 : VRMDDEYLRSALDYLELQPDLSALVRGAHTFRCPNLGITSWVRLPIHDADFGWGRPIFMGPGGIPYEGLSFVLPSPNNDGSLSVAISLQTEHMNVFEKLF : 428
ACC63882.p : VRMDNDYLRSALDFLELQPDLSALVRGAHTFRCPNLGITSWVRLPIHDADFGWGRPIFMGPGGIAYEGLSFIIPSSTNDGSLSVAISLQAEHMKLFEKFI : 428
XP_0110205 : VRMDNDYLRSALDFLELQPDLSALVRGAHTFRCPNLGITSWVRLPIHDADFGWGRPIFMGPGGIAYEGLSFIIPSSTNDGSMSVAISLQAEHMKLFEKFM : 428
TR465|c1_g : VRMDDEYMRSALDYLELQPDLSALVRGAHTFRCPNIGITSWSRLPIHDADFGWGRPIFMGPGGIAYEGLAFILPSPNGDGSLALAISLQPDHMKLFEKFI : 428
 vRMD1eY6RSALD5LELQPDL3aLVRGAHTFRCPN6GITSWvRLPIhDADFGWGRPIFMGPGGI yEGL F 6PSp nDGs6s6AISLQ HM 6F2K


XP_0102655 : YDF-- : 435
XP_0022689 : YEI-- : 429
XP_0166888 : YDI-- : 467
XP_0124454 : YDI-- : 480
NP_0013139 : YDI-- : 499
XP_0166888 : YDI-- : 431
XP_0124454 : YDI-- : 431
ACC63882.p : YDIKE : 433
XP_0110205 : YDI-- : 431
TR465|c1_g : YEI-- : 431
 Ydi


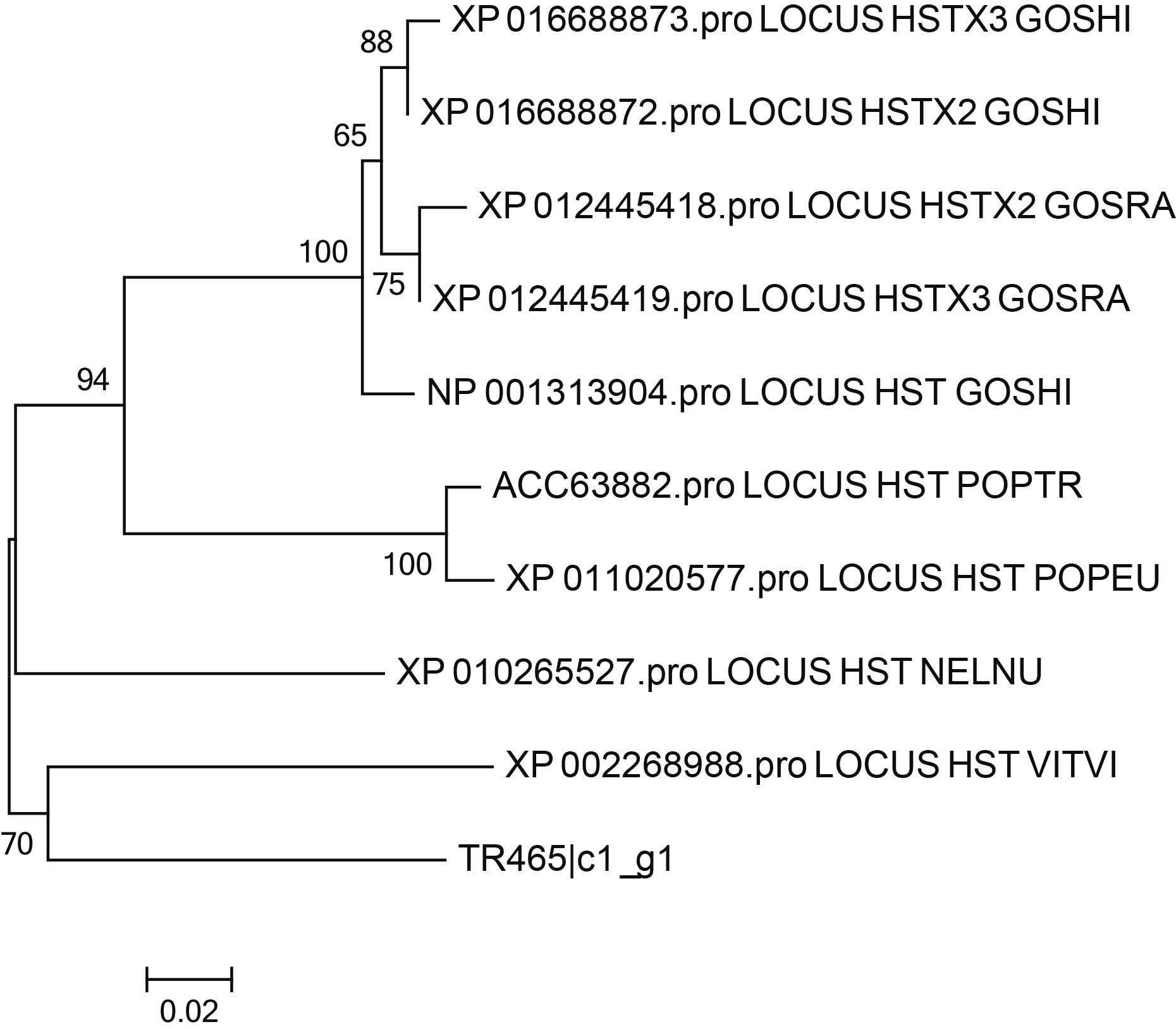

Supplement: S16 Fig — (DOCX) [file pone.0182348.s030.docx]
