# Supplementary material for: Understanding the molecular mechanisms underlying the effects of light intensity on flavonoid production by RNA-seq analysis in Epimedium pseudowushanense B.L.Guo
Source: PLoS One. 2017 Aug 7;12(8):e0182348. doi: 10.1371/journal.pone.0182348 (PMC5546586; doi:10.1371/journal.pone.0182348)

**S17 Fig. Sequence alignment of coumaroylquinate(coumaroylshikimate) 3'-monooxygenase proteins from *E. pseudowushanense* and various other plants, and phylogenetic relationships of coumaroylquinate(coumaroylshikimate) 3'-monooxygenase proteins from *E. pseudowushanense* and various other plants.**

* 20 * 40 * 60 * 80 * 100
O48922.pro : --MALLLIIPISLVTLWLGYTLYQRLRF-KLPPGPRPWPVVGNLYDIKPVRFRCFAEWAQSYGPIISVWFGSTLNVIVSNSELAKEVLKEHDQLLADRHR : 97
O22203.pro : --MSWFLIA-VATIAAVVSYKLIQRLRY-KFPPGPSPKPIVGNLYDIKPVRFRCYYEWAQSYGPIISVWIGSILNVVVSSAELAKEVLKEHDQKLADRHR : 96
O48956.pro : MDASLLLSVALAVVLIPLSLALLNRLRLGRLPPGPRPWPVLGNLRQIKPIRCRCFQEWAERYGPVISVWFGSGLTVVVSTSELAKEVLKENDQQLADRPR : 100
Q9CA60.pro : --MDLLLIS--LTTIIIAAYMQNLRRRGSNIPPGPPTRFLVGNLHQLKPLWTQSFSEWSQTYGPIISVWLGSQLAVVVSSSDLAKQVLRDKDYQLCNRHR : 96
Q9CA61.pro : --MIIYLIS--LLPIIVATLMLYQRWWRSNIPPGPKPKFLLGNLHQMKPLWTHSFSEWSETYGPIISVWIGSQLTVVVSSSDLARQVLRDKDHQLSNRHR : 96
TR1877|c0_ : --MALFLLF-LSPIFLLLAYNLYYHLRF-KLPPGPRPWPIVGNLYDIKPVRFRCFSEWAQTYGPIISVWFGSTLNVVVTNSELAKEVLKDKDQQLADRHR : 96
 m L l r r PPGP p 66GNL 6KP6 5 EW 2 YGP6ISVW GS L V6V3 s LA42VL4 D L 1RhR

 * 120 * 140 * 160 * 180 * 200
O48922.pro : SRSAAKFSRDGKDLIWADYGPHYVKVRKVCTLELFSPKRLEALRPIREDEVTSMVDSVYNHCTS---TENLGKGILLRKHLGVVAFNNITRLAFGKRFVN : 194
O22203.pro : NRSTEAFSRNGQDLIWADYGPHYVKVRKVCTLELFTPKRLESLRPIREDEVTAMVESVFRDCNL---PENRAKGLQLRKYLGAVAFNNITRLAFGKRFMN : 193
O48956.pro : NRSTQRFSRNGQDLIWADYGPHYIKVRKLCNLELFTPKRLEALRPIREDEVTAMVESVYRAATA---PGNEGKPMVVRNHLSMVAFNNITRLAFGKRFMN : 197
Q9CA60.pro : ---TARMTQNGSDLIWSDYGAHYVKMRKLCTLELFSLKSIECFRSMREMEVSSMVKSIFNDFMS---DDQ--KPVVLRNYLDSVALNIVSRLVIGKTFEP : 188
Q9CA61.pro : ---IARMTQTGTDLVWSDYSPHYVKLRKLCTLELFSLKSIENFRSLREMEARSMVVSILKDLMSNSGDDQERKPVIVRKYLAAVVLNTISRLMIGKEFGS : 193
TR1877|c0_ : SRSAAKFSRDGTDLIWADYGPHYVKVRKVCTLELFSPKRLEGLRPIREDEVTAMVESIFNDYTK---PENYGKSVVVKSYLGAVAFNNITRLAFGKRFMN : 193
 3 G DL6W DYgpHY6K6RK6CtLELF3 K 6E R 6RE Ev MV S6 K 6 64 L Va N 63RL GK F

 * 220 * 240 * 260 * 280 * 300
O48922.pro : SEGVMDEQGVEFKAIVENGLKLGASLAMAEHIPWLRWMFP--LEEGAFAKHGARR-DRLTRAIMAEHTEARKKSGGAKQHFVDALLTLQDKYDLSEDTII : 291
O22203.pro : AEGVVDEQGLEFKAIVSNGLKLGASLSIAEHIPWLRWMFP--ADEKAFAEHGARR-DRLTRAIMEEHTLARQKSSGAKQHFVDALLTLKDQYDLSEDTII : 290
O48956.pro : ANGDIDEQGREFKTIVNNGIKIGASLSVAEFIWYLRWLCP--LNEELYKTHNERR-DRLTMKIIEEHAKSLKES-GAKQHFVDALFTLKQQYDLSEDTVI : 293
Q9CA60.pro : ----K--DGREFRSIVERETRLPGATKMLDYTVWLKRLSSWFTSDKAFMKHMARKRNWFKRAVM-DEVYGGRDQ----KCFVQSLLELKEKDELTEETVM : 277
Q9CA61.pro : ----E--EGKEFKAIVEKEHLLSGSGTILDHVWWLKWVSSWFFSDKEFLAHKDRRTKWFRGAIMVEEDIEIEDH----RGFVRKLLVLKEQKELSEETVG : 283
TR1877|c0_ : AEGVIDEQGLEFKAIVANGLKLGASLAMAEHIPWLRWMFP--LEEGAFAKHGARR-DNLTRAIMEEHTAARKKSGGAQQHFVDALLTLQEKYDLSEDTII : 290
 G EF4 IV 6 s 6 5L4w6 5 H R4 a66 e FV Ll L L3E T6

 * 320 * 340 * 360 * 380 * 400
O48922.pro : GLLWDMITAGMDTTAISVEWAMAELIRNPRVQQKVQEELDRVIGLERVMTEADFSNLPYLQCVTKEAMRLHPPTPLMLPHRANANVKVGGYDIPKGSNVH : 391
O22203.pro : GLLWDMITAGMDTTAITAEWAMAEMIKNPRVQQKVQEEFDRVVGLDRILTEADFSRLPYLQCVVKESFRLHPPTPLMLPHRSNADVKIGGYDIPKGSNVH : 390
O48956.pro : GLLWDMITAGMDTTVISVEWAMAELVRNPRVQKKLQEELDRVVGRDRVMLETDFQNLPYLQAVVKESLRLHPPTPLMLPHKASTNVKIGGYDIPKGANVM : 393
Q9CA60.pro : GLVWNMLTAGADTTAITIEWAMAEMIRCPTVKEKVQDELDSVVGSGRLMSDADIPKLPFLQCVLKEALRLHPPTPLMLPHKASESVQVGGYKVPKGATVY : 377
Q9CA61.pro : GLVWNMLTAGADTTAVVIEWAMAEMIKCPTVQEKAQQELDSVVGSERLMTESDIPILPYLQCVVKEALRLHPSTPLMLPHKASETVWVGGYKVPKGATVY : 383
TR1877|c0_ : GLLWDMITAGMDTTAISVEWAMAELIKNPRVQEKAQEELDRVIGFERVVTEPDFTNLPYLQCIAKEALRLHPPTPLMLPHRANANVKIGGYDVPKGSNMH : 390
 GL6W1M6TAG DTTa6 EWAMAE664 P Vq K Q ElD V6G R66 e D LP5LQc6 KE RLHPpTPLMLPH4a V 6GGY 6PKG 6

 * 420 * 440 * 460 * 480 * 500
O48922.pro : VNVWAVARDPAVWKDPLEFRPERFLEEDVDMKGHDFRLLPFGSGRRVCPGAQLGINLAASMLGHLLHHFCWTPPEGMKPEEIDMGENPGLVTYMRTPIQA : 491
O22203.pro : VNVWAVARDPAVWKNPFEFRPERFLEEDVDMKGHDFRLLPFGAGRRVCPGAQLGINLVTSMMSHLLHHFVWTPPQGTKPEEIDMSENPGLVTYMRTPVQA : 490
O48956.pro : VNVWAVARDPKVWSNPLEYRPERFLEENIDIKGSDFRVLPFGAGRRVCPGAQLGINLVASMIGHLLHHFEWSLPEGTRPEDVNMMESPGLVTFMGTPLQA : 493
Q9CA60.pro : VNVQAIARDPANWSNPDEFRPERFLVEETDVKGQDFRVLPFGSGRRVCPAAQLSLNMMTLALGSLLHCFSWTSS--TPREHIDMTEKPGLVCYMKAPLQA : 475
Q9CA61.pro : VNVQAIGRDPANWINPYEFRPERFLQEETDVKGRDFRVLPFGSGRRMCPAAQLSMNLMTLVMGNLLHCFSWSSP--VPGERIDMSENPGLLCNMRTPLQA : 481
TR1877|c0_ : VNVWAVARDPAVWKEPLEFRPERFMEEDVDMKGHDYRLLPFGAGRRVCPGAQLGINLVTSMLGHLLHHFCWTPPEGVRPEDIDMSENPGMVTYMTTPLQA : 490
 VNV A6aRDPa W P E5RPERF6 E D6KG D5R6LPFG GRR6CP AQL 6N6 6g LLH F W3 p E 61M E PG66 M tP6QA

 * 520
O48922.pro : VVSPRLP-SHLYKRVPAEI- : 509
O22203.pro : VATPRLP-SDLYKRVPYDM- : 508
O48956.pro : VAKPRLEKEELYNRVPVEM- : 512
Q9CA60.pro : LASSRLP----QELYL---- : 487
Q9CA61.pro : LALPRAA----ARAIPLPLD : 497
TR1877|c0_ : VPTPRLP-THLYKRMAVDM- : 508
 6 pRl


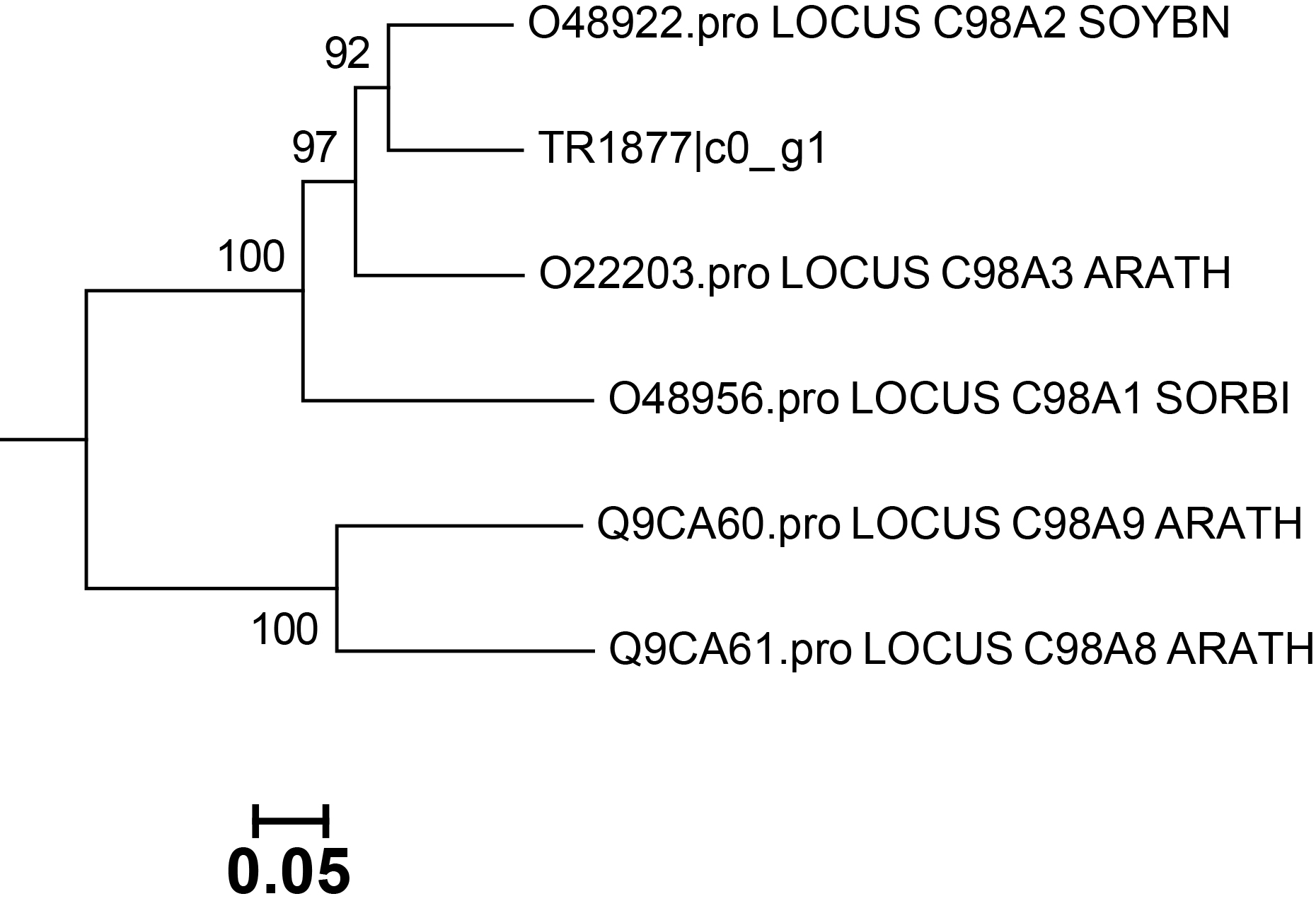

Supplement: S17 Fig — (DOCX) [file pone.0182348.s031.docx]
