# Supplementary material for: Understanding the molecular mechanisms underlying the effects of light intensity on flavonoid production by RNA-seq analysis in Epimedium pseudowushanense B.L.Guo
Source: PLoS One. 2017 Aug 7;12(8):e0182348. doi: 10.1371/journal.pone.0182348 (PMC5546586; doi:10.1371/journal.pone.0182348)
Supplement: S18 Fig — The X-axis shows the range of lengths of the transcript sequences. The Y-axis shows the number of unigenes. (DOCX) [file pone.0182348.s032.docx]

**S18 Fig. Sequence length of TF unigenes. The X-axis shows the range of lengths of the transcript sequences. The Y-axis shows the number of unigenes.**
