# Supplementary material for: Understanding the molecular mechanisms underlying the effects of light intensity on flavonoid production by RNA-seq analysis in Epimedium pseudowushanense B.L.Guo
Source: PLoS One. 2017 Aug 7;12(8):e0182348. doi: 10.1371/journal.pone.0182348 (PMC5546586; doi:10.1371/journal.pone.0182348)

**S19 Fig. Distribution of TF family. The X-axis shows the type of transcription factor family. The Y-axis shows the number of unigenes.**


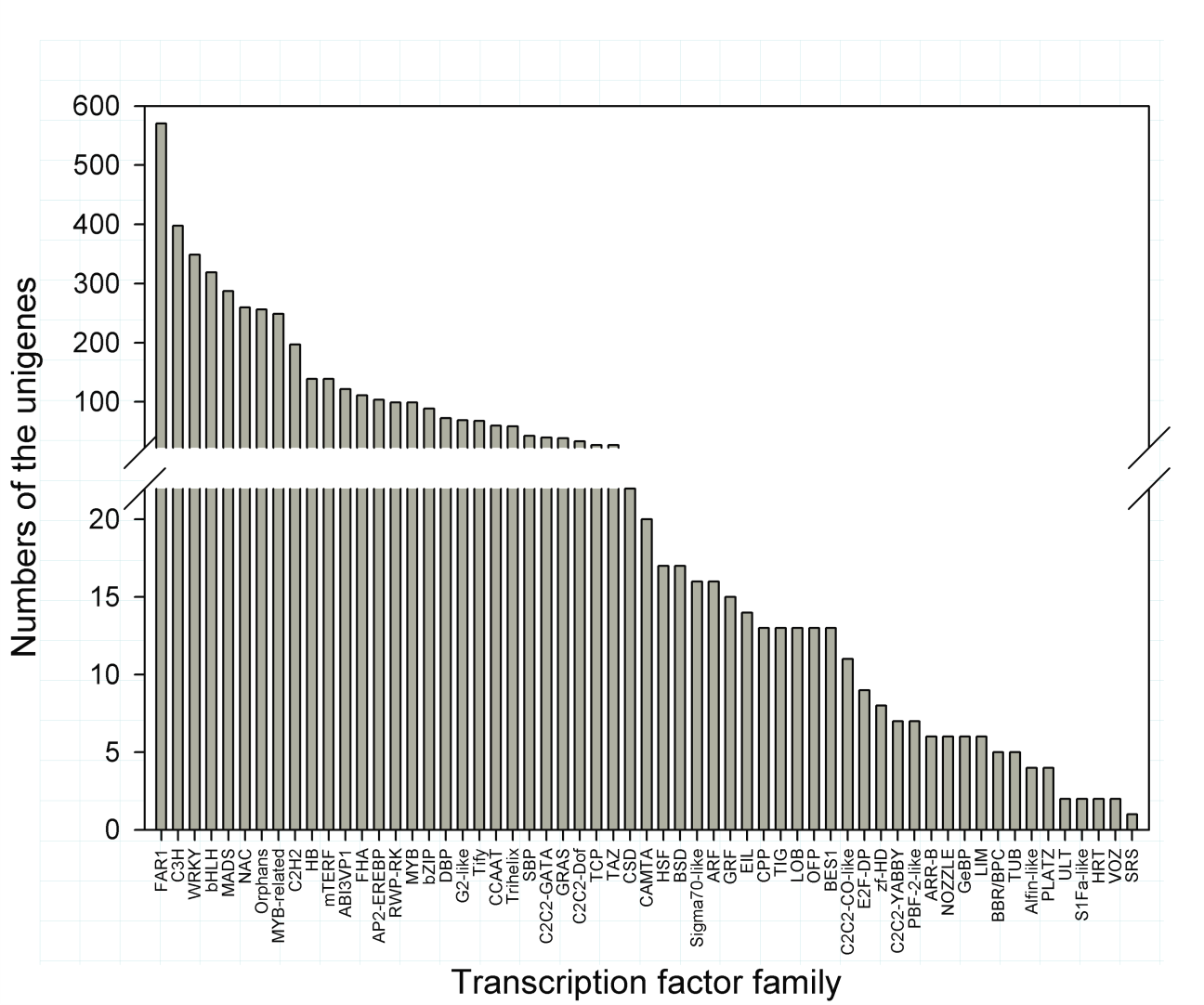

Supplement: S19 Fig — The X-axis shows the type of transcription factor family. The Y-axis shows the number of unigenes. (DOCX) [file pone.0182348.s033.docx]
